# Enhancing type 2 diabetes care by an individualized and group-based therapeutic patient education program: study protocol for a cluster randomized trial

**DOI:** 10.1186/s13063-025-09302-x

**Published:** 2025-12-23

**Authors:** David Ehlig, Maxime Sapin, Minette-Joëlle Zeukeng, Justus Vogel, Vincent Barthassat, Benoit Favre, Henrique DaCosta, Ksenia Tugay, Stephane Coendoz-Carron, David Bumann, Joëlle Coclet, Philippe Schaller, Alexander Geissler

**Affiliations:** 1 Chair of Health Economics, Policy and Management, School of Medicine, University of St. Gallen, St. Gallen, Switzerland; 2 Réseau Delta, Petit-Lancy, Switzerland; 3 Sokle SA, Geneve, Switzerland; 4 Groupe Mutuel, Martigny, Switzerland; 5 Commune de Savièse, Saviese, Switzerland; 6 Bern, Switzerland

**Keywords:** Patient therapeutic education, Cost-effectiveness, PROMs, Chronic disease management, Health-related quality of life, Type 2 diabetes, Value-based healthcare

## Abstract

**Background:**

Type 2 diabetes (T2D) presents a significant challenge to health systems and its prevalence is projected to increase. T2D is significantly influenced by lifestyle factors, including diet and physical activity. This makes therapeutic patient education (TPE) a pivotal component of T2D treatment strategies. While in some countries, like Germany and the UK, TPE elements like physician counselling regarding lifestyle changes (e.g., nutrition, smoking, exercise) and participation in diabetes education courses are part of structured nationwide disease management programs; in Switzerland, TPE elements are rarely part of the standard of care protocols. Our goal is to evaluate whether an interdisciplinary and individualized T2D program including TPE elements improves patients’ health outcomes.

**Methods:**

The study is a multi-center cluster randomized controlled trial in the canton of Geneva in Western Switzerland. We aim to include a minimum of 154 recently diagnosed T2D patients from around 30 primary care physician (PCP) practices. Practices are randomized with a 1:1 patient allocation ratio to either intervention or control group using covariate constrained randomization. The intervention lasts 12 months with a 6-month follow-up and consists of two steps. First, PCPs in the intervention group and associated healthcare professionals (e.g., dieticians, physical therapists) are trained in interprofessional group sessions on TPE elements by specialists. Second, patients follow an individualized treatment plan which is designed within an initial quality circle with their PCP, other healthcare professionals, a TPE specialist, a dedicated study manager, and other patients. The treatment plan is accompanied by regular patient-reported outcome measure (PROM) collections, which are discussed in patients’ regular PCP visits. Patients in the control group follow standard of care. Primary endpoint is the 12 months mean change in HbA1c levels, secondary endpoints are the 18 months mean change in HbA1c levels, the 12 and 18 months mean changes in patient-reported outcomes (EQ-5D-5L, DIAB-Q), mean changes in medical outcomes (blood pressure, body composition, medication intake), and patient experience. We further evaluate cost-effectiveness from the payer perspective.

**Discussion:**

A positive evaluation of the study can inform a wider roll-out of the T2D program within Switzerland and be a cornerstone for better patient health outcomes for T2D patients.

**Trial registration:**

ClinicalTrials.gov: NCT06774950. Registered on 14 January 2025

**Supplementary Information:**

The online version contains supplementary material available at 10.1186/s13063-025-09302-x.

## Introduction

### Background and rationale {6a}

Type 2 diabetes (T2D) presents a significant challenge to health systems and its prevalence is projected to increase continuously at least until 2030 [1]. T2D is a metabolic disorder, characterized by high blood sugar, insulin resistance, and eventually by relative insulin deficiency. T2D is significantly influenced by lifestyle factors, including diet, physical activity, and body weight [2]. Management of T2D, therefore, not only relies on medical interventions but also on patients’ engagement and lifestyle changes, making patient education a pivotal component of treatment strategies [3].

Structured T2D management programs with Therapeutic Patient Education (TPE) elements on the individual patient level and within group settings have shown to reduce glycated hemoglobin (HbA1c) levels in the short and medium term [4–8], leading to a reduced need for medication [5], partial or complete remission of T2D [7] and improved patients health-related quality of life (HRQoL) [9]. TPE usually consists of a set of educational interventions carried out by trained health professionals to support patients to self-manage their chronic conditions with the support of their caregivers and families.

Additionally, patient reported outcome measures (PROMs) are a promising tool to enhance T2D programs as they represent another source for empowering patients through understandable health status feedback and improved physician–patient communication [10, 11]. More concretely, physicians can monitor patients’ health status in a standardized way using PROMs. This way, physicians receive timely and structured insights on individual patient problem areas (e.g., distress, psychological well-being) enabling them to better personalize treatment strategies [12, 13].

While structured nationwide disease management programs for T2D exist in other countries (e.g., Germany [14], or the UK [8]), which include some TPE elements such as physician counselling regarding lifestyle changes (e.g., nutrition, smoking, exercise) and participation in diabetes education courses; in Switzerland, there is no standard on T2D management programs, and only a small share of patients received any form of TPE [15]. If any, the implementation of T2D programs including TPE elements is merely observed within practice networks and their effectiveness is evaluated in small pilot or case studies [15, 16].

In our study, we aim to evaluate the effect of an interdisciplinary and individualized T2D program including TPE elements on individual and group level to decrease patients’ HbA1c levels and to lead to a partial or full remission from T2D. We combine this program with regular measurements of patient-reported distress and psychological well-being that is to be discussed with the treating physician. For evaluation, we set up a cluster randomized controlled trial (CRT) in the canton Geneva in Western Switzerland. We further assess the cost-effectiveness of the program from a payer perspective.

### Objectives {7}

The primary objective of this CRT is to test whether an interdisciplinary and individualized T2D management program that includes TPE elements on an individual and group level combined with regular PROM collections and discussions reduces the glycated hemoglobin (A1c) (HbA1c) for T2D patients that were diagnosed within the last 10 years compared to standard care. Secondary objectives are the valuation of the program’s effect on other outcomes, such as health-related quality of life, blood pressure, or body composition and investigate whether cost-effectiveness allows for a wider regional or national roll-out.

### Trial design {8}

**Fig. 1:**
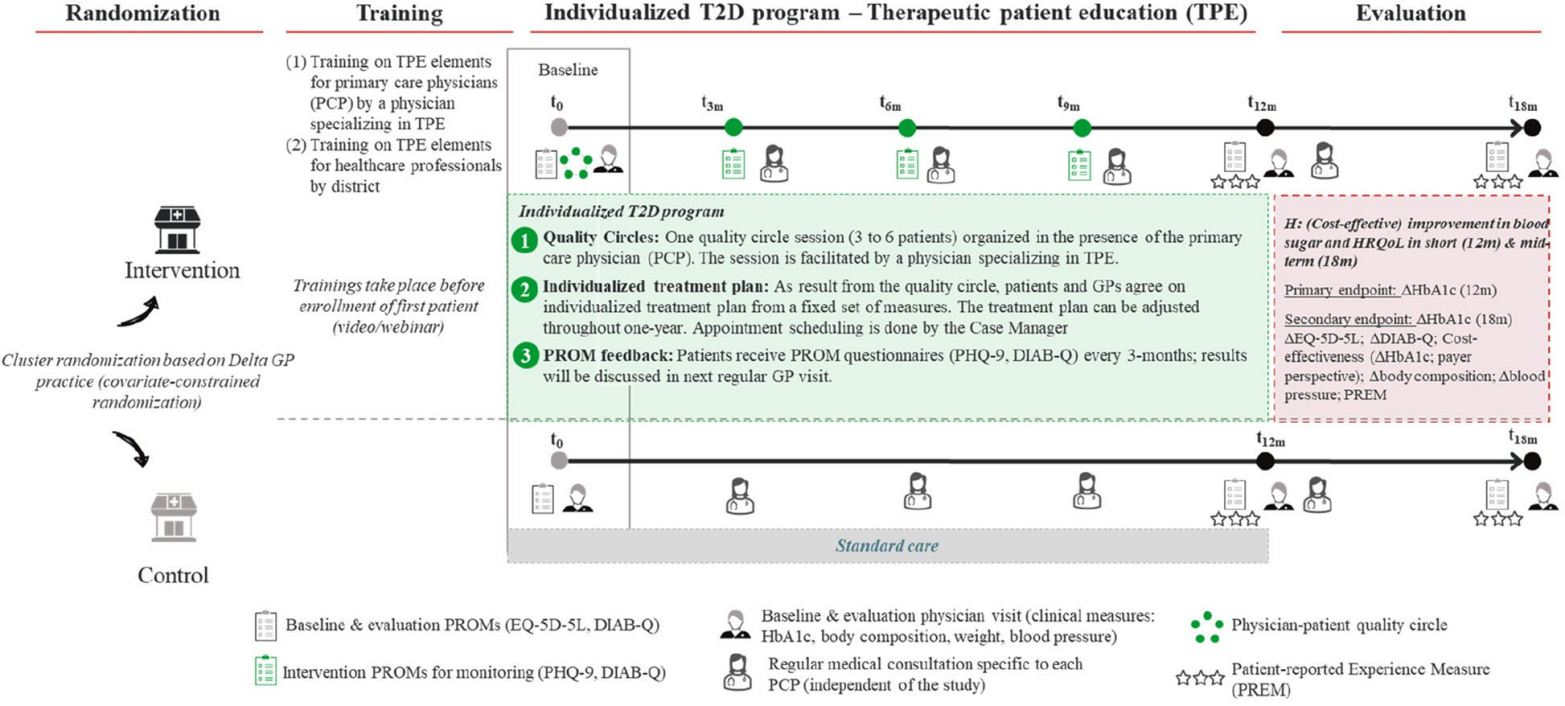
Trial design

The “Enhancing T2D Care” study is a two-arm multi-center cluster randomized controlled trial with a 1:1 patient allocation ratio and two parallel groups within a superiority framework [17]. Primary care physician (PCP) practices are randomized constrained for practice size, location, and specialization. As can be seen in Fig. 1, PCPs and associated healthcare professionals allocated to the intervention group (IG) will be trained on the TPE elements of the T2D program by professional educational patient therapists (TPE specialists) and on the utilization of PROMs within the program. Trainings will take place before enrollment of the first patient. The exact content of the training is described in the section Interventions.

Eligible patients in the IG will be treated within a 1-year T2D program, which centers around an initial (*t* = 0) physician–patient quality circle. This quality circle is facilitated by a TPE specialist. The PCP and the patient agree on an individualized treatment plan (e.g., individual or group session within healthcare specialist network) based on a needs assessment at each visit. Appointments are organized and scheduled by a dedicated study manager. Furthermore, the patient answers PROM questionnaires every 3 months and will discuss the results during the next regular PCP visit. Patients in the control group (CG) will follow standard care. PROMs used within the trial are the Patient Health Questionnaire (PHQ-9) [18] for psychological well-being and the Diabetes Intention, Attitude, and Behavior Questionnaire (DIAB-Q) [19] to detect any problems or distress with managing their diabetes. Primary endpoint is the mean change in HbA1c levels after 12 months, secondary endpoints are the mean change in HbA1c levels after 18 months, the mean change in HRQoL (∆ EQ-5D-5L), the mean change in intention to engage in diabetes self-care behaviors (∆ DIAB-Q), the mean change of blood pressure, the mean change of fat/lean mass (∆ body composition), and patient-reported experiences (PREMs) at 12 and 18 months. Claims data and intervention cost data will be used to evaluate cost-effectiveness with respect to mean change in HbA1c levels from the payer perspective.

## Methods: participants, interventions, and outcomes

### Study setting {9}

The trial is a multi-center CRT in the canton of Geneva in Western Switzerland, which includes T2D patients diagnosed within the last 10 years from around 30 PCP practices from Réseau Delta (risk and profit-sharing PCP network operating in various Swiss cantons). Within the IG, PCPs and their adjacent healthcare specialists are trained by TPE specialists and act, jointly with the study manager, as coordinating gatekeeper to tailor the patients’ treatment plan according to their specific needs and preferences within the T2D program.

### Eligibility criteria {10}

To be eligible for the study, patients must fulfill the following inclusion criteria:

1. T2D diagnosis within the last 10 years.
2. Age between 40 and 65 years at the start of the intervention.

Exclusion of patients is criteria are defined as follows:

1. Pregnant or breastfeeding women
2. Person not having the capacity to give informed consent as attested by a signature
3. Exclusion via significant comorbidities are at the discretion of the attending PCP. Only comorbidities associated with significant deterioration (preventing the candidate from participating physically in the quality circles) will be considered. The list of reportable comorbidities is based on the International Consortium for Health Outcomes Measurement (ICHOM) list of diabetes conditions and local clinical practice. These conditions comprise chronic obstructive pulmonary disease (COPD), peripheral vascular disease, dementia, hemiplegia, presence/history of anxiety disorders, depression, eating disorders, and psychotic mental disorders. The aim was to be as inclusive as possible in order to obtain generalizable (real world) results.

### Who will take informed consent? {26a}

Participating PCP practices from the physician network Réseau Delta will screen their patients for eligibility and approach them at their next visit. All patients will receive a study information and are required to sign an informed consent (see Appendix B). Study information looks differently between IG and CG to ensure blinding of participating patients. Open question can be discussed with the PCP, the PCP assistant or the dedicated study manager employed in this project. Participants are required to sign one or two forms: (1) participation in the study and the permission to use personal, medical and patient-reported data for evaluation, and (2) permission to merge claims data with aforementioned data for the evaluation of cost-effectiveness. The latter must only be signed by Groupe Mutuel insurees. The signed informed consent forms are stored electronically before initiating study procedures. Patients will be informed that, at any time, they can withdraw from the study. Their data will then be deleted and excluded from study evaluation.

### Additional consent provisions for collection and use of participant data and biological specimens {26b}

Not applicable—all information on consents forms is provided in {26a}.

## Interventions

### Explanation for the choice of comparators {6b}

The comparator selected in our study is standard care which corresponds to acquiring the right to practice in the canton of Geneve and generally follows the Swiss recommendations concerning type 2 diabetes [20] and the regional recommendations of the Centre for Primary Care Medicine of the University Hospitals of Geneva [21]. The treatment recommendations encompass four main elements: glycemic monitoring, prevention and management of complications and cardiovascular risk factors, therapeutic education and self-care, and lifestyle intervention. The reference HbA1c target is ~ 7%, individualized between 6.5 and 8.5% depending on comorbidities, disease duration, and hypoglycemia risk. T2D care is typically managed by PCPs, with specialist involvement when metabolic imbalance or complications occur. While this model ensures continuity, it remains fragmented, and access to structured patient education, nutritional counseling, and exercise programs is inconsistent. As T2D care in Switzerland is not managed in structured disease management programs with clear guidelines [15, 22], standard care in either form is taken as comparator. In addition to standard care, all PCP practices collect data required for the evaluation of the study at baseline (*t* = 0 m) and at the endpoints of the study (*t* = 12 m, *t* = 18 m), e.g., PROMs, HbA1c, and body composition.

### Intervention description {11a}

The intervention consists of two steps (see Fig. 1). First, PCPs and healthcare professionals of practices in the IG are trained in interprofessional group sessions on TPE elements by specialists (see Table 1). Second, patients of PCPs in the IG follow an individualized T2D program which is initiated in a quality circle with their PCP, other healthcare professionals, the dedicated study manager and other patients. The individualized T2D program is accompanied by regular PROM collections, which are discussed in the regular PCP visits.

**Table 1:** Design of interprofessional trainings on TPE elements for intervention group

Interprofessional trainings	
Objectives	• Physio-pathological refresher: Reinforce fundamental knowledge of the physiopathology of diabetes • Foundations of TPE: Review of the basic principles of therapeutic patient education • Review of current scientific literature: Update participants on the latest research and advances in the field of T2D management and care techniques • Creating a self-help network: Support promoting exchanges and sharing experiences between healthcare professionals • Exchange of experience: Discuss case studies and solutions applied to improve diabetes management • Use of a common language: Develop a shared vocabulary to ensure effective and coherent communication between the various parties involved in patient consultations
Recipients	Intervention group • PCPs • Other healthcare professionals (e.g., physiotherapists, dietician, psychotherapists, sports education specialists, acupuncturist, sophrologists, sports coaches)
Instructors	TPE specialists
Timing	Before inclusion of first patient
Frequency and duration	Once for 1 h 30 m
Methods	Training sessions are interactive, enabling dynamic discussions and exchanges of expertise between participants
Discussed TPE elements	• Disease management (complication management, self-monitoring, technical competencies, relieving symptoms, self-treatment) • Lifestyle changes (prevention of complications, implementation of lifestyle changes, awareness of risk factors) • Cognitive-behavioral skills (personal care, situational awareness, critical thinking, self-confidence, adaptability, problem solving) • Stress and sleep management (Information on disease processes, health-promoting behavior in the face of illness, etiology, treatment options) • Interpersonal skills (interpersonal skills, organizational information, psychosocial support incentives)

#### Step 1: Interprofessional trainings on TPE elements

Before the inclusion of the first patient into the study, PCPs and associated healthcare professionals of practices in the IG receive a joint training by TPE specialists (see Table 1 for details). The 1:30 h training will take place once for each group and will be interactive to enable dynamic discussions and an exchange of experiences between participants. The objectives range from a physio-pathological refresher on diabetes knowledge, over the foundations of TPE, to the creation of self-help networks between professionals, and the exchange of experiences as well as the use of a common language. Discussed TPE elements will be on (1) disease management, (2) lifestyle changes, (3) cognitive-behavioral skills, (4) stress and sleep management, and (5) interpersonal skills. Once the trainings have been conducted, the PCPs will start recruiting patients into the study.

#### Step 2: Quality circles, 1-year individualized T2D program for patients, and PRO measurements

The physician–patient quality circles (see Table 2) stand in the center of the intervention and present the starting point for the 1-year individualized T2D program that patients in the IG will follow. The quality circles are organized group sessions with a TPE specialist, the PCP, the study manager, and three to six patients (depending on practice size). Group sessions were chosen due to similar effectiveness compared to individual sessions [4] and their possibility of exchange between participants. Because the session is led by the TPE specialist, the PCP can take a different perspective and change his usual role from a directive to a response-solution approach. Within these sessions, the use of motivational interviewing techniques facilitates communication and patient involvement [23]. By jointly setting clear, motivating goals for patients to improve health and treatment adherence, the outcome of the quality circle is an individualized treatment plan for each patient according to their needs and preferences. The study manager ensures scheduling of activities and visits as well as medical follow-ups.

**Table 2:** Design of quality circle

Quality circles	
Objectives	• Change of physicians’ role: Encourage a response and solution approach rather than a directive one • Use of motivational interviewing techniques: Facilitate communication and patient involvement • Definition of learning objectives: Set clear, motivating goals for patients to improve adherence and clinical outcomes
Composition of the quality circle	3–6 patients (depending on practice size), TPE specialist (facilitator), study manager, PCP (recommended presence)
Roles of participants	TPE specialist: • Presentation of TPE elements and lead of quality circle Study manager: • Administrative and operational role (e.g., presentation of potential TPE activities, clinical and psychological follow-up, appointment management) PCP: • Observational role Patient: • Active participation
Timing	At baseline (*t* = 0)
Frequency and duration	1 h 30 m (minimum of one session)
Methods	Quality circles are interactive, enabling dynamic discussions and exchanges between professionals and patients, and among patients
Outcome	Definition of individualized treatment plan based on TPE elements for each patient according to their needs and preferences

The individualized treatment plan consists of one or several elements. It is essential that the patient commits voluntarily to the tailored treatment plan. Patients can choose from a number of activities and sessions that may or may not be covered by the Swiss LAMal health insurance scheme (Swiss Federal Law on Compulsory Health Care). Nevertheless, all services are free of charge for the patient.

◦ Services covered by the Swiss LAMal health insurance scheme: This includes individual sessions at the dietician, the physiotherapist, or the psychologist/psychotherapist.
◦ Services not covered by the Swiss LAMal health insurance scheme: This includes group sessions (e.g., group dietician session, discussion groups, classes with a professional in adapted physical activities); diaries and apps (e.g., dietary apps such as MySwissFoodPyramide, physical activity apps such as Asics RunKeeper, or apps for mental health such as Coherence cardiaque antistress); sport activities such as the rental of electronic bikes; and complementary medical services such as acupuncture, kinesiology, or sophrology.

The study manager organizes the patient’s corresponding treatment schedule, monitors adherence, and follows up with the patient. The individualized treatment plan can be adapted throughout the 12 months. Additionally, patients in the IG fill out PROM questionnaires at 3, 6, and 9 months (PHQ-9 [18] for measuring the patient-reported psychological well-being, DIAB-Q [19] to measure the patient-reported problems and distress with T2D). PROM results will be discussed in the following regular PCP visit to facilitate shared decision-making and enhance adherence to the treatment plan. The study manager plays a pivotal role in the intervention as he/she schedules and monitors the activities and sessions and is also the primary contact point for the patient. Furthermore, the study manager follows up after patients’ activities and ensures completion of PROM questionnaires.

### Criteria for discontinuing or modifying allocated interventions {11b}

At any time, patients can withdraw from the study. Their data will then be deleted and excluded from study evaluation. Patients will not be allowed to switch between intervention and control group.

### Strategies to improve adherence to interventions {11c} and plans to promote participant retention and complete follow-up {18b}

The dedicated study manager ensures that PCPs and patients adhere to the study protocol. At regular PCP visits, relevant data (see Appendix A) is collected, and it is monitored whether the PCP used the results from the PROM questionnaires during consultations. Regarding patients, appointments and activities are scheduled and followed-up on. The study manager also contacts patients when they miss appointments. To ensure high response rates of PROM questionnaires, the study software (SOKLE) sends out reminders to patients after 3, 7, and 14 days. Additionally, the study manager calls up patients when they missed to fill in PROM questionnaires and potentially assists the patients in filling out the questionnaires.

### Relevant concomitant care permitted or prohibited during the trial {11d}

All concomitant care is permitted. However, a rigorous and standardized outcome monitoring strategy has been put in place to ensure data validity and accurate evaluation of the intervention (see Appendix C).

### Provisions for post-trial care {30}

Not applicable, as post-trial care is not provided.

### Outcomes {12}

**Table:** 

Primary endpoint:
◦ Mean change in glycated haemoglobin (A1c) (∆HbA1c) after 12 months
Secondary endpoints:
◦ Mean change in glycated haemoglobin (A1c) (∆HbA1c) after 18 months ◦ Mean change in health-related quality of life (∆ EQ-5D-5L) after 12 and 18 months ◦ Mean change in intention to engage in diabetes self-care behaviors (∆ DIAB-Q) after 12 and 18 months ◦ Mean change of blood pressure (∆ blood pressure) after 12 and 18 months ◦ Mean change of fat/lean mass (∆ body composition) after 12 and 18 months ◦ Variation in the number of DDDs for antidiabetic drugs (ΔDDD ATC A10B) after 12 and 18 months ◦ Patient-reported experience measure (PREM) at 12 and 18 months ◦ Cost-effectiveness (∆HbA1c; costs evaluated from payer perspective) after 12 and 18 months

#### Primary evaluation criteria

Current recommendations indicate that the main objective of diabetes control is to keep HbA1c levels below predefined thresholds, while avoiding hypoglycemia. For most patients, this threshold will correspond to an HbA1c of 7.0%. In younger people with a short history of diabetes and/or patients with microvascular complications, the target may be reduced to 6.5% or lower, if it can be achieved without significant and repeated hypoglycemia. However, for older patients or in cases of comorbidity, a higher HbA1c target of 7–8% or lower is reasonable [24].

Recent reviews and meta-analyses that investigated the effectiveness of TPE interventions—whether at group level, at individual level, remote or in person—for T2D patients highlighted the frequent use of HbA1c levels as a primary outcome [4, 6, 25]. On average, significant reductions in HbA1c levels were observed in the IGs (e.g., standardized mean difference = 0.272; 95% confidence interval, 0.118 to 0.525; *n* = 7360 [25]).

In the context of this study, the HbA1c level will be monitored in the regular physician visits and the mean change of HbA1c level between baseline and month 12 will serve as the primary endpoint.

#### Secondary evaluation criteria

In terms of secondary evaluation criteria, we aim to measure seven different endpoints. First, we evaluate the mean change of HbA1c level between baseline and month 18 to observe whether the effects of the intervention lasted in the medium term. Second, HRQoL will be assessed. This measure provides patient-reported information on patients’ daily lives, beyond basic clinical health indicators [26]. The EQ-5D, a generic HRQoL measure, will be used in this study. This tool captures the multidimensional nature of the HRQoL, encompassing the dimensions of physical, mental, and social health [27]. It will be measured at baseline and at months 12 and 18 (mean change) and serve as secondary endpoint.

Third, in a diabetes specific PROM, the DIAB-Q will be used to measure intention to engage in diabetes self-care behaviors [19]. It is a brief (17 items) and psychometrically sound PROM. Besides the application of DIAB-Q to amplify patient-doctor communication in the IG, the mean change between baseline and months 12 and 18 will be used to evaluate if the intention, attitude, and behavior of enrolled patients towards their condition have changed due to the intervention.

Fourth and fifth, the mean change of two clinical measures, namely blood pressure and body composition, between baseline and months 12 and 18 will be used to evaluate the effects of the intervention on patients’ health status. Changes in body composition will be used to quantify changes in response to adapted nutritional habits or physical activity. The literature suggests quantifying body composition using bioelectrical impedance analysis. It has the advantage of being a non-invasive, mobile, portable and reproducible technique. It is based on measuring resistance and reactance in the body. According to the models generally used, body composition can be described in terms of two main compartments, fat mass and lean mass. Lean mass includes water, proteins, and minerals. Muscle mass and organs represent the metabolically active components of lean mass [28].

Sixth, in order to be able to assess changes in patients’ medication (medication intensity) on the basis of claims data, it will be possible to retrospectively assess the change in the number of DDD for anti-diabetic drugs (excl. insulin) according to the A10B anatomical, therapeutic, and chemical classification system (ATC Classification system). Only retrospective data on Groupe Mutuel patients will be available.

Seventh, we collect PREMs to assess patients’ care experience with a brief 9-item outpatient PREM questionnaire which was developed by the New South Wales (Australia) government and adapted to the Swiss context. The following dimensions are measured: satisfaction, subjective experience and objective experience, as well as relations with care providers. PREMs will be measured in months 12 and 18.

Finally, we asses cost-effectiveness of the intervention compared to standard care from payer (health insurance) perspective. The effect measure will be mean change in HbA1c. Cost information will be collected via Groupe Mutuel and participating health care facilities (e.g., intervention costs).

### Participant timeline {13}

Each patient will be participating in the study for 12 months; an additional follow-up measurement is planned after 18 months. Before enrolment of patients, practices are allocated to either the IG or the CG using covariate-constrained cluster randomization. Practices screen their existing patients for eligibility and contact them at their next regular visit. Patients in both groups sign the informed consent at enrolment. Baseline assessment in the IG will be right before their scheduled physician–patient quality circle and at the day of enrolment for patients in the CG. At baseline, participants provide casemix variables, fill in the baseline PROM questionnaires, and PCPs document patients’ medical data. During intervention, medical data, treatment adherence data, and responses to intervention PROMs are collected for patients in the IG. The intervention will take place in the 12 months after baseline and close out with an evaluation at month 12 (short-term) and an evaluation at month 18 (medium-term). Evaluation data collection consists of medical data, evaluation PROMs, and PREMs. Additionally, claims data from Groupe Mutuel are added for respective insurees. For more information on standard protocol items see Table 3.

**Table 3:**
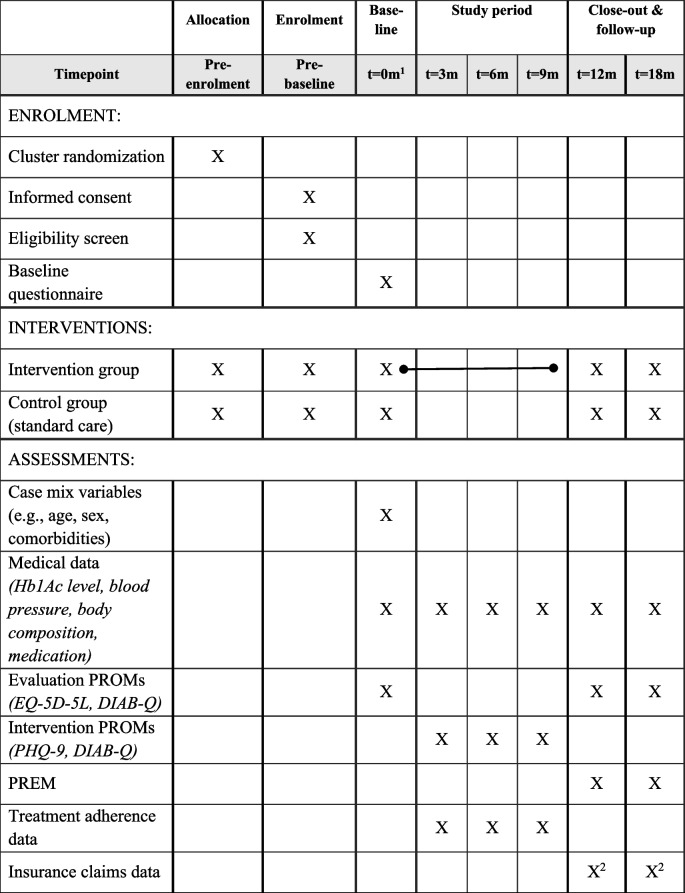
Standard protocol items

Annotations: (1) Baseline assessment for patients in IG before quality circle, for patients in CG at day of enrolment. (2) Only for Groupe Mutuel insurees. *DIAB-Q* Diabetes Intention, Attitude, and Behavior Questionnaire, *EQ-5D-5L* EuroQoL 5-dimension 5-level, *HbA1c* hemoglobin A1C, *PHQ-9* Patient Health Questionnaire, *PROM* patient-reported outcome measure, *PREM* patient-reported experience measure

### Sample size {14}

The required sample size was calculated based on the expected effect size with respect to our primary endpoint and the specificities of CRTs [29]. We assume an effect size of 0.58 percentage point difference in change of HbA1c between baseline and 12 months in the IG compared to the CG. This assumption is grounded on results from studies with similar intervention designs (e.g., TPEs in individual and group sessions) [4, 6]. Expected distribution (measured by standard deviation) of the primary outcome at baseline is 1.10 [4]; the intraclass correlation is assumed to be 0.032 [30]. From our sample of participating practices, we expect an average size of the cluster of five eligible patients with a coefficient of variation of 30%. We use an a-priori matching before randomization to balance the sample on previously known practice attributes (urban vs. rural location, practice size, specialization of PCP)—we assume a moderate group-level correlation between practice attributes and change in HbA1c [31]. Assuming a commonly used Type 1 error rate (alpha) of 5% and aiming for a power of 80%, the trial requires a minimum of 13 clusters of five patients in each cluster. The sample size was estimated using the sample size calculator of the National Institute of Health [32]. Anticipating a drop-out rate of patients until the end of the trial period of 15% [4], we need to recruit a minimum of 154 patients (77 in the intervention group, 77 in the control group).

### Recruitment {15}

Recruitment of the required practices will be out of the 130 Réseau Delta practices in the Swiss canton of Geneva. Patient recruitment will be carried out by PCPs in the participating practices. Concretely, PCPs will screen their patients for eligibility criteria and approach them at their next visit.

## Assignment of interventions: allocation

### Allocation (sequence generation {16a}, concealment mechanism {16b}, and implementation {16c})

Balanced cluster randomization will be performed by a statistician at the University of St. Gallen who will not be involved in the recruitment or training of practices. Randomization will be based on previously known practice attributes (location, practice size, specialization of PCPs). We use covariate constrained randomization [33] to minimize imbalance from practice attributes. To this end, we employ the cvcrand command in Stata 18, performing 1000 randomizations, and select the allocation that best balances the covariates based on principal component analysis. A clustered permutation test (cptest) is done to test the performance of the randomization.

Group assignments will be implemented through a pre-prepared pseudonymized list of practices provided by Réseau Delta to the University of St. Gallen. Practices are then randomly allocated. The allocation of each of the practices to either intervention or control is marked down in this list, which is then sent back to Réseau Delta who will implement the allocation. The allocation sequence will be securely stored and concealed. The investigator—Réseau Delta—only receives a finalized version of the practice allocation list. Practices will only be informed of their allocation before patient inclusion starts (see Fig. 1). Practices will enter the study arm to which they have been randomly allocated.

## Assignment of interventions: blinding

### Who will be blinded {17a}

This is a single-blinded study. Practices are aware of their allocation to IG or CG and are therefore not blinded. Patients in the IG and CG will receive different study information to prevent unblinding on the patient side.

### Procedure for unblinding if needed {17b}

There is no structured procedure for unblinding defined as we do not expect the necessity for unblinding.

## Data collection and management

### Plans for assessment and collection of outcomes {18a}

We collect three different data types to evaluate the primary and secondary outcomes:

1. Medical data: HbA1c level (via LumiraDx HbA1c [34]), body composition (via Biody Xpert ZM II [35], blood pressure (via Omron M3 [36] either at the PCP’s practice or at a suitable location chosen by the patient in consultation with the study manager; prior fasting is not required) and antidiabetic drug use (number of DDDs) are measured at baseline, 12 and 18 months at the participating practices and are documented by the PCP or the study manager in the study software SOKLE. In the IG group, these variables are also collected within the 12 months intervention during regular PCP visits.
2. Patient-reported data: PROMs (EQ-5D-5L, DIAB-Q, PHQ-9) and PREMs (Outpatient PREM questions—New South Wales government in Australia) are collected via the study software SOKLE. Patients receive PROM and PREM questionnaires via e-mail or SMS for self-administration. At baseline, manual completion (paper/pen) remains possible on request. The study manager is also available to help participants who encounter difficulties—however, the questionnaires are self-administered by the patients.
3. Claims data: After completion of the study, Réseau Delta identifies patients insured with the Groupe Mutuel, and requests claims data (treatment, medication, etc.) from Groupe Mutuel via the insurance number of these patients (cf. Data management {19}). This data is matched to the patient pseudonym and securely transferred to the University of St. Gallen.

### Data management {19}

Standardized case report forms are implemented within the study software SOKLE to prevent typing errors and irrational value entries. The software will be used by authorized PCPs at the participating practices and the dedicated study manager. Where interoperable interfaces exist, entered personal and medical data from the participating practices is directly transferred to the study software. Otherwise, the study manager will enter the relevant personal and medical data into the study software manually. Data interactions within the study software are closely monitored and audited, with access restricted to authorized individuals only.

For patient-reported data (PROMs and PREMs), patients use a unique key for questionnaire completion, enhancing data confidentiality and integrity. Data will be pseudonymized in the study software. For adding claims data, patients sign a consent form that authorizes sharing of social insurance numbers with Groupe Mutuel. This allows Groupe Mutuel to identify the claims data of the respective patients. The claims data is then sent back to the Réseau Delta, who merges the claims data with the respective pseudonym.

Réseau Delta transfers pseudonymized project data for evaluation to the University of St. Gallen by secure transfer (SFTP server). Before the data is transferred to the University of St. Gallen, all participants’ identifying information is deleted. Hence, the University of St. Gallen is not able to identify any participant. All analyses will be conducted on a pseudonymized data set. Published evaluation reports will be completely anonymous. All study data must be retained by the study sites and the University of St. Gallen for at least 10 years following the completion of the trial.

### Confidentiality {27}

Data is stored on a server located in Onex (Geneva, Switzerland), in a Réseau Delta data center—a closed data center that is only accessible to authorized persons. Only an administrator, who has signed the confidentiality charter for access to Réseau Delta data, has access to the physical server for maintenance and monitoring. The administrator must carry an identity card, and access is recorded. All data is backed up daily. Each month, previous backups are moved to an external disk. Backups are encrypted using a key stored in a vault (HashiCorp vault). All software is subject to an annual security audit by a third-party company. All elements of the audit report are brought into compliance within days of the audit. Protection mechanisms against major threats are in place, notably those listed in the TOP 10 of the Open Web Application Security Project. Sensitive information is encrypted in the database (passwords, identification keys, access tokens, etc.). The database is not accessible from the outside.

### Plans for collection, laboratory evaluation, and storage of biological specimens for genetic or molecular analysis in this trial/future use {33}

Not applicable, as no biological specimens for genetic or molecular analysis are used.

## Statistical methods

### Statistical methods for primary and secondary outcomes {20a}

We will apply a range of statistical methods to evaluate our CRT.

#### Primary endpoint

HbA1c will be analyzed with common descriptive statistics, i.e., sample mean, median, minimum, maximum, standard deviation, and interquartile range. We will follow the intention to treat principle, i.e., all included patients who did not withdraw consent will be analyzed. We will apply two-sample (independent samples) t-tests or Wilcoxon-Mann–Whitney tests (depending on distribution) for the primary endpoint for the mean differences at baseline, month 12 and month 18 and changes in mean difference from baseline to 12 and 18 months. As we anticipate cluster effects at the practice level due to the randomization method, our main model for the assessment of the primary endpoint will be a mixed-effects model. We will control for patient characteristics at baseline (e.g., baseline HbA1c, BMI, gender, age, comorbidities, diabetes duration) as fixed effects, which are likely to affect the primary endpoint. In addition, we will incorporate practice as a random intercept in the model to account for potential cluster effects at the practice level. We will not control for covariate-restricted randomization variables, as differences between practices are accounted for by random intercepts. We use the Wald Test or the Likelihood Ratio Test to evaluate hypotheses on fixed and random effects.

#### Secondary endpoints

Secondary endpoints (∆ EQ-5D-5L, ∆ DIAB-Q, ΔDDD ATC A10B, ∆ blood pressure, ∆ body composition, PREM) will be analyzed in a similar way taking into account different types of dependent variables and respective techniques, e.g., Wilcoxon-Mann–Whitney test for non-parametric data. Similar to the primary evaluation, the main model will be the mixed-effects model in which we will control for patient characteristics such as baseline outcome parameter value (e.g., EQ-5D-5L), BMI, gender, age and comorbidities as fixed effects, and incorporate clusters (practices) as random intercepts to also account for potential cluster effects. In addition, we will not control for randomization covariates. In analogue, we use the Wald Test or the Likelihood Ratio Test to evaluate hypotheses on fixed and random effects.

The cost-effectiveness analysis (CEA) will use ∆HbA1c as effect measure and costs evaluated from payer perspective including costs for all relevant treatments and drugs throughout the observed patient pathway at 12 and 18 months (see Appendix A, tab Groupe Mutuel). We further account for the cost of the intervention. The CEA will be performed in accordance with the consolidated standards for health economic evaluation reports (CHEERS 2022) [37]. The CEA will only be conducted for the participants that are insured at Groupe Mutuel due to data availability. In the case that the cost data are heavily right skewed, we will, as sensitivity analysis, perform a 95% winsorization on the cost data and repeat the CEA.

Analyses will be carried out using R, Stata, or Microsoft Excel as required.

### Interim analyses {21b}

No formal interim analyses are planned. However, we assess the adherence of both physicians and patients to the study protocol. This includes checking the consistency of data entries by physicians into the study software and monitoring patient participation rates in completing PROMs and adhering to intervention protocols. This analysis helps identify participation barriers and enables the implementation of strategies to enhance engagement and compliance.

### Methods for additional analyses (e.g., subgroup analyses) {20b}

Heterogeneity of treatment effect analysis will be applied to estimate treatment effects in clinically relevant subgroups and to identify which individual might benefit from the intervention. We will perform subgroup analyses for the following variables, as we hypothesize differential effects:

◦ Gender: male vs. female
◦ Age: below/above median age
◦ BMI: below/above 30 (obesity)
◦ A10B DDD: below/equal to/above the mean number of DDD on the same time base
◦ Baseline EQ-5D-5L score: below/above median baseline score
◦ Baseline score DIAB-Q: below/above the median baseline score

We will use a causal forest approach to estimate mean conditional treatment effects (see also [38]). We control for the same patient characteristics as in the mixed-effects model (e.g., baseline HbA1c, BMI, gender, age, comorbidities) but omit the subgroup variable as a control. In the event that the sample size is insufficient to use a causal forest approach, we will implement the mixed-effects model, including a subgroup dummy variable and an interaction term [39].

### Methods in analysis to handle protocol non-adherence and any statistical methods to handle missing data {20c}

The intention-to-treat principle will be followed. All included patients who have not withdrawn their consent will be analyzed. Multiple imputation techniques such as Random Forest Imputation (MissForest) will be used [40]. Drop-outs will not be considered in the final analysis. In the event of withdrawal, patients will not be replaced.

### Plans to give access to the full protocol, participant level-data, and statistical code {31c}

The protocol for our study will be made publicly available upon completion. Patient-level data will not be publicly available due to data privacy regulations. The statistical code used in our analysis will be available upon request.

## Oversight and monitoring

### Composition of the coordinating center and trial steering committee {5d}

The steering committee for the study consists of the principal investigator team from Réseau Delta, a responsible from the project sponsor Groupe Mutuel, the CEO of the study software SOKLE, and the academic director of the School of Medicine of the University of St. Gallen.

### Composition of the data monitoring committee, its role and reporting structure {21a}

Data and study adherence monitoring is done by the principal investigator (see Monitoring Plan in Appendix C). The study manager sends bi-weekly statistics on recruitment numbers, data entry quality, and PROM questionnaire response rates to the University of St. Gallen. The study manager in cooperation with the University of St. Gallen prepares bi-monthly reports to the steering committee to potentially initiate adaptations or other actions.

### Adverse event reporting and harms {22}

Although no adverse events or harms are expected to be caused by the intervention of this study, all adverse events and harms are documented and immediately (within a maximum of 24 h) reported to the steering committee. If it cannot be ruled out that an adverse event occurring in Switzerland is attributable to the intervention, the principal investigator reports it to the responsible Ethics Committee within 15 days.

### Frequency and plans for auditing trial conduct {23}

The detailed monitoring plan for the study (see Appendix C) implements a Risk-Based Monitoring strategy to ensure study quality and compliance, with centralized verification of source data, notably via extractable source data (according to time and date) from measuring devices (notably HbA1c levels). Remote opening, routine, and closing visits are scheduled as necessary to ensure protocol compliance, with direct access to data for the sponsor and ethics commission. Compliance with consent requirements and confidentiality of participants’ data are guaranteed, with additional checks planned in the event of non-compliance or problems detected.

### Plans for communicating important protocol amendments to relevant parties (e.g., trial participants, ethical committees) {25}

In substantial modifications to the study configuration and organization, the protocol and relevant study documents are subject to approval by the Ethics Committee prior to implementation. In an emergency, deviations from the protocol may be made to protect the rights, safety, and wellbeing of human subjects without prior approval from the Ethics Committee. Such deviations must be documented and reported to the Ethics Committee as soon as possible.

### Dissemination plans {31a}

Study results will be published in peer-reviewed journals. Publication will adhere to EQUATOR network guidelines [41]. All results will be presented in an aggregated, anonymized format, consistent with ethical standards for data reporting.

## Discussion

Current studies have shown the positive effects of different TPE elements on the reduction of HbA1c levels in several countries [4–6]. In this study, we want to test the (cost)-effectiveness of a T2D management program that combines regular PROM collection with TPE elements that are voluntarily chosen by patients within a trained network of healthcare professionals. This study not only aims at assessing effectiveness with respect to clinical outcomes (e.g., mean change in HbA1c levels, mean change in body composition) and PROMs (e.g., change in HRQoL) but also whether cost-effectiveness and patient experience allow for a wider roll-out in Switzerland.

While we designed the study in the best way possible, there are a couple of limitations to keep in mind. Firstly, most of the PCPs in the IG are using PROM questionnaires to monitor their patients’ health status and to better structure their visits for the first time. Though PCPs are trained on the relevance and the way of using PROM questionnaires for this study, the effect of using PROMs as monitoring tool and PROM results in regular visits might only fully uncover in the medium to long-term, when PCPs and patients get used to this approach. Secondly, differences of how practices in the CG define standard care might influence the differences in outcomes. In some cases, it might be possible that PCPs in the CG already use some form of TPE elements in their standard care protocol. In the statistical analyses, we will run models in which we also control for practice as random effects to isolate the effect of the intervention. Thirdly, as we only account for practice characteristics when cluster-randomizing, the patient characteristics between practices might differ considerably. Therefore, we estimate mixed effect models controlling for age, gender, and comorbidities. Lastly, it will hardly be possible to disentangle the effects from each element of the interdisciplinary and individualized T2D program. On the one hand, the T2D program is targeted to be as individualized as possible to create the highest impact for the patient. On the other hand, to be able to properly disentangle the impact of each element, the study would have been set up with multiple intervention arms. A study with a sufficient sample size to disentangle all possible effect combinations was financially and organizationally not feasible. However, the primary goal of this study is to estimate the effect of the entire program on health outcomes.

### Trial status

The current protocol is version 1, dated December 9, 2024. Recruitment of patients began on June 1, 2025, and will end around December 31, 2025. The study is expected to run until June, 2027.

## Supplementary Information

Additional file 1: Appendix A: Data variables

Additional file 2: Appendix B: Study information and informed consent forms

Additional file 3: Appendix C: Monitoring Plan.
Additional file 4: Appendix D: Ethics Committee approval

## Data Availability

Data sets generated and collected by this study will not be publicly available. Access to the collected data will only be granted to the University of St. Gallen and the participating Réseau Delta practices.

## Abbreviations

ATC classification: Anatomical therapeutic chemical classification
BMI: Body mass index
CEA: Cost-effectiveness analysis
CG: Control group
CRT: Cluster randomized controlled trial
DDD: Defined daily dose
DIAB-Q: Diabetes Intention, Attitude, and Behavior Questionnaire
EQ-5D-5L: EuroQoL 5-dimension 5-level
HbA1c: Glycated hemoglobin (A1c) HRQoL: Health-related quality of life
IG: Intervention group
PCP: Primary care physician
PHQ-9: Patient Health Questionnaire
PREM: Patient-reported experience measure
PROM: Patient-reported outcome measure
T2D: Type 2 diabetes
TPE: Therapeutic patient education
