# Supplementary material for: Enhancing type 2 diabetes care by an individualized and group-based therapeutic patient education program: study protocol for a cluster randomized trial
Source: Trials. 2025 Dec 23;26:577. doi: 10.1186/s13063-025-09302-x (PMC12723846; doi:10.1186/s13063-025-09302-x)
Supplement: Supplementary file 1 — Additional file 1: Appendix A: Data variables [file 13063_2025_9302_MOESM1_ESM.pdf]

Study code :	Randomization no :	Subject initials :

ALLIANCE DT2 CASE REPORT FORM

Improving care and quality of life for type 2 diabetes patients

Study reference number

**SITE:**	**Delta Care Network, Geneva**
**PRINCIPAL INVESTIGATOR :**	**Dr. Minette-Joëlle Zeukeng**

Subject initials :

Subject randomization number :

I am certain that the information provided in this form is complete and accurate. I confirm that the study was conducted in accordance with the protocol and any amendments to the protocol and that written informed consent was obtained prior to the study.

Signature of investigator: --

Date of signature :

D	d	m	m	m	y	y	y	y

This template has been provided free of charge by the Global Health Network. Please reference the Global Health Network when using it, and share your own documents in exchange. www.theglobalhealthnetwork.org.

NOTE : ***Coding of the participant taking part in the study***
Codes are generated automatically by SOKLE. The list of subject identification codes is stored on the SOKLE platform during the clinical study (restricted and secure access). In addition, after the end or interruption of the study, the list of subject identification codes will be kept confidential in accordance with Swiss legislation.

1. Eligibility criteria

**Inclusion criteria**		**Yes**	**No**
**1**	Is the participant between the ages of 40 and 65?	☐	☐
**2**	Has the participant been diagnosed with T2D in the last 10 years?	☐	☐
**3**	Has the subject given written informed consent? Date of informed consent (DD/MM/YYYY) :	☐	☐

**Exclusion criteria**		**Yes**	**No**
**1**	Does the participant have significant co-morbidities associated with worsening diabetes (such as renal failure, previous myocardial infarction, retinopathy, signs or symptoms of severe diabetic neuropathy or diabetic foot ulcers), severe osteoarthritis or is deemed unfit in the physician's judgment? treating?	☐	☐
**2**	Is the participant pregnant or breastfeeding?	☐	☐
**3**	Is the participant unable to give informed consent evidenced by a signature?	☐	☐

2. General participant information

- Identification code (ID) :
- AVS number :
- Randomization number :
- Age :
- Sex: M/F (0/1)

- Body composition:

3. Demographics

- **Level of education :**

  No training (1)/secondary school(2)/high school or university level(3)

- **Professional situation:**

  Employed (1)/Not employed (0):

- **Social status:**

  single (1), couple (2), family with children or parents (3)

4. Medical data

Please indicate if the participant has any of the following co-morbidities:

- Chronic obstructive pulmonary disease (COPD): Yes / No
- Peripheral vascular disease: Yes / No
- Dementia: Yes / No
- Hemiplegia: Yes / No
- Presence/history of anxiety disorders: Yes / No
- Depression: Yes / No
- Eating disorders: Yes / No
- Psychotic mental illness: Yes / No

5. Antidiabetic drugs (ATC code A10B)

Please ***register all*** *Diabetes type 2 medications*

Drug name	Dosage	Daily dosage

6. VISTES

6.1 Visit 1 (t0)

- Date:
- HbA1c :
- Blood pressure :
- Body composition :
- Questionnaires de reference:
  - EQ5D: yes/no
  - DIAB-Q: yes/no

6.2 Visit 2 (t3)

- Date:
- HbA1c :
- Blood pressure :
- Body composition :
- Questionnaires:
  - DIAB-Q: yes/no
  - PHQ-9: yes/no

6.3 Visit 3 (t6)

- Date:
- HbA1c :
- Blood pressure :
- Body composition :
- Questionnaires:
  - DIAB-Q: yes/no

- PHQ-9: yes/no

6.4 Visit 4 (tG)

- Date:
- HbA1c :
- Blood pressure :
- Body composition :
- Questionnaires:
  - DIAB-Q: yes/no
  - PHQ-9: yes/no

6.5 Visit 5 (t12)

- Date:
- HbA1c :
- Blood pressure :
- Body composition :
- Questionnaires:
  - EQ5D: yes/no
  - DIAB-Q: yes/no
  - PHQ-9 : yes/no
  - PREM (experience): yes/no
- Billing data (Groupe Mutuel): yes/no
- Number of medical visits with mpr in the last 12 months :
- Number of paramedical visits in the last 12 months :
- Type of group activity :
  - Dietetics: yes/no
  - Sports activities: yes/no
  - Discussions/information: yes/no

6.6 Visit 6 (t18)

- Date:
- HbA1c :
- Blood pressure :
- Body composition :
- Questionnaires:
  - EQ5D: yes/no
  - DIAB-Q: yes/no
  - PREM (experience): yes/no
- Billing data (Groupe Mutuel): yes/no

7. Collection of serious adverse events (in accordance with Oclin Art. 63)

Type (a, b, c or d)	Brief description	Degree of severity (minor, major, review)	Date	Duration	Link to causality with intervention	Brief description from measures companies	Document from reference eventual	Person from contact in the study

8. Closing/study exit

Date :

Reason for stopping studies (Check only the main reason. Reasons **other than study completion** require an explanation next to the answer)

☐ Study completed
☐ SAE **(fill in excel form if applicable)** ____________________
☐ Lost from sight ____________________ Non-
☐ compliant participant ____________________
☐ Medical contraindication ____________________
☐ Withdrawal of consent ____________________
☐ Death **(complete SAE form)** ____________________
☐ Other ____________________

Code d'étude :	Randomisation no :				Initiales du sujet :			

FORMULAIRE DE RAPPORT DE CAS
ALLIANCE DT2

Améliorer la prise en soins et la qualité de vie des patients diabétiques de type 2

Numéro de référence de l'étude

**SITE:**	**Réseau de soins Delta, Genève**
**INVESTIGATEUR PRINCIPAL :**	**Dre Minette-Joëlle Zeukeng**

Initiales du sujet :

Numéro de randomisation du sujet :

Je suis certain que les informations fournies dans ce formulaire sont complètes et exactes. Je confirme que l'étude a été menée conformément au protocole et à tout amendement au protocole et que le consentement éclairé écrit a été obtenu avant l'étude.

Signature de l'investigateur: --

Date de la signature :

D	d	m	m	m	y	y	y	y

Ce modèle de document a été fourni gratuitement par le Réseau mondial de la santé. Veuillez faire référence au Réseau mondial de la santé lorsque vous l'utilisez et partagez vos propres documents en échange. www.theglobalhealthnetwork.org.

NOTE : ***Codage du participant prenant part à l'étude***
Les codes sont générés de manière automatisée par SOKLE. La liste des codes d'identification des sujets est conservée à travers la plateforme SOKLE durant la réalisation de l'étude clinique (accès restreint et sécurisé). En outre, après la fin ou l'interruption de l'étude, la liste des codes d'identification des sujets sera conservée de façon confidentielle selon les conditions de la législation suisse.

Code d'étude :	Randomisation no :				Initiales du sujet :			

1. Critères d'éligibilité

**Critères d'inclusion**		**Oui**	**Non**
**1**	Le participant est-il âgé de 40 à 65 ans		
**2**	Le participant a-t-il reçu un diagnostic de DT2 dans les 10 dernières années		
**3**	Le sujet a-t-il donné son consentement éclairé par écrit ?		

Date du consentement éclairé (JJ/MM/AAAA) :

**Critères d'exclusion**		**Oui**	**Non**
**1**	Le participant, présente-t-il des comorbidités importantes liées à une péjoration du diabète (type insuffisance rénale, antécédent d'infarctus du myocarde, rétinopathie, signe ou symptôme de neuropathie diabétique grave ou d'ulcère du pied diabétique), arthrose sévère ou est jugé inapte selon le jugement du médecin traitant ?		
**2**	La participante est-elle enceinte ou allaitante ?		
**3**	La personne participante est-elle dans l'incapacité à donner un consentement éclairé attesté par une signature ?		

2. Informations générales du participant

- Code d'identification (ID) :
- Numéro AVS :
- Numéro de randomisation :
- Age :
- Sexe : M/F (0/1)

- Composition corporelle:

3. Données démographiques

- **Niveau d'éducation :**

  Sans formation (1)/école secondaire(2)/niveau haute école ou universitaire(3)

- **Situation professionnelle:**

  En emploi (1)/Sans emploi (0):

- **Situation sociale:**

  seul (1), en couple (2), en famille avec enfant/s ou parents (3)

4. Données médicales

Veuillez indiquer si le participant a les comorbidités suivantes:

- Maladie type bronchopneumopathie chronique obstructive (BPCO) : Oui / Non
- Maladie vasculaire périphérique: Oui / Non
- Démence: Oui / Non
- Hémiplégie: Oui / Non
- Présence/antécédents de troubles anxieux: Oui / Non
- Dépression: Oui / Non
- Troubles du comportement alimentaire: Oui / Non
- Maladies mentales psychotiques: Oui / Non

5. Médicaments antidiabétiques pris (code ATC A10B)

*Veuillez enregistrer **tous les** médicaments relatifs au Diabète de type 2*

Nom du médicament	Dosage	Dosage journalier

6. VISTES

6.1 Visite 1 (t0)

- Date:
- Taux HbA1c :
- Tension artérielle :
- Composition corporelle :
- Questionnaires de reference:
  - EQ5D : oui/non
  - DIAB-Q: oui/non

6.2 Visite 2 (t3)

- Date:
- Taux HbA1c :
- Tension artérielle :
- Composition corporelle :
- Questionnaires d'évaluation:
  - DIAB-Q: oui/non
  - PHQ-9: oui/non

6.3 Visite 3 (t6)

- Date:
- Taux HbA1c :
- Tension artérielle :
- Composition corporelle :
- Questionnaires d'évaluation:
  - DIAB-Q: oui/non

- PHQ-9: oui/non

6.4 Visite 4 (t9)

- Date:
- Taux HbA1c :
- Tension artérielle :
- Composition corporelle :
- Questionnaires d'évaluation:
  - DIAB-Q: oui/non
  - PHQ-9: oui/non

6.5 Visite 5 (t12)

- Date:
- Taux HbA1c :
- Tension artérielle :
- Composition corporelle :
- Questionnaires d'évaluation:
  - EQ5D : oui/non
  - DIAB-Q: oui/non
  - PHQ-9 : oui/non
  - PREM (expérience): oui/non
- Données de facturation (Groupe Mutuel) : oui/non
- Nombre de visites médicales avec mpr des 12 derniers mois :
- Nombre de visites paramédicales des 12 derniers mois :
- Type d'activité en groupe :
  - Diététique : oui/non
  - Mise en mouvement/activités sportive : oui/non
  - Discussions/information : oui/non

6.6 Visite 6 (t18)

- Date:
- Taux HbA1c :
- Tension artérielle :
- Composition corporelle :
- Questionnaires d'évaluation:
  - EQ5D : oui/non
  - DIAB-Q: oui/non
  - PREM (experience): oui/non
- Données de facturation (Groupe Mutuel) : oui/non

7. Recueil des évènements indésirables graves (selon Oclin Art. 63)

Type (a, b, c ou d)	Brève description	Degré de sévérité (mineur, majeur, critique)	Date	Durée	Lien de causalité avec l'intervention	Brève description des mesures entreprises	Document de référence éventuel	Personne de contact dans l'étude

8. Clôture/sortie d'étude

Date :

Raison de l'arrêt des études (Ne cochez que la raison principale. Les raisons **autres que l'achèvement de l'étude** nécessitent une explication à côté de la réponse)

- ☐ Étude achevée
- ☐ SAE **(remplir le formulaire excel le cas échéant)** ____________________
- ☐ Perdu de vue ____________________
- ☐ Participant non conforme ____________________
- ☐ Contre-indication médicale ____________________
- ☐ Retrait du consentement ____________________
- ☐ Décès **(remplir le formulaire SAE)** ____________________
- ☐ Autres ____________________
