# Supplementary material for: Enhancing type 2 diabetes care by an individualized and group-based therapeutic patient education program: study protocol for a cluster randomized trial
Source: Trials. 2025 Dec 23;26:577. doi: 10.1186/s13063-025-09302-x (PMC12723846; doi:10.1186/s13063-025-09302-x)
Supplement: Supplementary file 2 — Additional file 2: Appendix B: Study information and informed consent forms [file 13063_2025_9302_MOESM2_ESM.pdf]

RESEAU DELTA
LA SANTE INTELLIGENTE

Geneva study to improve care and quality of life for diabetic patients with diabetes. type 2 (Alliance DT2) in the outpatient setting*: Summary intervention information sheet* *(V3.0 11.10.2024)*

Dear Sir/Madam,

On the recommendation of your Delta Network physician, we invite you to participate in the study "Improving care and quality of life for type 2 diabetes patients (Alliance DT2)". Your Participation is entirely voluntary. All data collected as part of this study are subject to the following conditions strict data protection rules.

This study was initiated by Delta Network, the study promoter. The latter assumes responsibility management and financing of the study. The scientific part is carried out in partnership with the University of St. Gallen.
Gall and the technology partner is SOKLE, a company specializing in secure data management. medical care. Groupe Mutuel, the health insurer, is providing financial support for this project. study. The latter provides the data for the medico-economic analysis.

Réseau Delta, Ms Cecilia Rios Valente, Health Manager, 59C route de Chancy CH-1213 Petit Lancy
079 557 88 59 (8:30 a.m. to 6 p.m., Monday to Friday) cecilia.rios-valente@reseau-delta.ch

Here are the key points. You can find more detailed information by scanning the QR code.

Why are we conducting this study?

- The number of people with type 2 diabetes continues to rise worldwide. For chronic diseases such as diabetes, therapeutic patient education is on the rise. for over 30 years. Using this method, we were able to observe a decline or even a cure for type 2 diabetes (T2DM) in several international scientific studies.
- In Switzerland, in the outpatient setting, no study has yet been carried out to prove the importance of this type of treatment. of therapeutic patient education for optimal care of T2DM, coupled with specific questionnaires to assess quality of life in relation to the patient's health situation.
- This study gives patients the opportunity to express their views on their life with the disease, and to choose the activities/tools that seem important to him/her to change his/her lifestyle habits in order to to manage diabetes in collaboration with healthcare professionals (therapeutic alliance).

What should I do if I agree to take part?

- We'll ask you to fill in health-related questionnaires every 3 months for a year, with the help, if necessary, of the health manager, over 18 months.

→ *Health-related quality-of-life questionnaires: general EQ5D (5 questions),related to depression/anxiety PHQ-9 (9 questions) and diabetes-related DIAB-Q (17 questions)*

→ *Questionnaire related to experience in the healthcare system - (9 questions) at the end (12 months) then 6 months later*

- You will be contacted by the health care manager or other health care professionals to assess your rate. glycated hemoglobin and blood pressure as well as your body composition will be assessed at the beginning and end of the operation (12 months) and 6 months afterwards (total period: 18 months).
- If you decide to participate, you will be part of the group benefiting from the intervention. In the group of intervention, you will be treated with the care method dispensed by several health professionals (dietician, physiotherapist or sports coach) to implement movement, etc.) and a variety of approaches, including therapeutic patient education.

What are the benefits and risks of participating in the study?

PROFITS

- Personalize and optimize your care of T2DM by the healthcare professionals around you. There is no additional cost for you to participate in the study program. Some activities on offer not covered by your health insurance are financed by the Delta Network.
- Through your participation, you contribute to gathering essential information for evaluating and improving our products and services. quality of care for type 2 diabetes in family practices.

- You'll receive a welcome pack of "essential" accessories (water bottle, elastic bands, apron).

RISKS & CONSTRAINTS

- By participating in the study, we do not expose you to any risks or constraints.

Application to participate in medical research

INTERVENTION Detailed information

Improve the intake in care andthe quality of of life of patients diabétiques de type 2 (DT2) / Value-based care in type 2 diabetes

Simplified title : *Alliance DT2 / Enhancing T2D Care*

Dear Sir/Madam,

We'd like to introduce you to our study and invite you to take part. Before a new method can be disseminated and offered to physicians and other healthcare professionals. Research is needed to find out how this method works.

In medicine, such research is called a **clinical study**. In our study, we want to find out What method of care is provided by several health professionals (doctors...)? nurses, dieticians, specialists in adapted physical activity...) and according to several approaches including therapeutic patient education has on your daily life in your type 2 diabetes requiring treatment. That's why we're asking you if you'd like to participate in this study.

Your participation is voluntary. This **detailed information form** is designed to help you make your decision**.** decision. You can ask any questions you may have during a **consultation with your GP or the people in charge of the study, known as investigators or health managers**. These are responsible for the follow-up of study participants. If you wish to Please sign the **declaration of consent** at the end of this document. By With your signature, you confirm that you have read and understood the information provided. If you do not understand If you have any questions, please do not hesitate to ask the investigators or your doctor for clarification.

The information and consent form consists of three parts:

Part 1The essentials in a nutshell
Part 2Detailed information on the study
Part 3Data protection and insurance coverage

Part 1 gives you an overview of the study. In **Part 2**, we explain in detail the course and context of the study. **Part 3** contains information on the protection of and insurance coverage. By signing the consent form, you certify that you have understood the following and that you agree to participate.

This study is initiated by the Delta Network. This institution is called the promoter. The promoter assumes responsibility, management and financing of a study. The scientific part is carried out in partnership with the University of St. Gallen, technology partner SOKLE, a specialist in the secure management of medical data. Financial support is provided by the health insurer Groupe Mutual for this study.

The contact person for this study is:

Ms. Cecilia Rios Valente, Health Manager, Delta Network

Address 59C route de Chancy, CH - 1213 Petit Lancy
Phone 079 557 88 59 (8:30 a.m. to 6 p.m., Monday to Friday)
Email cecilia.rios-valente@reseau-delta.ch

Part 1: Quick facts

1. Why are we conducting this study?

If you've been living with type 2 diabetes for less than 10 years, we'd like to ask you if you'd like to know whether you wish to participate in this study.

For type 2 diabetes, the standard treatment is to follow the management plan proposed by your healthcare professional. physician, in order to avoid complications such as myocardial infarction, a stroke or a heart attack. cerebrovascular, eye, kidney or nerve damage. No improvement in results measured by blood tests, long-term use of insulin injections, etc. would then become necessary.

In this study, we examine how a new approach to the care of type 2 diabetes can be used to improve the quality of life of patients with type 2 diabetes. 2 in his daily life, provided by several health professionals (doctors, nurses and nurses, dieticians, specialists in adapted physical activity, etc.) and according to several approaches including therapeutic patient education. We will evaluate its effectiveness in the disease, quality of life and also from an economic point of view. This type of care would optimize your treatment to move towards a cure. Diabetes remission from type 2 is possible.

Find out more about the scientific background to the study **in Chapter 4**.

2. What do you have to do if you take part?

Your participation will last 18 months. A follow-up will be carried out every 3 months for one year, then every 6 months. A doctor-patient Quality Circle (QC) group session will be organized with your doctor. physician. A half-day medical certificate may be issued in order to participate in the session. CQ. As part of the study, you will be called upon by our health manager to take certain decisions. clinical measurements and complete short questionnaires. At the same time, you will continue your treatments standards (consultation) with your GP.

The average appointment with our health manager lasts less than 20 minutes. Appointments are detailed in **the figure in Chapter 5**.

If you decide to participate, your care remains the same as it is now with in addition to the possibility of choosing optional activities such as exercise, dietetics, etc. or related to mental health (including services not covered by basic insurance).

Find out more about the study process and procedures in **Chapter 5**.

3. What are the benefits and risks of participation?

Benefits

The aim of this study is to improve the management of type 2 diabetes and move towards a cure. It It should be stressed that international scientific studies have already shown a direct benefit on patients like you (improved laboratory values, increased physical activity...), weight loss, curing diabetes, etc.). In all cases, your participation can help to future patients. However, you may not be cured of your diabetes as a result of your participation in this study, but you will be contributing to the advancement of knowledge and science.

Risks

This method of care is new to the outpatient setting and has not yet been tested in Switzerland. However, the risks are minimal, if not non-existent, given that you continue to be and that the study will not interfere with your medication. (unless it becomes unnecessary or the dosage needs to be lowered because your laboratory data has improved).

Adverse reactions may occur if you choose not to follow your doctor's instructions. physician or other responsible healthcare professional. This will not be directly related to the present study.

We may not yet know all the risks and undesirable effects of the method. However, to date, no adverse effects have been reported.

Further information on possible constraints can be found in **chapter 6**.

Part 2: Detailed information on the study

4. Scientific background

4.1 Background: why are we conducting this study?

Type 2 diabetes (T2D) is a common chronic disease. When you have T2DM, if none of your changes in lifestyle habits, such as eating habits or activity levels is undertaken, the disease progressively worsens and sometimes requires insulin to be taken subcutaneously. as a long-term daily injection. The treatment and management of diabetes therefore depend on a The company has to take into account a multitude of parameters, including its own lifestyle.

Diabetes is generally treated with medication and possibly sessions with a diabetes specialist. someone specializing in dietetics and/or physiotherapy.

A great deal of research has already been carried out into the management of type 2 diabetes. Visit studies carried out to date, mainly in hospitals in Switzerland, have shown that interdisciplinary management, i.e. in collaboration with healthcare professionals of various specialties, along with therapeutic patient education, is necessary for effective management. of a diabetic patient. In fact, diabetes must be seen as a chronic disorder of the body. and complex way in which our body manages sugar, and for which a patient can with appropriate help (therapeutic alliance) restore lost equilibrium, once diagnosis is established early enough. So we know that, under certain conditions, it is possible to recover from type 2 diabetes.

In this study, we therefore examine whether interdisciplinary care accompanied by education to the patient is effective in improving clinical parameters such as hemoglobin and body composition (fat, muscle and bone content), as well as the quality of life associated with health. And finally, to be able to observe the economic impact of this care with a view to accessible to all.

4. 2 Structure of the study: how do we proceed?

In our study, participants were randomly assigned according to their treating physicians (assignment This is known as randomization. This method is important for reliable results. Each group receives a different treatment.

4. 3 Regulation of scientific research involving human beings huma ins

We are carrying out this study in accordance with the laws in force in Switzerland (law relating to research on human subjects). (e.g. human rights, data protection laws). In addition, we comply with all applicable internationally recognized standards. The study was reviewed and approved by the relevant ethics commission.

Our study is carried out in the Canton of Geneva. There are several hundred participants affiliated to the Réseau Delta in Geneva.

You will also find a description of this study on the website of the Swiss Federal Office of Public Health. public, at www.kofam.ch, under SNCTP registration number XXX or BASEC number XXX.

5. Course of the study

5. 1 What do you have to do if you take part in the study?

Participation in the study is voluntary and lasts 18 months. You must adhere to the appointment schedule. you → chapter 5.2) as well as all instructions given by the research team and your doctor processing.

You must inform the research team or your treating physician directly.

- if your state of health changes, for example if you are feeling less well or if you new disorders; you must continue to inform him if you withdraw of the study → chapters 5.3 and 5.4);
- For women: if you are planning a pregnancy or are pregnant (risk of gestational diabetes)

5. 2 What happens during appointments?

An initial consultation Patient-physician quality circles including your primary care physician and health management is mandatory.

Appointments are part of your standard diabetes management. In addition to your medical appointments, the Health Manager (GS) can call on you for medical appointments. for clinical measurements and questionnaires.

As part of the study, you will be asked to complete questionnaires every 3 months for the following periods one year and then at 6 months, on site or electronically (by telephone if necessary) with our health manager, according to your personal choice. Some clinical measures (such as glycated haemoglobin, blood pressure, body composition) will be taken in the office by the GS or delegated to other healthcare professionals. Additional appointments may be scheduled arranged with a dietician, physiotherapist, adapted physical activity professional, etc, psychotherapist as required, based on your individualized T2D program. Group activities can also be arranged at your request. All activities will be covered by your basic insurance or, depending on the activity, in particular the one in by the Delta Network. The Delta Network will assume responsibility for its share of the activities taken over by the group. insurance costs initiated as part of the study. An appointment can last between 20 minutes (simple consultation) and up to 2 hours (group dietetics course, for example). The list of intervention steps is shown in the figure below.

Here's what we do at every appointment (with your primary care physician, an educational specialist, etc.) or health manager):

- We answer your questions.
- We ask you questions about your health and quality of life.
- We measure routine clinical parameters
- We can help you fill in questionnaires or make them available to you.

During certain appointments, we also :

- Obtaining a drop of blood from the fingertip for glycated hemoglobin testing
- Blood pressure measurement
- Measuring body composition and body mass index
- Health-related quality of life questionnaire :
  - Generic quality-of-life questionnaire EQ5D (5 questions)
  - PHQ-9 depression anxiety questionnaire (9 questions)
  - Diabetes-specific questionnaire DIAB-Q (17 questions)
- Questionnaires related to experience in the healthcare system
  PREM questionnaire - satisfaction survey (9 questions)

These examinations/questionnaires enable us to assess the effectiveness of the intervention method.

Questionnaires are in electronic format (preferred). Paper format is available on request. available.

To sum up, your participation in the study is structured as follows (Figure 1):

1. Organization of a doctor-patient Quality Circle (QC) (group session) during which discuss the various aspects of type 2 diabetes with your doctor and a physician specialist in therapeutic patient education for the first part (approx. 1h30). During The second part (approx. 0.30 hrs). The health manager presents the different activities options to choose from. With the latter, you can determine an individual DT2 program according to your wishes.
2. Directly following the QC, the health manager takes the various clinical measures (glycated haemoglobin, body composition and blood pressure) and submits the first set of questionnaires (EQ5D and DIAB-Q) that you can fill out on site or at the home.
3. Three months after the QC, you will be asked to complete the short questionnaires again. (DIAB-Q and PHQ-9). This is also done at 6 and 9 months. At the same time, you follow activities/services you have chosen, and you visit your doctor at the frequency usual. During consultations, you can discuss the following with your doctor questionnaires. The latter will be able to consult them via the SOKLE platform.
4. One year (12 months) after the QC, you are again invited by the health manager to fill out a questionnaire. the evaluation questionnaires (EQ5D and DIAB-Q) as well as the one relating to your experience, called PREM (satisfaction). You are also called in for clinical measurements. At parallel you continue to follow the optional activities you have chosen.
5. Six months later, you are again asked by the health manager to fill in the form. evaluation questionnaire (EQ5D and DIAB-Q) as well as the one relating to your experience, named PREM (satisfaction). You will also be called in for clinical measurements.

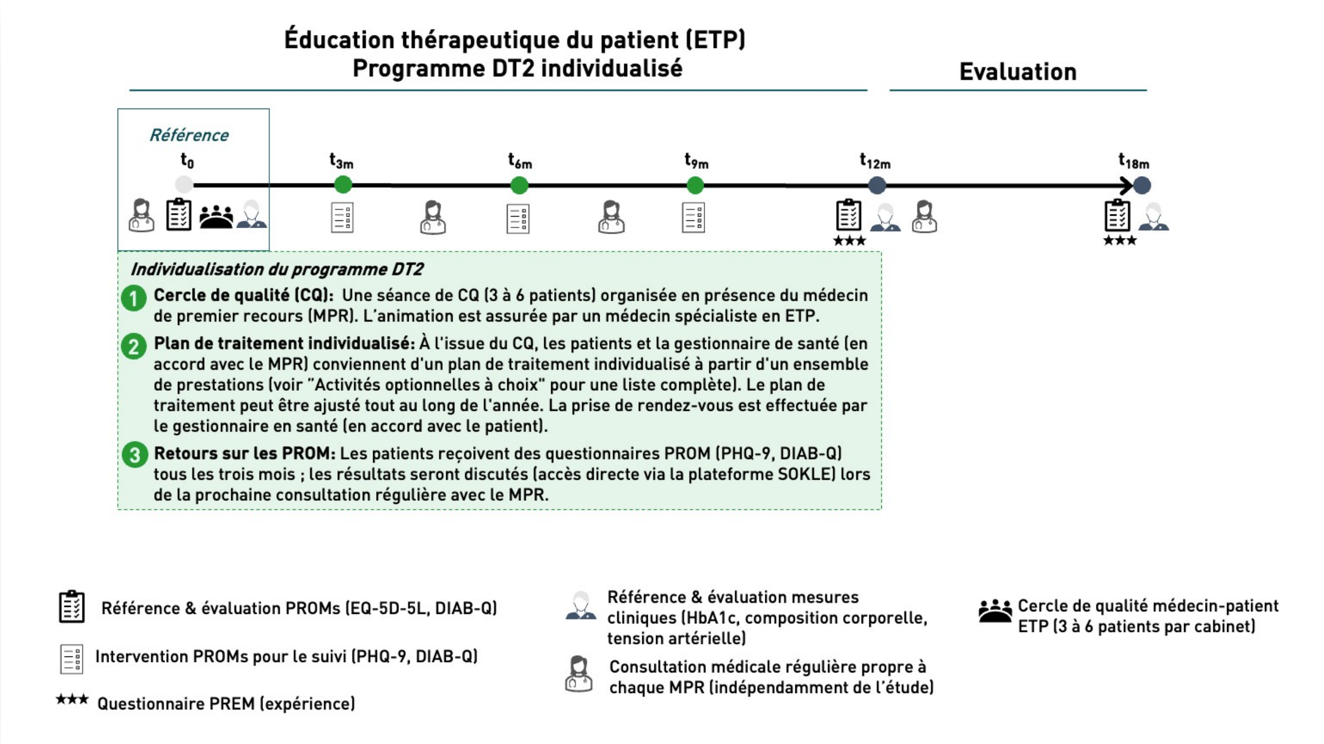

Figure 1 Study procedure for the "intervention" patient group

You will receive a precise summary of study-related appointments via the health manager. Please note that, in principle, it is not possible to reschedule an appointment. Should you nevertheless If you need to reschedule an appointment for important reasons, please inform us as soon as possible.

5. 3 What data are processed as part of the study?

For the purposes of this study, you authorize Delta Network via SOKLE to share your insurance number with the Delta Network. coded with your health insurance. For Groupe Mutuel policyholders, this allows Groupe Mutuel to identify the billing data of the respective participants. Billing data (in particular linked to your medication) are then sent back to the Delta Network, which associates them with the corresponding code. through SOKLE for study analyses.

5. 4 When does participation in the study end?

Your participation lasts 18 months (after completion of final questionnaires and measurements). clinical). You can interrupt your participation at any time before this date → chapter 5.4). You do not need to justify your participation. If you wish to terminate your participation, please contact us. inform the health manager, who will report it to the principal investigator.

If you withdraw from the study, your medical care and treatment will not be affected.

→ chapter 5.4 for other treatment options).

If you withdraw from the study, we can still analyze the data. Your study data remains coded → chapter 9).

5. 5 What happens if you don't want to participate?

If you do not take part in this study, your treatment and medical care will be assured. in line with current standards.

5. 6 Pregnancy

Pregnancy causes physiological changes that disrupt your clinical test results (glycated haemoglobin, body composition, etc.). This means you can't have children. during your participation in the study. This applies to all persons involved in this study. study. You can discuss these questions with your doctor or the investigator. contact.

For people who want to start a pregnancy

You should avoid becoming pregnant while taking part in the study (risk of diabetes). gestational). You must inform your partner(s) that you are taking part in this study. If you are breast-feeding or pregnant, you will not be able to take part in the study.

6. risks, constraints and undesirable effects

6. 1 What are the risks and constraints associated with the study?

Participating in this study does not entail any risk, but there are direct and indirect constraints. only, like any medical treatment of an illness.

If you are currently taking medication, please refer to your doctor's instructions. doctor or pharmacist. The official drug information (package leaflet) is directly accessible on the following website: https://www.swissmedicinfo.ch/?Lang=FR .

6. 2 Risks and constraints associated with examinations in the context of the study

We carry out various medical examinations for this study → chapter 5.2). These examinations are proven procedures for standard treatment by your GP. Nevertheless, they may involve risks and constraints, i.e. they may be unpleasant or have a negative impact on the environment. adverse effects. In this study, the risk/constraint is as follows:

- Fingertip prick/blood sampling: hematomas (when blood is drawn), bruising (when a blood sample is taken). bleeding or swelling may occur at the puncture site.

7. Financing and compensation

This study was initiated by Réseau Delta and is financially supported by Groupe Mutuel. insurance. The University of St. Gallen is the independent research institute commissioned to carry out the analyses. (scientific partner). The technological partner is SOKLE, an IT company based in Geneva. specialized in medical software. For its policyholders, Groupe Mutuel makes available for study billing data in coded form. For policyholders of other funds, access to data is carried out according to contractual possibilities.

There is no financial benefit to the researchers involved in the study.

If you take part in this study, you won't receive any money directly, but compensation (pack the essentials - water bottle - apron - elastic band", course offers / services not taken into account basic insurance, reimbursement of the portion of benefits directly related to study).

Participation in the study entails no additional costs for you or your insurance company. health insurance. Some of the activities offered are not covered by basic health insurance. funded by the Delta Network.

8. Study results

The results that concern you personally are communicated to you by your attending physician or the health manager.

In addition to individual results, the study will produce global results derived from data from all those involved. This would involve, for example, new knowledge about the factors/activities important for curing type 2 diabetes → chapter 4.1). These results do not apply to you necessarily directly. If you wish, on request, the investigators will provide you with a summary of overall results at the end of the study.

Part 3: Data protection and insurance coverage

9. Data and sample protection

We protect your data (e.g. medical data such as glycated hemoglobin, blood pressure and body composition). Swiss law lays down strict rules governing data protection.

Swiss data protection legislation gives you the right to access, rectify and receive information about your personal data. data collected, processed and transmitted as part of the study. In some cases due to other legal or regulatory requirements, these rights cannot always be exercised. be guaranteed. If you have any questions on this subject, please contact the Network Health Manager Delta for more information.

9. 1 Data and sample coding

All studies generate data from examinations (e.g. glycated hemoglobin, blood pressure, etc.). and body composition). These data are recorded in coded, electronic form. Encryption means that personal information that can directly identify you is stored *separately* from other data, in the form of a list (identification list) that identifies each person with a unique code. This means that your name, date of birth or address *cannot be used. do not appear* directly with the other data collected. This identification list remains for 10 years with the Delta Network and is then completely anonymized (access code required). specific user). No one else receives it. Special exceptions are covered in the 9.4.

When we transmit data for this study to the research institute, they are and your personal data is protected. The same applies when the list of policyholders is transmitted to your partner health insurer for billing data matching. Delta network (coding).

9. 2 Data and sample security during the study

The promoter Réseau Delta and the company SOKLE are responsible for the security of your data. of this study. They ensure compliance with current legislation, such as laws on the protection of intellectual property. data. Here is how the study sponsor contractually protects your data:

- -Delta & SOKLE Network Convention
- -Delta Network & Groupe Mutuel agreement and appendix
- -SOKLE & Groupe Mutuel agreement
- -Agreement University of St. Gallen & Groupe Mutuel
- -Delta Network & University of St. Gallen data transfer agreement

In this study, your data will be entered and transmitted electronically. The data is stored on a server located in Onex (Geneva, Switzerland) in a Delta Network data center. Only an administrator, who has signed the confidentiality charter for data access, has access to the physical server for maintenance and monitoring. The administrator must be in possession of a and access is recorded. All data is saved daily. After one month, previous backups are moved to an external disk. However, the risk of access to your personal data by unauthorized persons cannot be entirely ruled out (e.g. risk of "hacking").

It may be important for your treating physician to share anonymized medical data with the research team and coded data with your partner health insurer, with your authorization. This also applies to any other doctors who may be treating you. By signing the consent, you authorize the communication of this data, if necessary.

9. 3 Data security after the end of the study

When the study is over, the sponsor continues to ensure the security of your data. The law prescribes that all study documents, e.g. data collection forms, should be kept for at least 10 years.

At the end of this long period, the study data remain coded (or, where appropriate, are coded). fully anonymized for the research institute). Health-related data in your file including those in this study, are and always will be available to your caregivers. To to access to data, it is required to a access access. This excludes use/consultation by third parties.

Once the study is completed, the results are generally published in scientific journals. For this purpose, data is sent in anonymized form to other specialists if necessary. so that they can revise the publication. These data can be reused for analysis purposes. or other future research questions, but cannot be reused for any other purpose. by third parties.

9. 4 Consultation rights during inspections

The conduct of this study may be monitored by the relevant ethics commission. Visit sponsor must also carry out checks to guarantee the quality of the study and its results. For these checks, a small number of specially trained people have access to your

personal data and your medical file. In this case, the data is *not* coded. Anyone consulting your unencrypted data is bound by professional secrecy.

As a participant, you have the right to consult your data at any time.

10. Insurance coverage

You are covered by insurance if you suffer any damage as a result of the study - i.e. The procedure is regulated by law. The procedure is regulated by law. If you feel you have been you have suffered any harm as a result of the study, please contact your doctor or the local hospital directly. health manager or your private insurance company.

If damage results from the correct use of a conventional treatment, the rules of the law apply. are the same as for treatment outside a study. In such In such cases, liability insurance covers the costs.

Consent declaration for participation in the "Improving the quality of life" study care and quality of life for type 2 diabetes patients (Alliance DT2)" / *Value-based care in type 2 diabetes (Enhancing T2D Care)".*

Please read this form carefully. Do not hesitate to ask us questions if you do not understand. or if you require further information. Your written consent is required for participate.

**BASEC number**	
**Study title**	*Value-based care in type 2* diabetes / Améliorer la prise en soins et la qualité de vie des patients diabétiques de type 2
**Simplified title**	Alliance DT2 / *Enhancing T2D Care*
**Institution responsible**<br>(promoter and address)	Delta SA care network<br>Dr Philippe Schaller, MD, FMH, MPH<br>98 route de Chancy<br>CH - 1213 Onex
**Location**	Canton of Geneva
**Principal Investigator**<br>**Investigator-Coordinator**<br>**Health Manager**	Dr. Minette-Joëlle Zeukeng, PharmD, FPH, PhD<br>Ms. Joëlle Coclet, PhD<br>Ms Cecilia Rios Valente
**Participant**<br>Print name and surname: Date of birth :	

Attestation by attending physician/health care manager: I hereby certify that I have explained to the participating in the nature, size and scope of the study. I hereby declare that I have fulfilled all in relation to this study under Swiss law. If I were to become aware, during the course of the study, likely to influence the participant's willingness to take part in the study, I undertake to inform him immediately.

Place,<br>date	Full name of **attending physician/health manager**<br>in block letters<br><br>Signature

RESEAU DELTA
LA SANTE INTELLIGENTE

- I have received oral and written information about the study (purpose, progress, benefits & risks) from From my attending physician/investigator/coordinator/health manager undersigned.

- I am voluntarily participating in the study.

- I have had sufficient time to make my decision. I keep written information and I receive a copy of my written declaration of consent.

- I can end my participation at any time, I don't need to justify myself. Even if I withdraws from the study, my medical care remains assured. The data collected so far These are still being recorded and will be analyzed as part of the study.

- I understand that my participation in the study involves the processing of my personal data. under the conditions described in the information leaflet.

- I understand that my data will be transmitted for this study in coded form only, and that I will not be able to access any other data. pseudonymized. The sponsor ensures that data protection is respected in accordance with to Swiss standards.

- My attending physician is informed of my participation in the study. He or she can share investigators or the health manager some of the data in my medical file that are important for the study. The same applies to the other doctors who follow me.

- The relevant project specialists and the ethics commission can consult my data. not coded for control purposes. All these persons are bound by professional secrecy.

- Réseau Delta's liability insurance covers any damage.

Place, date	Participant's first and last name in block letters<br><br><br>Participant's signature

Geneva study to improve care and quality of life for type 2 diabetes patients (Alliance DT2) in the outpatient setting*: Summary information sheet (Version 3.0 of 11.10.2024)*

Dear Sir/Madam,

On the recommendation of your Delta Network doctor, we invite you to take part in the "Improve" study. the care and quality of life of type 2 diabetes patients (Alliance DT2)". Your participation is completely free. All data collected as part of this study are subject to strict rules and regulations. data protection.

The study was initiated by Delta Network, the study promoter. The latter assumes responsibility, management and financing the study. The scientific part is carried out in partnership with the University of St Gallen and the partner SOKLE, a company specializing in secure medical data management. Financial support has been granted by the Groupe Mutuel health insurer for this study. The latter is providing the data for medico-economic analysis.

Réseau Delta, Ms Cecilia Rios Valente, Health Manager, 59C route de Chancy CH-1213 Petit Lancy
079 557 88 59 (8:30 a.m. to 6 p.m., Monday to Friday) cecilia.rios-valente@reseau-delta.ch

Here are the key points. You can find more detailed information by scanning the QR code.

Why are we conducting this study?

- The number of people with type 2 diabetes is rising all over the world. For for chronic diseases such as diabetes, therapeutic patient education is developing steadily for some fifty years. By using this method, we have been able to observe a decline and even a cure for diabetes. type 2 diabetes (T2D) in several international scientific studies.
- In the Swiss outpatient setting, no studies have yet been carried out to prove the importance of therapeutic patient education for optimal care of T2DM, coupled with questionnaires to assess quality of life in relation to the patient's health situation.
- This study gives patients the opportunity to express their views on their life with the disease, and to choose the activities/tools that seem important for changing lifestyle habits to help control diabetes in collaboration with healthcare professionals (therapeutic alliance).

What should I do if I agree to take part?

- We'll invite you to fill in questionnaires about your health over the course of a year and a half, with the help of if necessary from the health manager, 3 times over 18 months.

→ *Health-related quality of life questionnaires: general EQ-5D (5 questions) and diabetes-related DIAB-Q (17 questions).*

→ *Questionnaire related to experience in the healthcare system - satisfaction questionnaire (9 questions)*

- We will invite you to consult your physician or health care manager to evaluate your rate. glycated haemoglobin, blood pressure and body composition, which will be assessed at the start of the treatment. and at the end of the procedure at 12 months, then 6 months later (3 times over an 18-month period).
- If you decide to participate, you will be part of the control group. This means you'll receive the usual care performed by your attending physician. You will be able to benefit from the other method as soon as the study is completed.

What are the benefits and risks of participating in the study?

PROFITS

- Your commitment will contribute to documenting and analyzing the management of T2DM by healthcare professionals. around you. There are no additional costs for you to participate in the study.
- Through your participation, you contribute to gathering essential information for evaluating and improving the quality of care for type 2 diabetes in family practices.
- Voucher worth CHF 50.00 for full participation in the study by filling in the following form questionnaires.

RISKS & CONSTRAINTS

- By participating in the study, we do not expose you to any risks or constraints.

Application to participate in medical research

Detailed information

Improve the intake in care andthe quality of of life of patients diabétiques de type 2 (DT2) / Value-based care in type 2 diabetes

Simplified title : *Alliance DT2 / Enhancing T2D Care*

Dear Sir/Madam,

We'd like to introduce you to our study and invite you to take part. Before a new method can be disseminated and offered to physicians and other healthcare professionals. Research is needed to find out how this method works.

In medicine, such research is called a **clinical study**. In our study, we want to find out What method of care is provided by several health professionals (doctors...)? nurses, dieticians, specialists in adapted physical activity...) and according to several approaches including therapeutic patient education has on your daily life in your type 2 diabetes requiring treatment. That's why we're asking you if you'd like to participate in this study.

Your participation is voluntary. This **detailed information form** is designed to help you make your decision**.** decision. You can ask any questions you may have during a **consultation with your GP or the people in charge of the study, known as investigators or health managers**. These are responsible for the follow-up of study participants. If you wish to Please sign the **declaration of consent** at the end of this document. By With your signature, you confirm that you have read and understood the information provided. If you do not understand If you have any questions, please do not hesitate to ask the investigators or your doctor for clarification.

The information and consent form consists of three parts:

Part 1The essentials in a nutshell
Part 2Detailed information on the study
Part 3Data protection and insurance coverage

Part 1 gives you an overview of the study. In **Part 2**, we explain in detail the course and context of the study. **Part 3** contains information on the protection of and insurance coverage. By signing the consent form, you certify that you have understood the following and that you agree to participate.

This study is initiated by the Delta Network. This institution is called the promoter. The promoter assumes responsibility, management and financing of a study. The scientific part is carried out in partnership with the University of St. Gallen, technology partner SOKLE, specialist in the secure management of medical data. Financial support is provided by the health insurer Groupe Mutual for this study.

The contact person for this study is:

Ms. Cecilia Rios Valente, Health Manager, Delta Network

Address	59C route de Chancy, CH - 1213 Petit Lancy
Phone	079 557 88 59 (8:30 a.m. to 6 p.m., Monday to Friday)
Email	cecilia.rios-valente@reseau-delta.ch

Part 1: Quick facts

1. Why are we conducting this study?

If you've been living with type 2 diabetes for less than 10 years, we'd like to ask you if you'd like to know whether you wish to participate in this study.

For type 2 diabetes, the standard treatment consists of following the management plan proposed by your healthcare professional. physician, in order to avoid complications such as myocardial infarction, a stroke or a heart attack. cerebrovascular, eye, kidney or nerve damage. No improvement in results measured by blood tests, long-term use of insulin injections, etc. would then become necessary.

In this study, we examine how a new approach to the care of type 2 diabetes can be used to improve the quality of life of patients with type 2 diabetes. 2 in its daily life through clinical measurements and questionnaires. We will evaluate efficacy in terms of disease, quality of life and economics. A a new approach to care would enable you to optimize your treatment and move towards to do this, we first need to measure current practice from the point of view of your diabetes. patient questionnaire. Type 2 diabetes can go into remission.

Find out more about the scientific background to the study **in Chapter 4**.

2. What do you have to do if you take part?

Your participation will last 18 months. You will be called upon by our health manager to help with the study to take certain clinical measurements and complete short questionnaires. At the same time, you will continue your standard treatments (consultation) with your GP.

The average appointment with our health manager lasts less than 20 minutes. Appointments are detailed in **the figure in Chapter 5**.

If you decide to take part, you will receive the same level of care as you do now.

Find out more about the study process and procedures in **Chapter 5**.

3. What are the benefits and risks of participation?

Benefits

The aim of this study is to improve the management of type 2 diabetes and move towards a cure. It It should be stressed that international scientific studies have already demonstrated a direct benefit to the environment. on patients like you (improved laboratory values, increased physical activity...), weight loss, curing diabetes, etc.) thanks to scientific studies. Your participation can help help future diabetic patients. However, you may not be directly cured of your diabetes as a result of participating in this study, but you will be helping to to advance knowledge and science.

Risks

The risks associated with your participation in the study are minimal, if not non-existent, given that you continue to be under the care of your treating physician and that the study will not interfere with your treatments medication (unless it becomes unnecessary or the dosage needs to be lowered because your laboratory data improved diabetes).

Adverse reactions may occur if you choose not to follow your doctor's instructions. doctor or other responsible health professional. This will not be directly related to to this study.

To date, no adverse effects have been reported.

Further information on possible constraints can be found in **chapter 6**.

Part 2: Detailed information on the study

4. Scientific background

4. 1 Background: why are we conducting this study?

Type 2 diabetes (T2D) is a common chronic disease. When you have T2DM, if none of your changes in lifestyle habits, such as eating habits or activity levels is undertaken, the disease progressively worsens and sometimes requires insulin to be taken subcutaneously. as a long-term daily injection. The treatment and management of diabetes therefore depend on a The company is subject to a multitude of parameters, including its own lifestyle.

Diabetes is generally treated with medication and possibly sessions with a diabetes specialist. a dietician and/or physiotherapist.

A great deal of research has already been carried out into the management of type 2 diabetes. Visit studies carried out to date, mainly in hospitals in Switzerland, have shown that interdisciplinary management, i.e. collaboration with healthcare professionals of various specialties, along with therapeutic patient education, is necessary for effective management. of a diabetic patient. In fact, diabetes must be seen as a chronic disorder of the body. and complex way in which our body manages sugar, and for which a patient can with appropriate help (therapeutic alliance) restore lost equilibrium, once diagnosis is established early enough. So we know that, under certain conditions, it is possible to recover from type 2 diabetes.

In this study, we therefore examine whether interdisciplinary care accompanied by education patient is effective in improving clinical parameters such as hemoglobin and body composition (fat, muscle and bone content), as well as the quality of life associated with health compared with standard treatment. And finally, to be able to observe the economic impact of to make this care accessible to all.

4. 2 Structure of the study: how do we proceed?

In our study, participants were randomly assigned according to their treating physicians (assignment This is known as randomization. This method is important for reliable results.

4. 3 Regulation of scientific research involving human beings huma ins

We are carrying out this study in accordance with the laws in force in Switzerland (law relating to research on human beings). (e.g. human rights, data protection laws). In addition, we comply with all applicable internationally recognized standards. The study was reviewed and approved by the relevant ethics committee.

Our study is carried out in the Canton of Geneva. There are several hundred participants affiliated to the Réseau Delta in Geneva.

You will also find a description of this study on the website of the Swiss Federal Office of Public Health. public, at www.kofam.ch, under SNCTP registration number XXX or BASEC number XXX.

5. Course of the study

5. 1 What do you have to do if you take part in the study?

Participation in the study is voluntary and lasts 18 months. You must adhere to the appointment schedule. you → chapter 5.2) as well as all instructions given by the research team and your doctor processing.

You must inform the research team or your treating physician directly.

- if your state of health changes, for example if you are feeling less well or if you new disorders; you must continue to inform him if you withdraw of the study → chapters 5.3 and 5.4);
- For women: if you are planning a pregnancy or are pregnant (risk of gestational diabetes)

5. 2 What happens during appointments?

Appointments are part of your standard diabetes management. In addition to your medical consultation appointments, the health manager (GS) will ask you to take your appointments. clinical measurements and short questionnaires.

As part of the study, you will be asked to complete questionnaires and carry out a number of other tasks. clinical measurements, one year after answering the first questionnaire and then 6 months later (duration total 18 months). Questionnaires can be completed on site or sent electronically. (possibility by telephone if necessary with our health manager) according to your personal choice. Certain clinical measurements (e.g. glycated hemoglobin, blood pressure, body composition) will be taken in the office by the GS or delegated to other healthcare professionals.

Here's what we do at every appointment (with your primary care physician, an educational specialist, etc.) or health manager):

- We answer your questions.
- We ask you questions about your health and quality of life.
- We measure routine clinical parameters
- We can help you fill in questionnaires or make them available to you.

During certain appointments, we also :

- Obtaining a drop of blood from the fingertip for glycated hemoglobin testing
- Blood pressure measurement
- Measuring body composition and body mass index
- Health-related quality of life questionnaire :
    - Generic quality-of-life questionnaire EQ5D (5 questions)
    - Diabetes-specific questionnaire DIAB-Q (17 questions)
- Questionnaires related to experience in the healthcare system
  PREM questionnaire - satisfaction survey (9 questions)

These examinations/questionnaires enable us to assess the effectiveness of the intervention method.

Questionnaires are in electronic format (preferred). Paper format is available on request. available.

To sum up, your participation in the study is structured as follows (Figure 1):

1. Summoned by the health manager or your attending physician to take measurements (glycated hemoglobin, body composition and blood pressure) and sent the short questionnaires (EQ5D and DIAB-Q).
2. One year (12 months) after clinical measurements and completion of questionnaires In the shortest possible time, the health manager will ask you to fill out the questionnaires again. assessment tools (EQ5D and DIAB-Q), as well as the one relating to your experience, called PREM (satisfaction). You will also be called in to take clinical measurements. At the same time cous continue to follow your usual treatment and continue to attend consultations with your GP.
3. Six months later (i.e. 18 months after your first summons), you're called up again by the health manager to complete the evaluation questionnaires (EQ5D and DIAB-Q) and and the one relating to your experience, PREM. You will also be asked to complete the clinical measurements.

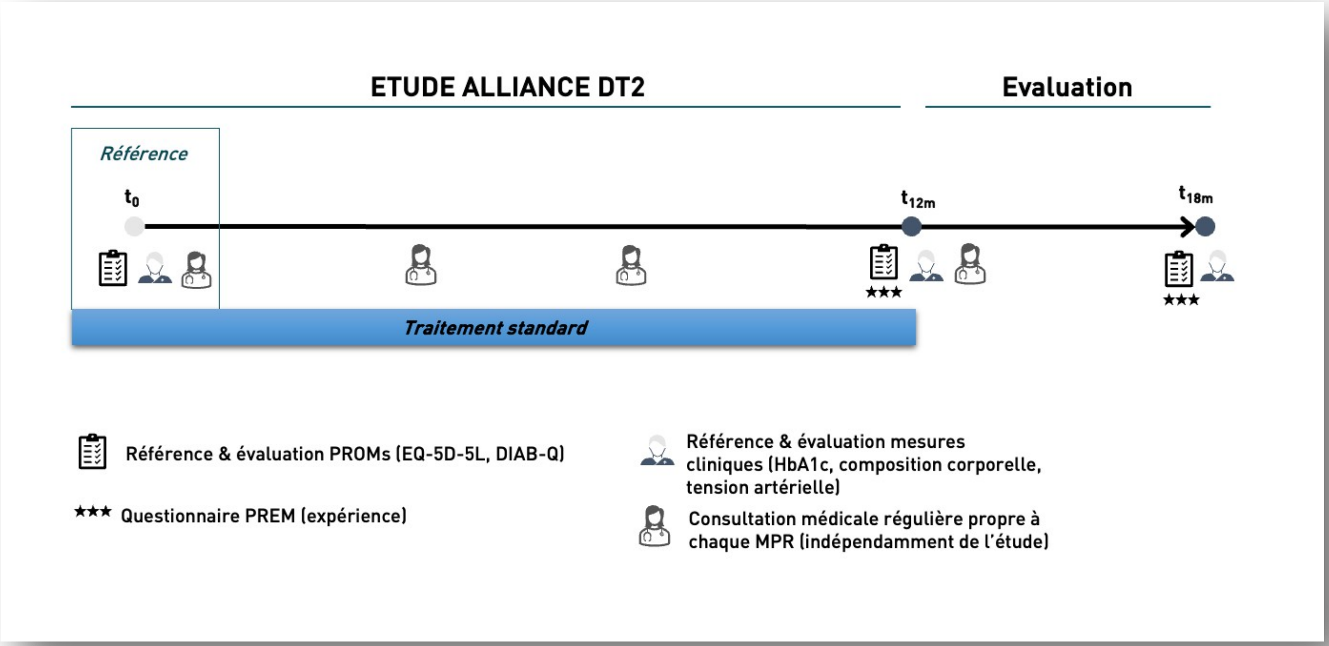

Figure 1 Study procedure for the "control" patient group

You will receive a precise summary of the appointments linked to the study via the health manager. Please note that, in principle, it is not possible to reschedule an appointment. Should you nevertheless need to reschedule an appointment for important reasons, please inform us as soon as possible.

5. 3 What data are processed as part of the study?

For the purposes of this study, you authorize Delta Network via SOKLE to share your insurance number with the Delta Network.
coded with your health insurance. For Groupe Mutuel policyholders, this allows Groupe Mutuel to identify the billing data of the respective participants. Billing data (linked to including medication) are then sent back to the Delta network, which associates them with the code. through SOKLE for the study analyses.

5. 4 When does participation in the study end?

Your participation lasts 18 months (after completion of final questionnaires and measurements).
clinical). You can interrupt your participation at any time before this date → chapter 5.4).
You don't need to justify yourself. If you wish to end your participation, please contact us.
inform the health manager, who will report it to the principal investigator.

If you withdraw from the study, your medical care and treatment will not be affected.
→ chapter 5.4 for other treatment options).

If you withdraw from the study, we can still analyze the data. Your study data remains coded → chapter 9).

5. 5 What happens if you don't want to participate?

If you do not take part in this study, your treatment and medical care will be assured. in line with current standards.

5. 6 Pregnancy

Pregnancy causes physiological changes that disrupt your clinical test results (glycated haemoglobin, body composition, etc.). This means you can't have children. during your participation in the study. This applies to all persons involved in this study. study. You can discuss these questions with your doctor or the investigator. contact.

For people who want to start a pregnancy

You should avoid becoming pregnant while taking part in the study (risk of diabetes). gestational). You must inform your partner(s) that you are taking part in this study. If you are breast-feeding or pregnant, you will not be able to take part in the study.

6. risks, constraints and undesirable effects

6. 1 What are the risks and constraints associated with the study?

Participating in this study does not entail any risk, but there are direct and indirect constraints. only, like any medical treatment of an illness.

If you are currently taking medication, please refer to your doctor's instructions. doctor or pharmacist. The official drug information (package leaflet) is directly accessible on the following website: https://www.swissmedicinfo.ch/?Lang=FR .

6. 2 Risks and constraints associated with examinations in the context of the study

We carry out various medical examinations for this study → chapter 5.2). These examinations are proven procedures for standard treatment by your GP. Nevertheless, they may involve risks and constraints, i.e. they may be unpleasant or have a negative impact on the environment. adverse effects. In this study, the risk/constraint is as follows:

- Fingertip prick/blood sampling: hematomas (when blood is drawn), bruising (when a blood sample is taken). bleeding or swelling may occur at the puncture site.

7. Financing and compensation

This study was initiated by Réseau Delta and is financially supported by Groupe Mutuel. insurance. The University of St. Gallen is the independent research institute commissioned to carry out the analyses. (scientific partner). The technological partner is SOKLE, an IT company based in Geneva. specialized in medical software. For its policyholders, Groupe Mutuel makes available for study billing data in coded form. For policyholders of other funds, access to data is carried out according to contractual possibilities.

There is no financial benefit to the researchers involved in the study.

If you take part in this study, you'll receive a CHF 50 voucher directly for the 3 appointments (questionnaires and clinical metabolic measurements) over the 18-month study period.

Participation in the study entails no additional costs for you or your insurance company. disease.

8. Study results

The results that concern you personally are communicated to you by your attending physician or the health manager.

In addition to individual results, the study will produce global results derived from data from all those involved. This would include, for example, new insights into the factors important for curing type 2 diabetes [illegible]→ chapter 4.1). These results do not apply to you necessarily directly. If you wish, on request, the investigators will provide you with a summary of overall results at the end of the study.

Part 3: Data protection and insurance coverage

9. Data and sample protection

We protect your data (e.g. medical data such as glycated hemoglobin, blood pressure and body composition). Swiss law lays down strict rules governing data protection.

Swiss data protection legislation gives you the right to access, rectify and receive information about your personal data. data collected, processed and transmitted as part of the study. In some cases due to other legal or regulatory requirements, these rights cannot always be exercised. be guaranteed. If you have any questions on this subject, please contact the Network Health Manager Delta for more information.

9. 1 Data and sample coding

All studies generate data from examinations (e.g. glycated hemoglobin, blood pressure, etc.). and body composition). These data are recorded in coded, electronic form. Encryption means that personal information that can directly identify you is stored *separately* from other data, in the form of a list (identification list) that identifies each person with a unique code. Your measured data remain accessible to your doctor (medical software). This means that your name, date of birth or address *are not included.* directly with the other data collected. This identification list remains for 10 years to the Delta Network and is then completely anonymized (user access code required). specific). No one else receives it. Specific exceptions are covered in chapter 9.5.

When we transmit data for this study to the research institute, they are and your personal data is protected. The same applies when the list of policyholders is transmitted to your partner health insurer for billing data matching. Delta network (coding).

9. 2 Data and sample security during the study

The promoter Réseau Delta and the company SOKLE are responsible for the security of your data. of this study. They ensure compliance with current legislation, such as laws on the protection of intellectual property. data. Here is how the study sponsor contractually protects your data:

- -Delta & SOKLE Network Convention
- -Delta Network & Groupe Mutuel agreement, and appendix
- -SOKLE & Groupe Mutuel agreement
  - -Agreement University of St. Gallen & Groupe Mutuel
- -Delta Network & University of St. Gallen data transfer agreement

In this study, your data will be captured and transmitted electronically. The data is stored on a server located in Onex (Geneva, Switzerland) in a Delta Network data center. Only an administrator, who has signed the confidentiality charter for data access, has access to the physical server for maintenance and monitoring. The administrator must be in possession of a and access is recorded. All data is saved daily. After one month, previous backups are moved to an external disk. However, the risk of access to your personal data by unauthorized persons cannot be entirely ruled out (e.g. risk of "hacking").

It may be important for your treating physician to share medical data (clinical value), comorbidity) with the research team and coded data with your health insurer, with your authorization. This also applies to any other doctors who treat you. By signing the consent form, you authorize the communication of this data, if necessary.

9. 3 Data security after the end of the study

When the study is over, the sponsor continues to ensure the security of your data. The law prescribes that all study documents, e.g. data collection forms, should be kept for at least 10 years.

At the end of this long period, the study data remain coded (or, where appropriate, are coded). completely deleted for the research institute). Health data in your file including those in this study, are and always will be available to your healthcare professional. health. In order to access data, user access is required. This excludes use/consultation by third parties.

Once the study is completed, the results are generally published in scientific journals. For this purpose, data is sent in anonymized form to other specialists if necessary. so that they can revise the publication. These data may not be reused for the purposes of research by third parties.

9. 4 Consultation rights during inspections

The conduct of this study may be monitored by the relevant ethics commission. Visit sponsor must also carry out checks to guarantee the quality of the study and its results. For these checks, a small number of specially trained people have access to your

personal data and your medical file. In this case, the data is *not* coded. Anyone consulting your unencrypted data is bound by professional secrecy.

As a participant, you have the right to consult your data at any time.

10. Insurance coverage

You are covered by insurance if you suffer any damage as a result of the study - i.e. The procedure is regulated by law. The procedure is regulated by law. If you feel you have been you have suffered any harm as a result of the study, please contact your doctor or the local hospital directly. health manager or your private insurance company.

If damage results from the correct use of a conventional treatment, the rules of the law apply. are the same as for treatment outside a study. In such In such cases, liability insurance will cover the costs.

Consent declaration for participation in the "Improving the quality of life" study care and quality of life for type 2 diabetes patients (Alliance DT2)" / *Value-based care in type 2 diabetes (Enhancing T2D Care)".*

Please read this form carefully. Do not hesitate to ask us questions if you do not understand. or if you require further information. Your written consent is required for participate.

**BASEC number**	
**Study title**	*Value-based care in type 2* diabetes / Améliorer la prise en soins et la qualité de vie des patients diabétiques de type 2
**Simplified title**	Alliance DT2 / *Enhancing T2D Care*
**Institution responsible**<br>(promoter and address)	Delta SA care network<br>Dr Philippe Schaller, MD, FMH, MPH<br>98 route de Chancy<br>CH - 1213 Onex
**Location**	Canton of Geneva
**Principal Investigator**<br>**Investigator-Coordinator**<br>**Health Manager**	Dr. Minette-Joëlle Zeukeng, PharmD, FPH, PhD<br>Ms. Joëlle Coclet, PhD<br>Ms Cecilia Rios Valente
**Participant**<br>Print name and surname: Date of birth :	

Attestation by attending physician/health manager: I hereby certify that I have explained to the participating in the nature, size and scope of the study. I hereby declare that I have fulfilled all in relation to this study under Swiss law. If I were to become aware, during the course of the study, likely to influence the participant's willingness to take part in the study, I undertake to inform him immediately.

Place,<br>date	First and last name of **attending physician/health manager** in block letters<br><br>Signature

- I have received oral and written information about the study (purpose, progress, benefits & risks) from From my treating physician/investigator/coordinator/health manager undersigned.

- I am voluntarily participating in the study.

- I have had sufficient time to make my decision. I keep written information and I receive a copy of my written declaration of consent.

- I can end my participation at any time, I don't need to justify myself. Even if I withdraws from the study, my medical care remains assured. The data collected so far These are still being recorded and will be analyzed as part of the study.

- I understand that my participation in the study involves the processing of my personal data. under the conditions described in the information leaflet.

- I understand that my data will be transmitted for this study in coded form only, and that I will not be able to access any other data. pseudonymized. The sponsor ensures that data protection is respected in accordance with to Swiss standards.

- My attending physician is informed of my participation in the study. He or she can share investigators or the health manager some of the data in my medical file that are important for the study. The same applies to the other doctors who follow me.

- The relevant project specialists and the ethics commission can consult my data. not coded for control purposes. All these persons are bound by professional secrecy.

- Réseau Delta's liability insurance covers any damage.

Place, date	Participant's first and last name in block letters<br><br>Participant's signature

RESEAU DELTA
LA SANTE INTELLIGENTE

Étude genevoise pour améliorer la prise en soins et la qualité de vie des patients diabétiques de type 2 (Alliance DT2) en milieu ambulatoire*: Résumé feuille d'information intervention* (V3.0 11.10.2024)

Madame, Monsieur,

Sur recommandation de votre médecin traitant du Réseau Delta, nous vous invitons à participer à l'étude « Améliorer la prise en soins et la qualité de vie des patients diabétiques de type 2 (Alliance DT2) ». Votre participation est entièrement libre. Toutes les données collectées dans le cadre de cette étude sont soumises à des règles strictes en matière de protection des données.

Cette étude est initiée par le Réseau Delta, le promoteur de l'étude. Ce dernier assume la responsabilité, la gestion et le financement de l'étude. La partie scientifique est effectuée en partenariat avec l'Université de St Gall et le partenaire technologique est l'entreprise SOKLE, spécialiste dans la gestion sécurisée de données médicales. Un soutien financier est octroyé par l'assureur-maladie Groupe Mutuel pour la réalisation de cette étude. Ce dernier met à disposition les données pour l'analyse médico-économique.

Réseau Delta, Mme Cecilia Rios Valente, gestionnaire en santé, 59C route de Chancy CH-1213 Petit Lancy
079 557 88 59 (de 8h30 à 18h du lundi au vendredi) cecilia.rios-valente@reseau-delta.ch

Voici ci-dessous les points clés. Vous trouverez des informations plus détaillées en scannant le QR code

Pourquoi menons-nous cette étude?

- Le nombre de personnes atteintes de diabète de type 2 ne cesse d'augmenter partout dans le monde. Pour les **maladies chroniques** comme le diabète, **l'éducation thérapeutique au patient** se développe de manière continue depuis plus de 30 ans. En utilisant cette méthode, on a pu observer un recul voire une guérison du diabète de type 2 (DT2) dans plusieurs études scientifiques internationales.
- En Suisse, dans le milieu ambulatoire, il n'existe pas encore d'étude réalisée afin de prouver l'importance de l'éducation thérapeutique au patient pour une prise en soins optimale du DT2, couplée à des **questionnaires spécifiques pour évaluer la qualité de vie liée à la situation de santé du patient**.
- Cette étude permet de donner l'opportunité au patient de donner son avis sur sa vie avec la maladie et de **choisir les activités/outils** qui lui paraissent importants pour changer ses habitudes de vie pour faire reculer son diabète en collaboration avec les professionnels de santé (alliance thérapeutique).

Que dois-je faire si j'accepte de participer ?

- Nous vous inviterons à remplir des questionnaires relatifs à votre santé durant une année, tous **les 3 mois, avec l'aide si nécessaire de la gestionnaire en santé, sur 18 mois**.

→ Questionnaires sur la qualité de vie lié à la santé : général EQ5D (5 questions),lié à l'état dépressif/anxiété PHQ-9 (9 questions) et lié au diabète DIAB-Q (17 questions)

→ Questionnaire lié à l'expérience dans le système de santé - (9 questions) à la fin (12 mois) puis 6 mois après

- Vous serez sollicité par la gestionnaire en santé ou autres professionnels de santé afin d'évaluer votre taux **d'hémoglobine glyquée et votre tension artérielle** ainsi que votre **composition corporelle** sera évaluée au début et à la fin de l'intervention (12 mois) puis 6 mois après (Période totale : 18 mois).
- Si vous décidez de participer, vous ferez partie du groupe bénéficiant de l'intervention. Dans le groupe d'intervention, vous serez traité avec la méthode de prise en soins dispensée par plusieurs professionnels de la santé (diététicien.ne, physiothérapeute ou coach sportif pour la mise en mouvement,…) et selon plusieurs approches incluant de l'éducation thérapeutique au patient.

Quels sont les bénéfices et les risques liés à la participation à l'étude ?

BENEFICES

- **Personnaliser et optimiser votre prise en soins** du DT2 par les professionnels de santé autour de vous. La participation à l'étude n'entraîne aucun coût supplémentaire pour vous. Certaines activités proposées non prise en charge par votre assurance maladie sont **financées par le Réseau Delta**.
- Par votre participation, vous contribuez à récolter des informations essentielles pour évaluer et améliorer la qualité de **prise en soins du diabète de type 2 en cabinet de médecine de famille.**
- **Vous recevrez un lot de bienvenu d'accessoires « essentiels » (gourde, bandes élastiques, tablier).**

RISQUES & CONTRAINTES

- En participant à l'étude, nous vous exposons à aucun risque ou contrainte.

Demande de participation à une recherche médicale

INTERVENTION Informations détaillées

Améliorer la prise en soins et la qualité de vie des patients diabétiques de type 2 (DT2) / Value-based care in type 2 diabetes

Titre simplifié : *Alliance DT2 / Enhancing T2D Care*

Madame, Monsieur,

Nous aimerions vous présenter notre étude et vous inviter à y participer. Avant qu'une nouvelle méthode de prise en soins puisse être diffusée et proposée auprès des médecins et des autres professionnels de la santé, des recherches sont en effet nécessaires pour savoir comment cette méthode agit.

En médecine, une telle recherche s'appelle une **étude clinique**. Dans notre étude, nous voulons découvrir quel effet la méthode de prise en soins dispensée par plusieurs professionnels de la santé (médecins, infirmières et infirmiers, diététiciennes et diététiciens, spécialistes en activité physique adaptée …) et selon plusieurs approches incluant de l'éducation thérapeutique au patient a sur votre quotidien dans votre diabète de type 2 et qui nécessite un traitement. C'est pourquoi nous vous demandons si vous souhaitez participer à cette étude.

Votre participation est volontaire. Ce **formulaire d'information détaillé** doit vous aider à prendre votre décision. Vous pouvez poser toutes vos questions lors d'un **entretien avec votre médecin traitant ou les personnes en charge de l'étude que l'on nomme investigateurs ou gestionnaire de santé**. Ces derniers sont responsables du suivi des participantes et des participants de l'étude. Si vous souhaitez participer, nous vous remercions de signer la **déclaration de consentement** à la fin du document. Par votre signature, vous attestez avoir lu et compris les informations fournies. Si vous ne comprenez pas quelque chose, n'hésitez pas à demander des précisions aux investigateurs ou à votre médecin traitant.

Le formulaire d'information et de consentement se compose de trois parties :

Partie 1 **L'essentiel en bref**
Partie 2 **Informations détaillées sur l'étude**
Partie 3 **Protection des données et couverture d'assurance**

Dans la **partie 1**, vous avez un aperçu général de l'étude. Dans la **partie 2**, nous vous expliquons en détail le déroulement et le contexte de l'étude. La **partie 3** contient les informations relatives à la protection des données et à la couverture d'assurance. En signant le consentement, vous attestez que vous avez compris toutes les informations et que vous acceptez de participer.

Cette étude est initiée par le Réseau Delta. Cette institution est appelée promoteur. Le promoteur assume la responsabilité, la gestion et le financement d'une étude. La partie scientifique est effectuée en partenariat avec l'Université de St Gall, l'entreprise SOKLE partenaire technologique spécialiste dans la gestion sécurisée de données médicales. Un soutien financier est octroyé par l'assureur maladie Groupe Mutuel pour la réalisation de cette étude.

La personne de contact pour cette étude est :

Madame Cecilia Rios Valente, gestionnaire en santé, Réseau Delta

Adresse 59C route de Chancy, CH - 1213 Petit Lancy
Téléphone 079 557 88 59 (de 8h30 à 18h du lundi au vendredi)
Courriel cecilia.rios-valente@reseau-delta.ch

Partie 1 : L'essentiel en bref

1. Pourquoi menons-nous cette étude ?

Vous vivez avec un diabète de type 2 depuis moins de 10 ans et c'est pourquoi nous vous demandons si vous souhaitez participer à cette étude.

Pour le diabète de type 2, le traitement standard consiste à suivre la prise en charge proposée par votre médecin traitant, dans le but d'éviter les complications telles que l'infarctus du myocarde, un accident vasculaire cérébral ou des lésions oculaires, rénales ou nerveuses. Sans amélioration des résultats cliniques mesurés notamment lors de prises de sang, une utilisation au long court d'injection d'insuline quotidienne deviendrait alors nécessaire.

Dans cette étude, nous examinons comment une nouvelle méthode de prise en soins du diabète de type 2 dans son quotidien dispensée par plusieurs professionnels de la santé (médecins, infirmières et infirmiers, diététiciennes et diététiciens, spécialistes en activité physique adaptée …) et selon plusieurs approches incluant l'éducation thérapeutique au patient. Nous allons évaluer son efficacité au niveau de la maladie, la qualité de vie et également sous l'angle économique. Cette prise en soins permettrait d'optimiser votre traitement afin de tendre vers la guérison de votre diabète. La rémission du diabète de type 2 est possible.

Vous en apprendrez davantage sur le contexte scientifique de l'étude au **chapitre 4**.

2. Que devez-vous faire si vous participez ?

Votre participation durera 18 mois. Un suivi sera effectué durant une année tous les 3 mois, puis à 6 mois. Une séance de Cercles de qualité (CQ) médecin-patient en groupe sera organisée avec votre médecin traitant. Un certificat médical d'une demi-journée pourra vous être délivré afin de participer à la séance CQ. Vous serez sollicité par notre gestionnaire en santé dans le cadre de l'étude afin de prendre certaines mesures clinique et remplir des questionnaires courts. En parallèle vous poursuivrez vos traitements standards (consultation) avec votre médecin traitant.

Un rendez-vous avec notre gestionnaire en santé dure en moyenne moins de 20 minutes. Les rendez-vous sont détaillés dans **la figure au chapitre 5**.

Si vous décidez de participer, la prise en soins reste identique à celle que vous avez actuellement avec en plus la possibilité de choisir des activités optionnelles à choix pour la mise en mouvement, la diététique ou en lien avec la santé mentale (prestations non prises en charges par l'assurance de base incluses).

Vous en apprendrez plus sur le déroulement et les procédures de l'étude au **chapitre 5**.

3. Quels sont les bénéfices et les risques liés à la participation ?

Bénéfices

Cette étude a pour but d'améliorer la prise en soins du diabète de type 2 pour tendre vers la guérison. Il convient de souligner que des études scientifiques internationales ont déjà montrés un bénéfice direct sur des patients comme vous (amélioration des valeurs de laboratoire, augmentation de l'activité physique, perte de poids, guérison du diabète, etc.). Votre participation peut dans tous les cas contribuer à aider de futurs patients. Vous n'allez peut-être toutefois pas être guéri de votre diabète à la suite de votre participation à cette étude mais vous allez contribuer à faire avancer vos connaissances et la science.

Risques

La méthode de prise en soins est nouvelle en milieu ambulatoire et n'a pas encore été testée en Suisse. Toutefois les risques sont minimes pour ne pas dire inexistants étant donné que vous continuez à être suivi par votre médecin traitant et que l'étude n'interféra pas avec votre traitement médicamenteux (excepté s'il devient inutile ou le dosage doit être abaissé car vos données de laboratoire sont améliorées).

Des effets indésirables peuvent survenir si vous choisissez de ne pas respecter les indications de votre médecin traitant ou tout autres professionnel de la santé responsable. Ceci ne sera pas directement lié à la présente étude.

Nous ne connaissons peut-être pas encore tous les risques et les effets indésirables de la méthode d'intervention, toutefois jusqu'à présent, aucun effet indésirable n'a été répertorié.

Vous trouverez davantage d'informations sur les éventuelles contraintes au **chapitre 6**.

Partie 2 : Informations détaillées sur l'étude

4. Contexte scientifique

4.1 Contexte : pourquoi menons-nous cette étude ?

Le diabète de type 2 (DT2) est une maladie chronique fréquente. Lorsque l'on est atteint de DT2, si aucun changement des habitudes de vie comme le comportement au niveau alimentaire ou au niveau de l'activité physique n'est entrepris, la maladie s'aggrave progressivement et nécessite parfois la prise d'insuline sous forme d'injection quotidienne à long terme. Le traitement et la gestion du diabète dépendent donc d'une multitude de paramètres, liés notamment à son propre mode de vie.

Le diabète est généralement traité avec des médicaments et éventuellement des séances avec une personne spécialisée en diététique et/ou spécialisée en physiothérapie.

Il existe déjà un nombre important de recherches concernant la prise en charge du diabète de type 2. Les études menées jusqu'à présent notamment en Suisse, principalement en milieu hospitalier, ont montré qu'une prise en charge interdisciplinaire c'est-à-dire en collaboration avec des professionnels de la santé de diverses spécialités, avec l'éducation thérapeutique au patient, est nécessaire pour une prise en charge optimale d'un patient diabétique. En effet, le diabète doit être considéré comme un dérèglement chronique et complexe du fonctionnement de notre organisme dans la gestion du sucre et pour lequel un patient peut avec une aide adéquate (alliance thérapeutique) rétablir l'équilibre perdu, lorsque le diagnostic est établi suffisamment précocement. Ainsi, nous savons donc que sous certaines conditions, il est possible de guérir de son diabète de type 2.

Nous examinons donc dans cette étude si une prise en soins interdisciplinaire accompagnée d'éducation thérapeutique au patient est efficace pour améliorer les paramètres cliniques tels que l'hémoglobine glyquée et la composition corporelle (part de graisse, de muscle et d'os) ainsi que la qualité de vie liée à la santé. Et finalement, pouvoir observer l'impact économique de cette prise en soins dans un but de la rendre accessible à tous.

4.2 Structure de l’étude : comment procédons-nous ?

Dans notre étude, les participants selon leurs médecins traitants sont répartis au hasard (affectation aléatoire) dans des groupes, c’est ce qu’on appelle la randomisation. Cette méthode est importante pour obtenir des résultats fiables. Chaque groupe reçoit un traitement différent.

4.3 Réglementation de la recherche scientifique impliquant des êtres humains

Nous réalisons cette étude conformément aux lois en vigueur en Suisse (loi relative à la recherche sur l’être humain, lois sur la protection des données). En outre, nous respectons toutes les directives reconnues au niveau international. La commission d’éthique compétente a examiné et autorisé l’étude.

Notre étude est effectuée dans la Canton de Genève. Il y a plusieurs centaines de participants affiliés au Réseau Delta à Genève qui sont sollicités.

Vous trouverez également une description de cette étude sur le site Internet de l’Office fédéral de la santé publique, à l’adresse www.kofam.ch, sous le numéro d’enregistrement SNCTP XXX ou le numéro BASEC XXX.

5. Déroulement de l'étude

5.1 Que devez-vous faire si vous participez à l'étude ?

La participation à l'étude est volontaire et dure 18 mois. Vous devez respecter le calendrier des rendez-vous (→ chapitre 5.2) ainsi que toutes les consignes données par l'équipe de recherche et votre médecin traitant.

Vous devez informer l'équipe de recherche ou directement votre médecin traitant

- si votre état de santé évolue, par exemple si vous vous sentez moins bien ou si vous présentez de nouveaux troubles ; vous devez continuer de l'en informer si vous vous retirez de l'étude (→ chapitres 5.3 et 5.4) ;
- Pour les femmes : si vous envisagez une grossesse ou êtes enceinte (risque de diabète gestationnel)

5.2 Que se passe-t-il lors des rendez-vous ?

Une première consultation de départ Cercles de qualité patient-médecin incluant votre médecin traitant et la gestionnaires en santé est obligatoire.

Les rendez-vous font partie de votre prise en charge standard de suivi de diabète. En marge de vos rendez-vous de consultation médicales, la gestionnaire en santé (GS) peut vous solliciter pour des rendez-vous relatif aux prises de vos mesures cliniques et concernant les questionnaires.

Dans le cadre de l'étude, vous serez sollicités pour répondre à des questionnaires tous les 3 mois durant une année puis à 6 mois, sur place ou par voie électronique (possibilité par téléphone si nécessaire) avec notre gestionnaire en santé, selon votre choix personnel. Certaines mesures cliniques (notamment hémoglobine glyquée, tension artérielle, composition corporelle) seront prises au cabinet par la GS ou déléguées à d'autres professionnels de la santé. D'autres rendez-vous supplémentaires pourront être agendé notamment avec une diététicienne, physiothérapeute, professionnel en activité physique adaptée, psychothérapeute selon les cas et selon votre désir sur la base de votre programme DT2 individualisés. Des activités en groupe pourront également être effectuée selon vos désirs. L'ensemble des activités proposées seront prises en charge par votre assurance de base ou selon l'activité, notamment celle en groupe, par le Réseau Delta. Le Réseau Delta prendra en charge la quote-part des activités prises en charges par l'assurance qui auront été initiés dans le cadre de l'étude. Un rendez-vous peut durer entre environ 20 minutes (simple consultation) et jusqu'à 2 heures (cours en groupe de diététique par exemple). La liste des étapes pour l'intervention figure dans la figure ci-après.

Voici ce que nous faisons à tous les rendez-vous (avec votre médecin traitant, un spécialiste en éducation thérapeutique au patient ou la gestionnaire en santé):

- Nous répondons à vos questions.
- Nous vous posons des questions sur votre état de santé et votre qualité de vie.
- Nous mesurons des paramètres cliniques de routine
- Nous vous aidons à remplir des questionnaires ou les mettons à votre disposition.

Lors de certains rendez-vous, nous faisons en outre :

- Prélèvement d'une goutte de sang obtenue par piqûre au bout du doigt pour l'hémoglobine glyquée
- Prise de la tension artérielle
- Mesure de la composition corporelle et indice de masse corporelle
- Questionnaire sur la qualité de vie lié à la santé :
  - Questionnaire générique lié à la qualité de vie EQ5D (5 questions)
  - Questionnaire spécifique lié à l'anxiété dépression PHQ-9 (9 questions)
  - Questionnaire spécifique lié au diabète DIAB-Q (17 questions)
- Questionnaires lié à l'expérience dans le système de santé

  Questionnaire PREM - questionnaire de satisfaction (9 questions)

Ces examens/questionnaires nous permettent d'évaluer l'efficacité de la méthode d'intervention.

Les questionnaires sont sous forme électronique (à privilégier). Sur demande, le format papier est disponible.

En résumé le déroulement de votre participation à l'étude s'articule comme suit (Figure 1):

1. Organisation d'un Cercle de qualité (CQ) médecins-patients (séance en groupe) durant laquelle vous discuter des différents aspects du diabète de type 2 avec votre médecin et un médecin spécialiste en éducation thérapeutique du patient pour la première partie (environ 1h30). Lors de la deuxième partie (env.0h30). La gestionnaire en santé vous présente les différentes activités optionnelles à choix. Avec cette dernière, vous déterminer un programme individuelle DT2 selon vos souhaits.
2. Directement à la suite du CQ, la gestionnaire en santé prend les différentes mesures cliniques (hémoglobine glyquée, composition corporelle et tension artérielle) et vous soumet le premier set de questionnaires (EQ5D et DIAB-Q) que vous pouvez remplir directement sur place ou à la maison.
3. Trois mois après le CQ vous serez à nouveau sollicité pour répondre aux questionnaires courts (DIAB-Q et PHQ-9). Ceci également à 6 mois et 9 mois. En parallèle, vous suivez les activités/prestations que vous avez choisis et vous vous rendez chez votre médecin à la fréquence habituelle. Lors des consultations, vous avez la possibilité de discuter avec votre médecin des questionnaires. Ce dernier aura la possibilité de les consulter à travers la plateforme SOKLE.
4. Une année (12 mois) après le CQ, vous êtes à nouveau invité par la gestionnaire en santé à remplir les questionnaire d'évaluation (EQ5D et DIAB-Q) ainsi que celui relatif à votre expérience, nommé PREM (satisfaction). Vous êtes également convoqué afin d'effectuer les mesures cliniques. En parallèle vous continuez de suivre les activités optionnelles que vous avez choisis.
5. Six mois plus tard, vous êtes à nouveau sollicité par la gestionnaire en santé afin de remplir les questionnaire d'évaluation (EQ5D et DIAB-Q) ainsi que celui relatif à votre expérience, nommé PREM (satisfaction). Vous êtes également convoqué afin d'effectuer les mesures cliniques.

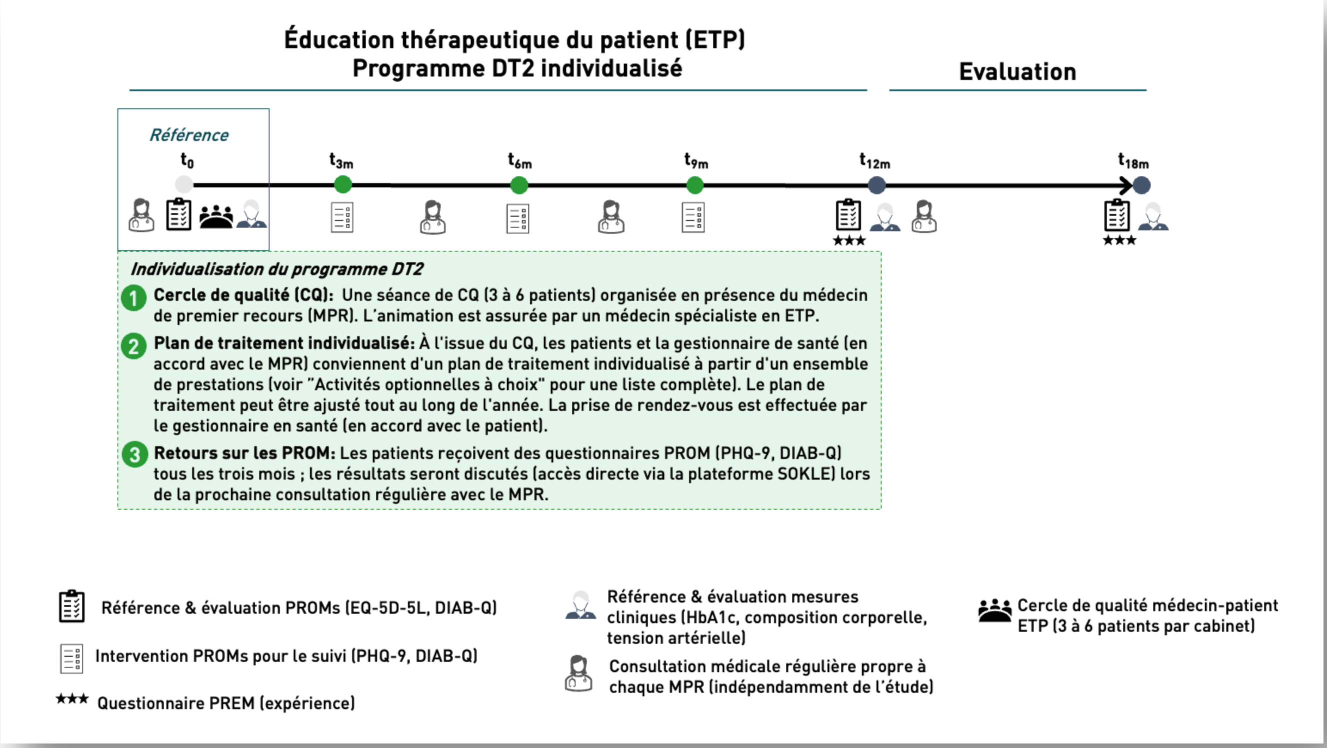

Figure 1 Déroulement de l'étude pour le groupe de patient "intervention"

Vous recevrez un récapitulatif précis des rendez-vous liés à l'étude par le biais de la gestionnaire en santé. Merci de noter qu'il n'est en principe pas possible de reporter un rendez-vous. Si vous deviez malgré tout déplacer un rendez-vous pour des raisons importantes, nous vous prions de nous en informer rapidement.

5.3 Quelles données sont traitées dans le cadre de l'étude ?

Dans le cadre de l'étude, vous autorisez le Réseau Delta via SOKLE à partager votre numéro d'assurance codé avec votre assurance-maladie. Pour les assurés du Groupe Mutuel cela permet au Groupe Mutuel d'identifier les données de facturation des participants respectifs. Les données de facturation (notamment liées à votre médications) sont ensuite renvoyées au Réseau Delta qui les associe au code correspondant par l'intermédiaire de SOKLE pour les analyses de l'étude.

5.4 Quand la participation à l'étude prend-elle fin ?

Votre participation dure 18 mois (après avoir complété les derniers questionnaires et prise de mesures cliniques). Vous pouvez interrompre votre participation à tout moment avant cette date (→ chapitre 5.4). Vous n'avez pas besoin de vous justifier. Si vous souhaitez mettre fin à votre participation, veuillez en informer la gestionnaire en santé qui le rapportera à l'investigatrice principale.

Si vous vous retirez de l'étude, cela n'aura aucune incidence sur vos soins médicaux et votre traitement (→ chapitre 5.4 pour les autres possibilités de traitement).

Si vous vous retirez de l'étude, nous pourrons encore analyser les données. Vos données d'étude restent codées (→ chapitre 9).

5.5 Que se passe-t-il si vous ne souhaitez pas participer ?

Si vous ne participez pas à cette étude, votre traitement et votre prise en charge médicale seront assurés conformément aux standards actuels.

5.6 Grossesse

La grossesse engendre des changement physiologiques qui perturbe vos résultats cliniques d'analyse (hémoglobine glyquée, composition corporelle, etc…). Ainsi, vous ne pouvez donc pas avoir d'enfants pendant votre participation à l'étude. Cela vaut pour les personnes concernées qui participent à cette étude. Vous pouvez discuterez de ces questions avec votre médecin traitant ou la personne investigatrice de contact.

Pour les personnes concernées qui veulent débuter une grossesse

Vous devez éviter de débuter une grossesse pendant votre participation à l'étude (risque de diabète gestationnel). Vous devez informer votre partenaire / vos partenaires que vous prenez part à cette étude. Si vous allaitez ou êtes enceinte, vous ne pourrez pas participer à l'étude.

6. Risques, contraintes et effets indésirables

6.1 Quels sont les risques et les contraintes liés à l'étude ?

La participation à cette étude ne comporte pas de risque et des contraintes directes mais indirecte uniquement, comme tout traitement médical d'une maladie.

Si vous prenez actuellement des médicaments, nous vous prions de vous référez aux indications de votre médecin traitant ou votre pharmacien. L'information officielle du médicament (notice d'emballage) est directement accessible sur le site internet suivant : https://www.swissmedicinfo.ch/?Lang=FR .

6.2 Risques et contraintes liés aux examens dans le cadre de l'étude

Nous effectuons différents examens d'ordre médical pour cette étude (→ chapitre 5.2). Ces examens sont des procédures éprouvées relatives à un traitement standard par votre médecin traitant. Néanmoins, ils peuvent comporter des risques et des contraintes, c'est-à-dire qu'ils peuvent être désagréables ou avoir des effets indésirables. Dans cette étude, le risque/contrainte est le suivant :

- Piqûre au bout du doigt/prise de sang : des hématomes (lors d'une prise de sang), des saignements ou des gonflements peuvent survenir au point de piqûre/ponction.

7. Financement et indemnisation

Cette étude est initiée par le Réseau Delta et est soutenue financièrement pour le Groupe Mutuel assurance. L'Université de St Gall est l'institut de recherche indépendant mandaté pour les analyses (partenaire scientifique). Le partenaire technologique est SOKLE, une société d'informatique genevoise spécialisée dans les logiciels médicaux. Pour ses assurés, le Groupe mutuel met à disposition pour l'étude les données de facturation sous forme codées. Pour les assurés d'autres caisses, l'accès aux données s'effectue selon les possibilités contractuelles.

Les chercheurs participant à l'étude n'en retirent aucun avantage financier.

Si vous participez à cette étude, vous ne recevrez pas directement d'argent mais une compensation (pack de bienvenu « les essentiels – gourde - tablier – élastique », offres de cours/prestations non prise en charge par l'assurance de base, remboursement de la quote-part des prestations directement liées à l'étude).

La participation à l'étude n'entraîne aucun coût supplémentaire pour vous ni pour votre assurance-maladie. Certaines activités proposées qui ne sont pas prise en charge par l'assurance de base sont financées par le Réseau Delta.

8. Résultats de l'étude

Les résultats qui vous concernent personnellement vous sont communiqués par votre médecin traitant ou la gestionnaire en santé.

En plus des résultats individuels, l'étude produira des résultats globaux qui proviennent des données de toutes les personnes y participant. Il s'agirait p.ex. de nouvelles connaissances sur les facteurs/activités important/e/s pour guérir du diabète de type 2 (→ chapitre 4.1). Ces résultats ne vous concernent pas nécessairement directement. Si vous le souhaitez, sur demande, les investigateurs vous transmettront un résumé des résultats globaux à la fin de l'étude.

Partie 3 : Protection des données et couverture d'assurance

9. Protection des données et des échantillons

Nous protégeons vos données (p. ex. les données médicales telles que l'hémoglobine glyquée, la tension artérielle et la composition corporelle). Les lois suisses prévoient des règles strictes en matière de protection des données.

La législation suisse sur la protection des données vous donne le droit d'accéder, de rectifier et de recevoir les données qui ont été collectées, traitées et transmises dans le cadre de l'étude. Dans des cas exceptionnels en raison d'autres exigences légales ou réglementaires, ces droits ne peuvent pas toujours être garantis. Si vous avez des questions à ce sujet, veuillez contacter la gestionnaire en santé du Réseau Delta qui vous renseignera.

9.1 Codage des données et des échantillons

Toute étude génère des données issues des examens (p. ex. l'hémoglobine glyquée, la tension artérielle et la composition corporelle). Ces données sont enregistrées de manière codée, sous forme électronique. Le codage signifie que les informations personnelles qui peuvent vous identifier directement sont conservées *séparément* des autres données, sous la forme d'une liste (liste d'identification) qui identifie chaque personne avec un code unique. Ainsi, votre nom, votre date de naissance ou votre adresse *ne figurent pas* directement avec les autres données collectées. Cette liste d'identification reste pendant 10 années auprès du Réseau Delta et est ensuite totalement anonymisée (nécessité d'un code d'accès utilisateur spécifique). Personne d'autre ne la reçoit. Les exceptions particulières sont réglées au chapitre 9.4.

Lorsque nous transmettons des données dans le cadre de cette étude à l'institut de recherche, elles sont toujours codées et vos données personnelles sont protégées. Il en va de même lorsque la liste des assurés est transmise pour le couplage des données de facturation avec votre assureur maladie partenaire du Réseau Delta (codification).

9.2 Sécurité des données et des échantillons pendant l'étude

Le promoteur Réseau Delta ainsi que la société SOKLE sont responsables de la sécurité de vos données de cette étude. Ils veillent au respect des lois en vigueur, par exemple des lois sur la protection des données. Voici comment le promoteur de l'étude protège contractuellement vos données :

- Convention Réseau Delta & SOKLE
- Convention Réseau Delta & Groupe Mutuel et annexe
- Convention SOKLE & Groupe Mutuel
- Convention Université de St Gall & Groupe Mutuel
- Convention pour le transfert des données Réseau Delta & Université de St Gall

Dans cette étude, vos données sont saisies et transmises par voie électronique. Les données sont stockées sur un serveur situé à Onex (Genève, Suisse) dans un centre de données du Réseau Delta. Seul un administrateur, qui a signé la charte de confidentialité pour l'accès aux données, a accès au serveur physique pour la maintenance et la surveillance. L'administrateur doit être muni d'une carte d'identité et l'accès est enregistré. Toutes les données sont sauvegardées chaque jour. Au bout d'un mois, les sauvegardes précédentes sont déplacées sur un disque externe. Néanmoins, le risque que des personnes non autorisées accèdent à vos données personnelles ne peut être entièrement exclu (p. ex. risque de « piratage informatique »).

Il peut être important que votre médecin traitant partage des données médicales anonymisées avec l'équipe de recherche et des données codées avec votre assureur maladie partenaire, avec votre autorisation. Cela vaut également pour les éventuels autres médecins qui vous suivent. En signant le consentement, vous autorisez la communication de ces données, si nécessaire.

9.3 Sécurité des données après la fin de l'étude

Lorsque l'étude est terminée, le promoteur continue d'assurer la sécurité de vos données. La loi prescrit que tous les documents de l'étude, par exemple les formulaires de collecte de données, doivent être conservés pendant au moins 10 ans.

Au terme de cette longue période, les données de l'étude restent codées (ou, le cas échéant, sont entièrement anonymisées pour l'institut de recherche). Les données relatives à la santé de votre dossier médical, y compris celles de cette étude, sont et resteront toujours accessibles à vos soignants. Afin d'avoir accès aux données, il est nécessaire d'avoir un accès utilisateur. Ceci exclue l'utilisation/consultation par des tiers.

Une fois l'étude terminée, les résultats de l'étude sont généralement publiés dans des revues scientifiques. Pour ce faire, les données sont si nécessaires envoyées sous forme anonymisée à d'autres spécialistes afin qu'ils puissent réviser la publication. Une réutilisation de ces données est possible pour des analyses secondaires ou d'autres questions de recherche futures mais ne peuvent cependant pas être réutilisées par des tiers.

9.4 Droit de consultation lors des contrôles

La réalisation de cette étude peut faire l'objet de contrôles par la commission d'éthique compétente. Le promoteur doit également procéder à des vérifications afin de garantir la qualité de l'étude et de ses résultats. Pour ces contrôles, un petit nombre de personnes spécialement formées ont accès à vos

données personnelles et à votre dossier médical. Dans ce cadre, les données ne sont donc *pas* codées. Les personnes qui consultent vos données non codées sont soumises au secret professionnel.

En tant que participant·e, vous avez à tout moment le droit de consulter vos données.

10. Couverture d'assurance

Vous bénéficiez d'une couverture d'assurance si vous subissez un dommage du fait de l'étude – c'est-à-dire du fait de la méthode de prise en soins. La procédure est réglée par la loi. Si vous pensez avoir subi un dommage du fait de l'étude, veuillez-vous adresser directement à votre médecin traitant ou à la gestionnaire en santé ou à votre assurance privée.

Si un dommage résulte de l'utilisation conforme de l'application d'un traitement conventionnel, les règles en matière de responsabilité sont les mêmes que pour les traitements en dehors d'une étude. En pareil cas, l'assurance responsabilité civile prend en charge les frais.

RESEAU DELTA
LA SANTE INTELLIGENTE

Déclaration de consentement pour la participation à l’étude « Améliorer la prise en soins et la qualité de vie des patients diabétiques de type 2 (Alliance DT2) » / *Value-based care in type 2 diabetes (Enhancing T2D Care)* »

Veuillez lire attentivement ce formulaire. N’hésitez pas à nous poser des questions si vous ne comprenez pas quelque chose ou si vous souhaitez des précisions. Votre consentement écrit est nécessaire pour participer.

**Numéro BASEC**	
**Titre de l’étude**	Améliorer la prise en soins et la qualité de vie des patients diabétiques de type 2 / *Value-based care in type 2 diabetes*
**Titre simplifié**	Alliance DT2 / *Enhancing T2D Care*
**Institution responsable** (promoteur et adresse)	Réseau de soins Delta SA<br>Dr Philippe Schaller, MD, FMH, MPH<br>98 route de Chancy<br>CH - 1213 Onex
**Lieu de réalisation**	Canton de Genève
**Investigatrice-principale**<br>**Investigatrice-coordinatrice**<br>**Gestionnaire en santé**	Dre Minette-Joëlle Zeukeng, PharmD, FPH, PhD<br>Mme Joëlle Coclet, PhD<br>Mme Cecilia Rios Valente
**Participant**<br>Nom et prénom en caractères d’imprimerie :<br>Date de naissance :	

Attestation du médecin traitant/gestionnaire en santé : Par la présente, j’atteste avoir expliqué au participant la nature, l’importance et la portée de l’étude. Je déclare satisfaire à toutes les obligations en relation avec cette étude selon le droit suisse. Si je devais prendre connaissance, au cours de l’étude, d’éléments susceptibles d’influer sur la disposition du participant à prendre part à l’étude, je m’engage à l’en informer immédiatement.

Lieu, date	Nom et prénom du **médecin traitant/gestionnaire en santé** en caractères d’imprimerie<br><br>Signature

- J'ai reçu des informations orales et écrites sur l'étude (but, déroulement, bénéfices & risques) de la part de mon médecin traitant traitant/investigatrice-coordinatrice/gestionnaire en santé soussigné.e.

- Je participe volontairement à l'étude.

- J'ai eu suffisamment de temps pour prendre ma décision. Je conserve les informations écrites et je reçois une copie de ma déclaration de consentement écrite.

- Je peux mettre fin à ma participation à tout moment, je n'ai pas besoin de me justifier. Même si je me retire de l'étude, ma prise en charge médicale reste assurée. Les données collectées jusque-là restent enregistrés et seront encore analysés dans le cadre de l'étude.

- J'ai compris que ma participation à l'étude implique le traitement de mes données personnelles dans les conditions décrites dans la notice d'information.

- J'ai compris que mes données ne sont transmises pour cette étude que sous forme codée et pseudanonymisée. Le promoteur veille au respect de la protection des données conformément aux normes suisses.

- Mon médecin traitant est informé de ma participation à l'étude. Il peut partager avec les investigatrices ou la gestionnaire en santé certaines des données de mon dossier médical qui sont importantes pour l'étude. Cela vaut également pour les autres médecins qui me suivent.

- Les spécialistes compétents du projet et la commission d'éthique peuvent consulter mes données non codées à des fins de contrôle. Toutes ces personnes sont soumises au secret professionnel.

- L'assurance responsabilité civile de l'institution Réseau Delta couvre les dommages éventuels.

Lieu, date	Nom et prénom du participant en caractères d'imprimerie<br><br><br>Signature du participant

RESEAU DELTA
LA SANTE INTELLIGENTE

Étude genevoise pour améliorer la prise en soins et la qualité de vie des patients diabétiques de type 2 (Alliance DT2) en milieu ambulatoire*: Résumé feuille d'information* *(Version 3.0 du 11.10.2024)*

Madame, Monsieur,

Sur recommandation de votre médecin traitant du Réseau Delta, nous vous invitons à participer à l'étude « Améliorer la prise en soins et la qualité de vie des patients diabétiques de type 2 (Alliance DT2) ». Votre participation est entièrement libre. Toutes les données collectées dans le cadre de cette étude sont soumises à des règles strictes en matière de protection des données.

Cette étude est initiée par le Réseau Delta, le promoteur de l'étude. Ce dernier assume la responsabilité, la gestion et le financement de l'étude. La partie scientifique est effectuée en partenariat avec l'Université de St Gall et le partenaire technologique est l'entreprise SOKLE, spécialiste dans la gestion sécurisée de données médicales. Un soutien financier est octroyé par l'assureur-maladie Groupe Mutuel pour la réalisation de cette étude. Ce dernier met à disposition les données pour l'analyse médico-économique.

Réseau Delta, Mme Cecilia Rios Valente, gestionnaire en santé, 59C route de Chancy CH-1213 Petit Lancy
079 557 88 59 (de 8h30 à 18h du lundi au vendredi) cecilia.rios-valente@reseau-delta.ch

Voici ci-dessous les points clés. Vous trouverez des informations plus détaillées en scannant le QR code

Pourquoi menons-nous cette étude?

- Le nombre de personnes atteintes de diabète de type 2 ne cesse d'augmenter partout dans le monde. Pour les **maladies chroniques** comme le diabète, **l'éducation thérapeutique au patient** se développe de manière continue depuis une cinquantaine d'années. En utilisant cette méthode, on a pu observer un recul voire une guérison du diabète de type 2 (DT2) dans plusieurs études scientifiques internationales.
- En Suisse, dans le milieu ambulatoire, il n'existe pas encore d'étude réalisée afin de prouver l'importance de l'éducation thérapeutique au patient pour une prise en soins optimale du DT2, couplée à des **questionnaires spécifiques pour évaluer la qualité de vie liée à la situation de santé du patient**.
- Cette étude permet de donner l'opportunité au patient de donner son avis sur sa vie avec la maladie et de **choisir les activités/outils** qui lui paraissent importants pour changer ses habitudes de vie pour faire reculer son diabète en collaboration avec les professionnels de santé (alliance thérapeutique).

Que dois-je faire si j'accepte de participer ?

- Nous vous inviterons à **remplir des questionnaires** relatifs à votre santé durant une année et demi, avec l'aide si nécessaire de la gestionnaire en santé, **soit 3 fois sur 18 mois.**

→ Questionnaires sur la qualité de vie lié à la santé : général EQ-5D (5 questions) et lié au diabète DIAB-Q (17 questions).

→ Questionnaire lié à l'expérience dans le système de santé - questionnaire de satisfaction (9 questions)

- Nous vous inviterons à consulter votre médecin ou la gestionnaire en santé afin d'évaluer votre taux **d'hémoglobine glyquée, votre tension artérielle**, ainsi que votre **composition corporelle qui seront** évalués au début et à la fin de l'intervention à 12 mois, puis 6 mois après (3 fois sur une période de 18 mois).
- Si vous décidez de participer, vous ferez partie du groupe témoin. Ainsi vous recevrez la prise en soins habituel effectuée par votre médecin traitant. Vous pourrez bénéficier de l'autre méthode dès la fin de l'étude.

Quels sont les bénéfices et les risques liés à la participation à l'étude ?

BENEFICES

- **Votre engagement contribuera à documenter et analyser la prise en soins** du DT2 par les professionnels de santé autour de vous. La participation à l'étude n'entraîne aucun coût supplémentaire pour vous.
- Par votre participation, vous contribuez à récolter des informations essentielles pour évaluer et améliorer la qualité de **prise en soins du diabète de type 2 en cabinet de médecine de famille.**
- **Bon d'achat d'une valeur de CHF 50.- pour la participation complète** à l'étude en remplissant notamment les questionnaires.

RISQUES & CONTRAINTES

- En participant à l'étude, nous vous exposons à aucun risque ou contrainte.

RESEAU DELTA
LA SANTE INTELLIGENTE

Demande de participation à une recherche médicale

Informations détaillées

Améliorer la prise en soins et la qualité de vie des patients diabétiques de type 2 (DT2) / Value-based care in type 2 diabetes

Titre simplifié : *Alliance DT2 / Enhancing T2D Care*

Madame, Monsieur,

Nous aimerions vous présenter notre étude et vous inviter à y participer. Avant qu'une nouvelle méthode de prise en soins puisse être diffusée et proposée auprès des médecins et des autres professionnels de la santé, des recherches sont en effet nécessaires pour savoir comment cette méthode agit.

En médecine, une telle recherche s'appelle une **étude clinique**. Dans notre étude, nous voulons découvrir quel effet la méthode de prise en soins dispensée par plusieurs professionnels de la santé (médecins, infirmières et infirmiers, diététiciennes et diététiciens, spécialistes en activité physique adaptée …) et selon plusieurs approches incluant de l'éducation thérapeutique au patient a sur votre quotidien dans votre diabète de type 2 et qui nécessite un traitement. C'est pourquoi nous vous demandons si vous souhaitez participer à cette étude.

Votre participation est volontaire. Ce **formulaire d'information détaillé** doit vous aider à prendre votre décision. Vous pouvez poser toutes vos questions lors d'un **entretien avec votre médecin traitant ou les personnes en charge de l'étude que l'on nomme investigateurs ou gestionnaire de santé**. Ces derniers sont responsables du suivi des participantes et des participants de l'étude. Si vous souhaitez participer, nous vous remercions de signer la **déclaration de consentement** à la fin du document. Par votre signature, vous attestez avoir lu et compris les informations fournies. Si vous ne comprenez pas quelque chose, n'hésitez pas à demander des précisions aux investigateurs ou à votre médecin traitant.

Le formulaire d'information et de consentement se compose de trois parties :

Partie 1 **L'essentiel en bref**
Partie 2 **Informations détaillées sur l'étude**
Partie 3 **Protection des données et couverture d'assurance**

Dans la **partie 1**, vous avez un aperçu général de l'étude. Dans la **partie 2**, nous vous expliquons en détail le déroulement et le contexte de l'étude. La **partie 3** contient les informations relatives à la protection des données et à la couverture d'assurance. En signant le consentement, vous attestez que vous avez compris toutes les informations et que vous acceptez de participer.

Cette étude est initiée par le Réseau Delta. Cette institution est appelée promoteur. Le promoteur assume la responsabilité, la gestion et le financement d'une étude. La partie scientifique est effectuée en partenariat avec l'Université de St Gall, l'entreprise SOKLE partenaire technologique spécialiste dans la gestion sécurisée de données médicales. Un soutien financier est octroyé par l'assureur maladie Groupe Mutuel pour la réalisation de cette étude.

La personne de contact pour cette étude est :

Madame Cecilia Rios Valente, gestionnaire en santé, Réseau Delta

Adresse 59C route de Chancy, CH - 1213 Petit Lancy
Téléphone 079 557 88 59 (de 8h30 à 18h du lundi au vendredi)
Courriel cecilia.rios-valente@reseau-delta.ch

Partie 1 : L’essentiel en bref

1. Pourquoi menons-nous cette étude ?

Vous vivez avec un diabète de type 2 depuis moins de 10 ans et c'est pourquoi nous vous demandons si vous souhaitez participer à cette étude.

Pour le diabète de type 2, le traitement standard consiste à suivre la prise en charge proposée par votre médecin traitant, dans le but d’éviter les complications telles que l'infarctus du myocarde, un accident vasculaire cérébral ou des lésions oculaires, rénales ou nerveuses. Sans amélioration des résultats cliniques mesurés notamment lors de prises de sang, une utilisation au long court d’injection d’insuline quotidienne deviendrait alors nécessaire.

Dans cette étude, nous examinons comment une nouvelle méthode de prise en soins du diabète de type 2 dans son quotidien au travers de prise de mesure clinique et par le biais de questionnaire. Nous allons évaluer l’efficacité au niveau de la maladie, la qualité de vie et également sous l’angle économique. Une nouvelle approche pour la prise en soins permettrait d’optimiser votre traitement afin de tendre vers la guérison de votre diabète pour cela il nous faut d’abord mesurer la pratique actuelle avec le point de vue du patient (questionnaire) . La rémission du diabète de type 2 est possible.

Vous en apprendrez davantage sur le contexte scientifique de l’étude au **chapitre 4**.

2. Que devez-vous faire si vous participez ?

Votre participation durera 18 mois. Vous serez sollicité par notre gestionnaire en santé dans le cadre de l’étude afin de prendre certaines mesures clinique et remplir des questionnaires courts. En parallèle vous poursuivrez vos traitements standards (consultation) avec votre médecin traitant.

Un rendez-vous avec notre gestionnaire en santé dure en moyenne moins de 20 minutes. Les rendez-vous sont détaillés dans **la figure au chapitre 5**.

Si vous décidez de participer, la prise en soins reste identique à celle que vous avez actuellement.

Vous en apprendrez plus sur le déroulement et les procédures de l’étude au **chapitre 5**.

3. Quels sont les bénéfices et les risques liés à la participation ?

Bénéfices

Cette étude a pour but d'améliorer la prise en soins du diabète de type 2 pour tendre vers la guérison. Il convient de souligner que des études scientifiques internationales ont déjà démontrés un bénéfice direct sur des patients comme vous (amélioration des valeurs de laboratoire, augmentation de l'activité physique, perte de poids, guérison du diabète, etc.) grâce aux études scientifiques. Votre participation peut dans tous les cas contribuer à aider de futurs patients diabétique. Vous n'allez peut-être toutefois pas être directement guéri de votre diabète à la suite de votre participation à cette étude mais vous allez contribuer à faire avancer les connaissances et la science.

Risques

Les risques liés à votre participation à l'étude sont minimes pour ne pas dire inexistants, étant donné que vous continuez à être suivi par votre médecin traitant et que l'étude n'interféra pas avec vos traitements médicamenteux (excepté s'il devient inutile ou le dosage doit être abaissé car vos données de laboratoire sont améliorées au niveau du diabète).

Des effets indésirables peuvent survenir si vous choisissez de ne pas respecter les indications de votre médecin traitant ou tout autres professionnel de la santé responsables. Ceci ne sera pas directement lié à la présente étude.

Jusqu'à présent, aucun effet indésirable n'a été répertorié.

Vous trouverez davantage d'informations sur les éventuelles contraintes au **chapitre 6**.

Partie 2 : Informations détaillées sur l'étude

4. Contexte scientifique

4.1 Contexte : pourquoi menons-nous cette étude ?

Le diabète de type 2 (DT2) est une maladie chronique fréquente. Lorsque l'on est atteint de DT2, si aucun changement des habitudes de vie comme le comportement au niveau alimentaire ou au niveau de l'activité physique n'est entrepris, la maladie s'aggrave progressivement et nécessite parfois la prise d'insuline sous forme d'injection quotidienne à long terme. Le traitement et la gestion du diabète dépendent donc d'une multitude de paramètres, liés notamment à son propre mode de vie.

Le diabète est généralement traité avec des médicaments et éventuellement des séances avec une personne spécialisée en diététique et/ou spécialisée en physiothérapie.

Il existe déjà un nombre important de recherches concernant la prise en charge du diabète de type 2. Les études menées jusqu'à présent notamment en Suisse, principalement en milieu hospitalier, ont montré qu'une prise en charge interdisciplinaire c'est-à-dire en collaboration avec des professionnels de la santé de diverses spécialités, avec l'éducation thérapeutique au patient, est nécessaire pour une prise en charge optimale d'un patient diabétique. En effet, le diabète doit être considéré comme un dérèglement chronique et complexe du fonctionnement de notre organisme dans la gestion du sucre et pour lequel un patient peut avec une aide adéquate (alliance thérapeutique) rétablir l'équilibre perdu, lorsque le diagnostic est établi suffisamment précocement. Ainsi, nous savons donc que sous certaines conditions, il est possible de guérir de son diabète de type 2.

Nous examinons donc dans cette étude si une prise en soins interdisciplinaire accompagnée d'éducation thérapeutique au patient est efficace pour améliorer les paramètres cliniques tels que l'hémoglobine glyquée et la composition corporelle (part de graisse, de muscle et d'os) ainsi que la qualité de vie liée à la santé par rapport à un traitement standard. Et finalement, pouvoir observer l'impact économique de cette prise en soins dans un but de la rendre accessible à tous.

4.2 Structure de l'étude : comment procédons-nous ?

Dans notre étude, les participants selon leurs médecins traitants sont répartis au hasard (affectation aléatoire) dans des groupes, c'est ce qu'on appelle la randomisation. Cette méthode est importante pour obtenir des résultats fiables.

4.3 Réglementation de la recherche scientifique impliquant des êtres humains

Nous réalisons cette étude conformément aux lois en vigueur en Suisse (loi relative à la recherche sur l'être humain, lois sur la protection des données). En outre, nous respectons toutes les directives reconnues au niveau international. La commission d'éthique compétente a examiné et autorisé l'étude.

Notre étude est effectuée dans la Canton de Genève. Il y a plusieurs centaines de participants affiliés au Réseau Delta à Genève qui sont sollicités.

Vous trouverez également une description de cette étude sur le site Internet de l'Office fédéral de la santé publique, à l'adresse www.kofam.ch, sous le numéro d'enregistrement SNCTP XXX ou le numéro BASEC XXX.

5. Déroulement de l'étude

5.1 Que devez-vous faire si vous participez à l'étude ?

La participation à l'étude est volontaire et dure 18 mois. Vous devez respecter le calendrier des rendez-vous (→ chapitre 5.2) ainsi que toutes les consignes données par l'équipe de recherche et votre médecin traitant.

Vous devez informer l'équipe de recherche ou directement votre médecin traitant

- si votre état de santé évolue, par exemple si vous vous sentez moins bien ou si vous présentez de nouveaux troubles ; vous devez continuer de l'en informer si vous vous retirez de l'étude (→ chapitres 5.3 et 5.4) ;
- Pour les femmes : si vous envisagez une grossesse ou êtes enceinte (risque de diabète gestationnel)

5.2 Que se passe-t-il lors des rendez-vous ?

Les rendez-vous font partie de votre prise en charge standard de suivi de diabète. En marge de vos rendez-vous de consultation médicales, la gestionnaire en santé (GS) vous sollicitera pour la prise de vos mesures clinique et au sujet des questionnaires courts.

En effet, dans le cadre de l'étude, vous serez sollicités pour répondre à des questionnaires et effectué de mesures cliniques, une année après avoir répondu aux premier questionnaire puis 6 mois après (durée totale 18 mois). Les questionnaires peuvent être remplis sur place ou envoyé par voie électronique (possibilité par téléphone si nécessaire avec notre gestionnaire en santé) selon votre choix personnel. Certaines mesures cliniques (notamment hémoglobine glyquée, tension artérielle, composition corporelle) seront prises au cabinet par la GS ou déléguées à d'autres professionnels de la santé.

Voici ce que nous faisons à tous les rendez-vous (avec votre médecin traitant, un spécialiste en éducation thérapeutique au patient ou la gestionnaire en santé):

- Nous répondons à vos questions.
- Nous vous posons des questions sur votre état de santé et votre qualité de vie.
- Nous mesurons des paramètres cliniques de routine
- Nous vous aidons à remplir des questionnaires ou les mettons à votre disposition.

Lors de certains rendez-vous, nous faisons en outre :

- Prélèvement d'une goutte de sang obtenue par piqûre au bout du doigt pour l'hémoglobine glyquée
- Prise de la tension artérielle
- Mesure de la composition corporelle et indice de masse corporelle
- Questionnaire sur la qualité de vie lié à la santé :
  - Questionnaire générique lié à la qualité de vie EQ5D (5 questions)
  - Questionnaire spécifique lié au diabète DIAB-Q (17 questions)
- Questionnaires lié à l'expérience dans le système de santé

  Questionnaire PREM - questionnaire de satisfaction (9 questions)

Ces examens/questionnaires nous permettent d'évaluer l'efficacité de la méthode d'intervention.

Les questionnaires sont sous forme électronique (à privilégier). Sur demande, le format papier est disponible.

En résumé le déroulement de votre participation à l'étude s'articule comme suit (Figure 1):

1. Convocation par la gestionnaire en santé ou votre médecin traitant pour effectuer les mesures cliniques (hémoglobine glyquée, composition corporelle et tension artérielle) et envoi des questionnaires courts à remplir (EQ5D et DIAB-Q).
2. Une année (12 mois) après la prise des mesures cliniques et le remplissage des questionnaires courts, vous êtes à nouveau invité par la gestionnaire en santé à remplir les questionnaire d'évaluation (EQ5D et DIAB-Q) ainsi que celui relatif à votre expérience, nommé PREM (satisfaction). Vous êtes également convoqué afin d'effectuer les mesures cliniques. En parallèle cous continuer de suivre votre traitement habituel et continuer de suivre les consultations avec votre médecin traitant.
3. Six mois plus tard (soit à 18 mois après votre première convocation), vous êtes à nouveau sollicité par la gestionnaire en santé afin de remplir les questionnaires d'évaluation (EQ5D et DIAB-Q) ainsi que celui relatif à votre expérience, PREM. Vous êtes également convoqué afin d'effectuer les mesures cliniques.

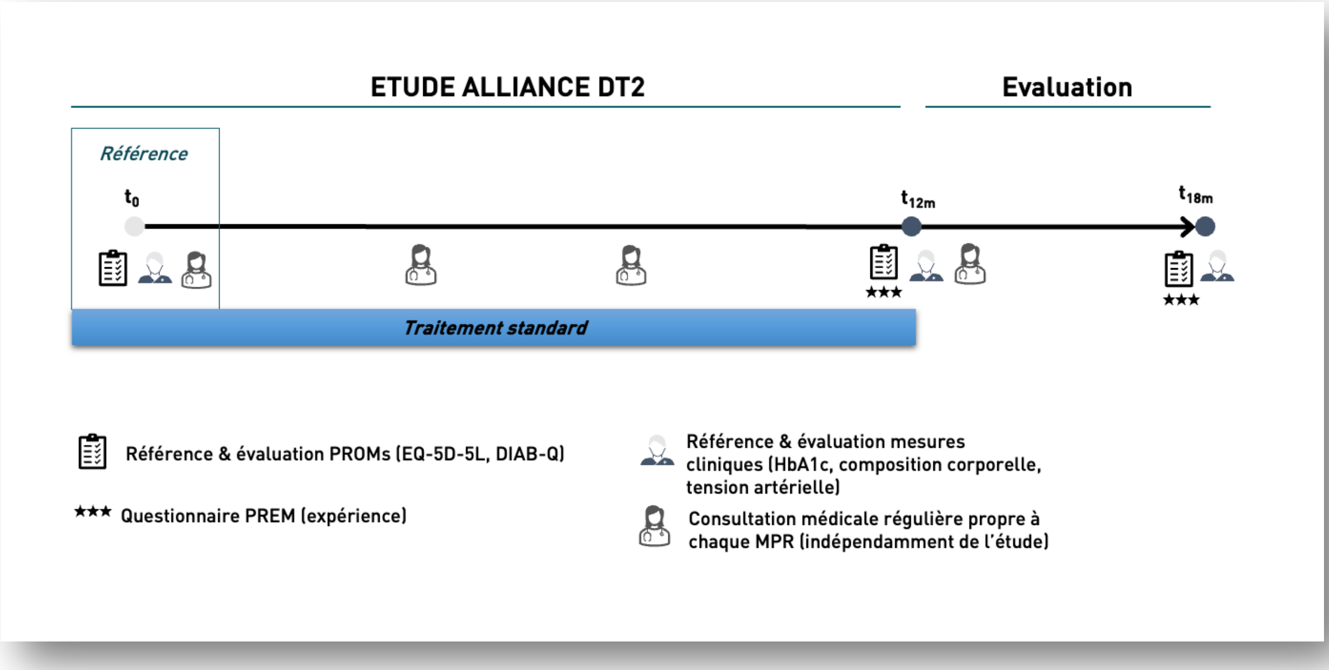

Figure 1 Déroulement de l'étude pour le groupe de patient "contrôle"

Vous recevrez un récapitulatif précis des rendez-vous liés à l'étude par le biais de la gestionnaire en santé. Merci de noter qu'il n'est en principe pas possible de reporter un rendez-vous. Si vous deviez malgré tout déplacer un rendez-vous pour des raisons importantes, nous vous prions de nous en informer rapidement.

5.3 Quelles données sont traitées dans le cadre de l'étude ?

Dans le cadre de l'étude, vous autorisez le Réseau Delta via SOKLE à partager votre numéro d'assurance codé avec votre assurance-maladie. Pour les assurés du Groupe Mutuel cela permet au Groupe Mutuel d'identifier les données de facturation des participants respectifs. Les données de facturation (liées notamment à la médication) sont ensuite renvoyées au Réseau Delta qui les associe au code correspondant par l'intermédiaire de SOKLE pour les analyses de l'étude.

5.4 Quand la participation à l'étude prend-elle fin ?

Votre participation dure 18 mois (après avoir complété les derniers questionnaires et prise de mesures cliniques). Vous pouvez interrompre votre participation à tout moment avant cette date (→ chapitre 5.4). Vous n'avez pas besoin de vous justifier. Si vous souhaitez mettre fin à votre participation, veuillez en informer la gestionnaire en santé qui le rapportera à l'investigatrice principale.

Si vous vous retirez de l'étude, cela n'aura aucune incidence sur vos soins médicaux et votre traitement (→ chapitre 5.4 pour les autres possibilités de traitement).

Si vous vous retirez de l'étude, nous pourrons encore analyser les données. Vos données d'étude restent codées (→ chapitre 9).

5.5 Que se passe-t-il si vous ne souhaitez pas participer ?

Si vous ne participez pas à cette étude, votre traitement et votre prise en charge médicale seront assurés conformément aux standards actuels.

5.6 Grossesse

La grossesse engendre des changement physiologiques qui perturbe vos résultats cliniques d'analyse (hémoglobine glyquée, composition corporelle, etc…). Ainsi, vous ne pouvez donc pas avoir d'enfants pendant votre participation à l'étude. Cela vaut pour les personnes concernées qui participent à cette étude. Vous pouvez discuterez de ces questions avec votre médecin traitant ou la personne investigatrice de contact.

Pour les personnes concernées qui veulent débuter une grossesse

Vous devez éviter de débuter une grossesse pendant votre participation à l'étude (risque de diabète gestationnel). Vous devez informer votre partenaire / vos partenaires que vous prenez part à cette étude. Si vous allaitez ou êtes enceinte, vous ne pourrez pas participer à l'étude.

6. Risques, contraintes et effets indésirables

6.1 Quels sont les risques et les contraintes liés à l'étude ?

La participation à cette étude ne comporte pas de risque et des contraintes directes mais indirecte uniquement, comme tout traitement médical d'une maladie.

Si vous prenez actuellement des médicaments, nous vous prions de vous référez aux indications de votre médecin traitant ou votre pharmacien. L'information officielle du médicament (notice d'emballage) est directement accessible sur le site internet suivant : https://www.swissmedicinfo.ch/?Lang=FR .

6.2 Risques et contraintes liés aux examens dans le cadre de l'étude

Nous effectuons différents examens d'ordre médical pour cette étude (→ chapitre 5.2). Ces examens sont des procédures éprouvées relatives à un traitement standard par votre médecin traitant. Néanmoins, ils peuvent comporter des risques et des contraintes, c'est-à-dire qu'ils peuvent être désagréables ou avoir des effets indésirables. Dans cette étude, le risque/contrainte est le suivant :

- Piqûre au bout du doigt/prise de sang : des hématomes (lors d'une prise de sang), des saignements ou des gonflements peuvent survenir au point de piqûre/ponction.

7. Financement et indemnisation

Cette étude est initiée par le Réseau Delta et est soutenue financièrement pour le Groupe Mutuel assurance. L'Université de St Gall est l'institut de recherche indépendant mandaté pour les analyses (partenaire scientifique). Le partenaire technologique est SOKLE, une société d'informatique genevoise spécialisée dans les logiciels médicaux. Pour ses assurés, le Groupe mutuel met à disposition pour l'étude les données de facturation sous forme codées. Pour les assurés d'autres caisses, l'accès aux données s'effectue selon les possibilités contractuelles.

Les chercheurs participant à l'étude n'en retirent aucun avantage financier.

Si vous participez à cette étude, vous recevrez directement sous forme d'un bon d'achat de CHF 50.- pour les 3 rendez-vous (questionnaires et mesures métaboliques cliniques) sur la période de 18 mois de l'étude.

La participation à l'étude n'entraîne aucun coût supplémentaire pour vous ni pour votre assurance-maladie.

8. Résultats de l'étude

Les résultats qui vous concernent personnellement vous sont communiqués par votre médecin traitant ou la gestionnaire en santé.

En plus des résultats individuels, l'étude produira des résultats globaux qui proviennent des données de toutes les personnes y participant. Il s'agirait p.ex. de nouvelles connaissances sur les facteurs important/e/s pour guérir du diabète de type 2 (→ chapitre 4.1). Ces résultats ne vous concernent pas nécessairement directement. Si vous le souhaitez, sur demande, les investigateurs vous transmettront un résumé des résultats globaux à la fin de l'étude.

Partie 3 : Protection des données et couverture d’assurance

9. Protection des données et des échantillons

Nous protégeons vos données (p. ex. les données médicales telles que l’hémoglobine glyquée, la tension artérielle et la composition corporelle). Les lois suisses prévoient des règles strictes en matière de protection des données.

La législation suisse sur la protection des données vous donne le droit d'accéder, de rectifier et de recevoir les données qui ont été collectées, traitées et transmises dans le cadre de l'étude. Dans des cas exceptionnels en raison d'autres exigences légales ou réglementaires, ces droits ne peuvent pas toujours être garantis. Si vous avez des questions à ce sujet, veuillez contacter la gestionnaire en santé du Réseau Delta qui vous renseignera.

9.1 Codage des données et des échantillons

Toute étude génère des données issues des examens (p. ex. l’hémoglobine glyquée, la tension artérielle et la composition corporelle). Ces données sont enregistrées de manière codée, sous forme électronique. Le codage signifie que les informations personnelles qui peuvent vous identifier directement sont conservées *séparément* des autres données, sous la forme d’une liste (liste d’identification) qui identifie chaque personne avec un code unique. Vos données mesurées restent accessibles à votre médecin traitant (logiciel médical). Ainsi, votre nom, votre date de naissance ou votre adresse *ne figurent pas* directement avec les autres données collectées. Cette liste d’identification reste pendant 10 années auprès du Réseau Delta et est ensuite totalement anonymisée (nécessité d’un code d’accès utilisateur spécifique). Personne d’autre ne la reçoit. Les exceptions particulières sont réglées au chapitre 9.5.

Lorsque nous transmettons des données dans le cadre de cette étude à l’institut de recherche, elles sont toujours codées et vos données personnelles sont protégées. Il en va de même lorsque la liste des assurés est transmise pour le couplage des données de facturation avec votre assureur maladie partenaire du Réseau Delta (codification).

9.2 Sécurité des données et des échantillons pendant l'étude

Le promoteur Réseau Delta ainsi que la société SOKLE sont responsables de la sécurité de vos données de cette étude. Ils veillent au respect des lois en vigueur, par exemple des lois sur la protection des données. Voici comment le promoteur de l'étude protège contractuellement vos données :

- Convention Réseau Delta & SOKLE
- Convention Réseau Delta & Groupe Mutuel, et annexe
- Convention SOKLE & Groupe Mutuel
- Convention Université de St Gall & Groupe Mutuel
- Convention pour le transfert des données Réseau Delta & Université de St Gall

Dans cette étude, vos données sont saisies et transmises par voie électronique. Les données sont stockées sur un serveur situé à Onex (Genève, Suisse) dans un centre de données du Réseau Delta. Seul un administrateur, qui a signé la charte de confidentialité pour l'accès aux données, a accès au serveur physique pour la maintenance et la surveillance. L'administrateur doit être muni d'une carte d'identité et l'accès est enregistré. Toutes les données sont sauvegardées chaque jour. Au bout d'un mois, les sauvegardes précédentes sont déplacées sur un disque externe. Néanmoins, le risque que des personnes non autorisées accèdent à vos données personnelles ne peut être entièrement exclu (p. ex. risque de « piratage informatique »).

Il peut être important que votre médecin traitant partage des données médicales (valeur clinique, comorbidité soumises à déclaration) avec l'équipe de recherche et des données codées avec votre assureur maladie partenaire, avec votre autorisation. Cela vaut également pour les éventuels autres médecins qui vous suivent. En signant le consentement, vous autorisez la communication de ces données, si nécessaire.

9.3 Sécurité des données après la fin de l'étude

Lorsque l'étude est terminée, le promoteur continue d'assurer la sécurité de vos données. La loi prescrit que tous les documents de l'étude, par exemple les formulaires de collecte de données, doivent être conservés pendant au moins 10 ans.

Au terme de cette longue période, les données de l'étude restent codées (ou, le cas échéant, sont entièrement supprimées pour l'institut de recherche). Les données relatives à la santé de votre dossier médical, y compris celles de cette étude, sont et resteront toujours accessibles à vos professionnel de la santé. Afin d'avoir accès aux données, il est nécessaire d'avoir un accès utilisateur. Ceci exclue l'utilisation/consultation par des tiers.

Une fois l'étude terminée, les résultats de l'étude sont généralement publiés dans des revues scientifiques. Pour ce faire, les données sont si nécessaires envoyées sous forme anonymisée à d'autres spécialistes afin qu'ils puissent réviser la publication. Ces données ne peuvent pas être réutilisées à des fins de recherche par des tiers.

9.4 Droit de consultation lors des contrôles

La réalisation de cette étude peut faire l'objet de contrôles par la commission d'éthique compétente. Le promoteur doit également procéder à des vérifications afin de garantir la qualité de l'étude et de ses résultats. Pour ces contrôles, un petit nombre de personnes spécialement formées ont accès à vos

données personnelles et à votre dossier médical. Dans ce cadre, les données ne sont donc *pas* codées. Les personnes qui consultent vos données non codées sont soumises au secret professionnel.

En tant que participant·e, vous avez à tout moment le droit de consulter vos données.

10. Couverture d'assurance

Vous bénéficiez d'une couverture d'assurance si vous subissez un dommage du fait de l'étude – c'est-à-dire du fait de la méthode de prise en soins. La procédure est réglée par la loi. Si vous pensez avoir subi un dommage du fait de l'étude, veuillez-vous adresser directement à votre médecin traitant ou à la gestionnaire en santé ou à votre assurance privée.

Si un dommage résulte de l'utilisation conforme de l'application d'un traitement conventionnel, les règles en matière de responsabilité sont les mêmes que pour les traitements en dehors d'une étude. En pareil cas, l'assurance responsabilité civile prend en charge les frais.

Déclaration de consentement pour la participation à l'étude « Améliorer la prise en soins et la qualité de vie des patients diabétiques de type 2 (Alliance DT2) » / *Value-based care in type 2 diabetes (Enhancing T2D Care) »*

Veuillez lire attentivement ce formulaire. N'hésitez pas à nous poser des questions si vous ne comprenez pas quelque chose ou si vous souhaitez des précisions. Votre consentement écrit est nécessaire pour participer.

**Numéro BASEC**	
**Titre de l'étude**	Améliorer la prise en soins et la qualité de vie des patients diabétiques de type 2 / *Value-based care in type 2 diabetes*
**Titre simplifié**	Alliance DT2 / *Enhancing T2D Care*
**Institution responsable**<br>(promoteur et adresse)	Réseau de soins Delta SA<br>Dr Philippe Schaller, MD, FMH, MPH<br>98 route de Chancy<br>CH - 1213 Onex
**Lieu de réalisation**	Canton de Genève
**Investigatrice-principale**<br>**Investigatrice-coordinatrice**<br>**Gestionnaire en santé**	Dre Minette-Joëlle Zeukeng, PharmD, FPH, PhD<br>Mme Joëlle Coclet, PhD<br>Mme Cecilia Rios Valente
**Participant**<br>Nom et prénom en caractères d'imprimerie :<br>Date de naissance :	

Attestation du médecin traitant/gestionnaire en santé : Par la présente, j'atteste avoir expliqué au participant la nature, l'importance et la portée de l'étude. Je déclare satisfaire à toutes les obligations en relation avec cette étude selon le droit suisse. Si je devais prendre connaissance, au cours de l'étude, d'éléments susceptibles d'influer sur la disposition du participant à prendre part à l'étude, je m'engage à l'en informer immédiatement.

Lieu, date	Nom et prénom du **médecin traitant/gestionnaire en santé** en caractères d'imprimerie<br><br>Signature

- J’ai reçu des informations orales et écrites sur l’étude (but, déroulement, bénéfices & risques) de la part de mon médecin traitant traitant/investigatrice-coordinatrice/gestionnaire en santé soussigné.e.

- Je participe volontairement à l’étude.

- J’ai eu suffisamment de temps pour prendre ma décision. Je conserve les informations écrites et je reçois une copie de ma déclaration de consentement écrite.

- Je peux mettre fin à ma participation à tout moment, je n’ai pas besoin de me justifier. Même si je me retire de l’étude, ma prise en charge médicale reste assurée. Les données collectées jusque-là restent enregistrés et seront encore analysés dans le cadre de l’étude.

- J’ai compris que ma participation à l’étude implique le traitement de mes données personnelles dans les conditions décrites dans la notice d’information.

- J’ai compris que mes données ne sont transmises pour cette étude que sous forme codée et pseudanonymisée. Le promoteur veille au respect de la protection des données conformément aux normes suisses.

- Mon médecin traitant est informé de ma participation à l’étude. Il peut partager avec les investigatrices ou la gestionnaire en santé certaines des données de mon dossier médical qui sont importantes pour l’étude. Cela vaut également pour les autres médecins qui me suivent.

- Les spécialistes compétents du projet et la commission d’éthique peuvent consulter mes données non codées à des fins de contrôle. Toutes ces personnes sont soumises au secret professionnel.

- L’assurance responsabilité civile de l’institution Réseau Delta couvre les dommages éventuels.

Lieu, date	Nom et prénom du participant en caractères d’imprimerie<br><br>Signature du participant
