# Supplementary material for: Enhancing type 2 diabetes care by an individualized and group-based therapeutic patient education program: study protocol for a cluster randomized trial
Source: Trials. 2025 Dec 23;26:577. doi: 10.1186/s13063-025-09302-x (PMC12723846; doi:10.1186/s13063-025-09302-x)
Supplement: Supplementary file 3 — Additional file 3: Appendix C: Monitoring Plan. Additional file 4: Appendix D: Ethics Committee approval [file 13063_2025_9302_MOESM3_ESM.pdf]

Monitoring plan

Form based on the monitoring platform of the Swiss Clinical Trial Organisation (SCTO). www.sctoplatforms.ch.

MONITORING PLAN - Alliance DT2

**Study details**	
**Study title**	Alliance DT2
Type of study / Risk category (according to Swiss law)	ClinO_Other / A
Name of operation under investigation	Implementation of an interdisciplinary program (therapeutic patient education and PROM/PREM questionnaires) for the treatment of type 2 diabetes.
CRF system (if applicable)	Specify if neither secuTrial nor REDCap: SOKLE/electronics
**Developer**	Delta care network
Main contact person	Dr Philippe Schaller, MD, FMH, MPH<br>Managing Director
Address	98 route de Chancy<br>CH - 1213 Onex
Telephone number, e-mail	079 652 51 16, http://www.philippe-schaller.ch
**Principal investigator**	Dr. Minette-Joëlle Zeukeng, PharmD, PhD, FPH<br>079 892 19 81<br>minette.zeukeng@reseau-delta.ch
**Contact person for data management (monitor)**	Cécilia Rios Valente<br>Cecilia.rios-valente@reseau-delta.ch
**Contact person for operational management (coordinator)**	Joëlle Coclet, PhD<br>Joelle.coclet@reseau-delta.ch

Monitoring plan approval details		
Version / Date of monitoring plan	1	11.10.2024
**Author of monitoring plan**		
First and last name	Minette-Joëlle Zeukeng	
Function	Principal investigator	
Date / Signature	11.10.2024	
**Developer**		
First and last name	Philippe Schaller	
Date / Signature	11.10.2024 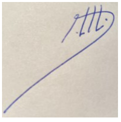	

Table of contents

1. Introduction....4
2. Risk-Based Monitoring (RBM) strategy....5
   2.1 Strategy (table 1)....5
   2.2 Visit schedule (table 2)....5
   2.3 Monitoring activities (table 3)....6
3. Responsibilities....7
   3.1 Monitor....7
   3.2 Developer....7
   3.3 Investigators....7
4. Follow-up activities....7
   4.1 Informed consent process and documentation....7
   4.2 Source data verification (SDV)....8
   4.3 Security....8
   4.4 Randomization....8
   4.5 Protocol compliance....8
   4.6 Confirmation of inspection visits....8
5. Site start-up visit (cabinet)....8
6. Routine check-up....9
7. Central data control....9
8. Closing visit....9
9. Spreads....10

Acronyms and abbreviations....11

Appendices....12

1. Introduction

Monitoring activities are carried out in accordance with GCP standards. This monitoring plan is based on the study's specific requirements. If agreed commitments change after the start of the study, a new version of the monitoring plan may be issued.

Standard operating procedures (SOPs) are detailed in Appendix 2 of the Alliance DT2 research protocol.

The Alliance DT2 clinical trial is taking place in several practices affiliated to the Delta Network, making it multicentric in nature. However, there are a number of reasons for adopting a monitoring plan similar to that of a single-center trial, given the specific organizational structure and centralized coordination under the responsibility of the Delta Network.

All the practices involved in this clinical study belong to Réseau Delta, which operates as an umbrella organization. Primary care physicians (PCPs) operate under uniform guidelines, and coordination is handled centrally by the promoter (*Réseau de soins Delta SA*). This structure guarantees standardization of practices within the various practices. We are not talking about independent sites, but a coordinated network sharing the same procedures, training and responsibilities, similar to a single center with several sub-sites.

The Delta Network centralizes the management and coordination of the various stages of the study:

- Patient inclusion and consent are managed via a single platform (*SOKLE*), under the direct supervision of the Health Manager (GS).
- The GS and the coordination team are responsible for the proactive follow-up of participants, the collection of clinical data (HbA1c, PROM/PREM questionnaires), and the organization of Quality Circles in the various practices. This centralization of administrative and clinical tasks under a single structure (Delta Network) brings the organization closer to a monocentric management model where a single team controls operations in several physical locations.

The central intervention of this study, the Physician-Patient Quality Circles, is organized in a similar way in each practice. The educational procedures, the conduct of the interventions and the collection of clinical measures are standardized according to a single plan applied to all participating sites.

- GS coordinates physician training and ensures uniform application of therapeutic patient education (TPE) protocols.
- The materials used (metabolic measurements, questionnaires) are also standardized and administered in the same way, guaranteeing uniformity of practice, as in a monocentric setting.

The monitoring approach in this study will be centralized and standardized. Although (remote) monitoring visits will take place at several practices, data will be pooled in a single system (SOKLE platform) and monitoring will be carried out in a similar way at each site, with periodic checks :

- Data checks (SDV) and discrepancy management will be carried out from the same platform, under the supervision of the GS and the principal investigator.

- Centralized monitoring of the data collected (metabolic measurements including HbA1c, and PROM/PREM questionnaires) will take place at predefined points (10% of participants included - see details in Appendix 2) independently of site, as in a monocentric setting where data would be centralized in a single database.

The frequency and content of monitoring visits will be standardized, with additional visits only when necessary (e.g. major non-conformity or problem identified during the first routine visit). This approach, where all practices receive uniformly scheduled visits, supports the idea of a monitoring model similar to that of a single center.

Finally, the fact that the health manager and the intervention team working with all participating practices are covered by a single insurance policy under the Delta Network umbrella reinforces this idea of centralized management. This reduces the variability of practices and risks across different practices, as they are all governed by the same type of contractual obligations. This uniformity reduces the need for a more detailed monitoring plan for each practice, as would be required in a traditional multicenter trial with several totally independent sponsors or institutions.

2. Risk-Based Monitoring (RBM) strategy

For details, see the RBM risk score calculator report (Appendix 4).

2.1 Strategy (table 1)

Recommended risk-based monitoring strategy according to the RBM score calculator of the SCTO[1]	low-risk
Medium or high risk factors according to the CTSS RBM score calculator[1]	Withdrawal/abandonment<br>Volume/complexity<br>Qualité du formulaire de rapport de cas (CRF)
Central data control?	Yes

2.2 Visit schedule (table 2)

Remote site opening visit (e-mail to the office)	Before inclusion of the first participant
Routine inspection (Visit 1)	If necessary, to identify any non-conformities, logistical problems or outliers.
Routine inspection (Visits 2 to 6)	If necessary, in the event of non-conformity or problems serious adverse events.
Remote closing visit	When all participants have completed their final visits and data collection is complete<br>completed.
Central data control	When creating a new Doctor-Patient Quality Circle using SOKLE software.

During the course of the study, the extent of data verification and the number of routine monitoring visits may be re-evaluated according to the results. The controller (member of the investigating team) must inform the sponsor if additional time or resources are required.

2.3 Monitoring activities (table 3)

**Action**	**Scope of study** (% of participants)	**Comments**
Verification of informed consent	100%	
Complete SDV	100%	Metabolic data (*HbA1c level)* per quarter for one year, then at 6 months for a total of 18 months
Partial SDV of<br>key data, including<br>including visit<br>schedule	20%	Metabolic data (*body composition,*<br>*blood pressure)*<br>*Verification possible from measurement device source files (date and time)*
Partial SDV of key data, including visit schedule	10%	Reference and evaluation PROMs (EQ5D, DIABQ)<br>Intervention PROMs (PHQ-9, DIAB-Q)Per quarter<br>PREM (patient experience)

See the VDS table in Appendix 2 for details of the VDS scope.

3. Responsibilities

3.1 Monitor

The controller's responsibilities are detailed in the GCP.

Compliance with protocol requirements is important to ensure the ethical conduct of the clinical study, as well as the scientific validity, accuracy and completeness of the data generated during the clinical study. Monitoring ensures that these requirements are met.

First and foremost, the instructor must be trained by the promoter:

- Any documentation relating to the study, e.g. protocol or (e)CRF
- Any specific control requirements, such as source data verification (SDV).
- Regulations and guidelines in current version: Ordinance on clinical trials in human research (ClinO) chapter 4

3.2 Developer

The sponsor is responsible for setting up and monitoring the study. The sponsor's responsibilities are detailed in the GCP.

All study documents (TMF - Trial master file) are centralized and available on request. The sponsor is also responsible for study-specific training.

Training must cover at least the following points

- Medical and terminal training
- All study-specific documents
- Study design and procedures
- Study schedule
- Logistics (organizing sessions, supplying equipment for metabolic measurements, etc.)
- Composition, completion and recording of the CRF (in the case of a paper version)
- Query processing / Estimated database lock times
- Study-specific quality systems / SOPs

3.3 Investigators

The principal investigator' s responsibilities are specified in the GCP.

As far as control is concerned, the investigating team is responsible for facilitating control activities, making them accessible for control and being available during control visits.

4. Follow-up activities

4.1 Informed consent process and documentation

The controller (investigating team) must check that :

- The consent process is appropriate, complies with international guidelines for good clinical practice (GCP and Swiss national regulations) and is documented in the participant's file.
- Only current versions and the latest versions approved by the Ethics Commission of the Participant Information and Consent Form are used.
- The consent form has been signed/dated by the participant and by the qualified person authorized by the principal investigator in the registry.

- Consent was obtained and properly documented for all participants who underwent an intervention as part of the study, in accordance with the randomization register.
- The original consent form is kept by the health manager (paper and electronic) in SOKLE. A copy of the fully signed document is given to the participant.
- Consent was obtained in an appropriate manner prior to the implementation of any study-related procedure.
- Participant inclusion, randomization and identification registers are correctly completed and updated.

4.2 Data verification at source (SDV)

The controller checks the CRF data against the corresponding source data, in accordance with Table 2.

The controller is a member of the investigative team.

Any discrepancies will be discussed with the investigators and, if necessary, arrangements will be made.

4.3 Security

In accordance with Table 2, the monitor (health manager) verifies that serious adverse events (AEs) :

- are correctly recorded in accordance with the source documents and are consistent with concomitant medications and illnesses
- are appropriately flagged and monitored according to protocol and regulatory requirements

4.4 Randomization

The monitor must compare the randomization list with the nature of the treatments administered to the participants in accordance with Table 2.

4.5 Protocol compliance

The monitor will assess protocol compliance at each visit (e.g. timing of visits) in accordance with Table 3 and record any deviations in SOKLE.

4.6 Confirmation of inspection visits

All remote visits will be preceded by a confirmation e-mail or letter containing all the necessary information concerning the visit (date, time, place of visit, documents and basic data requested, participants, etc.).

5. Site start-up visit (cabinet)

The site will be initiated in accordance with

Table 2. The controller will perform the

following activities:

- Reminder of PCM principles and regulatory requirements
- Training on key elements of the protocol (information) and study procedures.
- Training on recruitment and informed consent processes
- Discuss the roles and responsibilities of the study team

- Identification of source documents (metabolic measurement devices and SOKLE)

- Where applicable, ensure receipt of required forms (see SOP, Appendix 2 of Alliance protocol DT2)

6. Routine check-up

Routine inspection visits will be carried out in accordance with Table 2.

The controller will perform the following activities:

- Verify that the informed consent process is carried out correctly
- Check that study tasks are carried out by delegated personnel
- Check compliance with eligibility criteria
- Check that the protocol is being followed and that any deviations are properly reported to the sponsor (recorded in SOKLE).
- Perform source data verification (SDV) on the CRF as specified in Appendix 2.
- Identify, discuss and resolve action points with the firm's staff.
- If necessary, review registrations and discuss options for increasing participant recruitment.
- Where applicable, check randomization procedures in accordance with Appendix 2, and ensure that blinding is maintained.
- If necessary, gather documents for the promoter (CRF) After the

visit, the inspector will document any shortcomings in SOKLE.

7. Central data control

Device source data for metabolic measurements are available and accessible (.xlsx or .csv export) directly from the devices. Checks can therefore be made using the time and date of measurement contained in the extractions. The manager (monitor) transcribes the data directly into SOKLE during visits.

At SOKLE level, all actions (backups) can be tracked and notified to the SOKLE administrator on request.

8. Closing visit

A closing visit will be carried out remotely, in accordance with Table 2. The

controller will perform the following activities:

- Resolve outstanding action points with site staff.
- Ensure that all forms and registers are completed and signed by the principal investigator.
- Inform interviewers about :
    - Responsibility for serious adverse events, as specified in the protocol
    - Archiving and retention requirements for study documents

After the visit, the inspector will, if necessary, draw up a visit report to be entered into SOKLE.

G. Spreads

A protocol or PCG deviation is any failure to comply with the protocol or regulatory requirements. Where applicable, protocol deviations should be reported in the monitoring report and discussed with the principal investigator.

All deviations must be reported by the observer and recorded in SOKLE.

Any potential critical deviations observed during a control visit or independently of the visits must be reported by the controller to the promoter before the control visit report is drawn up.

Sample questions:

- The informed consent process was not carried out properly
- Violation of inclusion/exclusion criteria
- Assigning a participant to the wrong study group
- SAE reporting requirements not met
- Visits and follow-up of participants not carried out in accordance with protocol
- Any breach of the GCP

Acronyms and abbreviations

ClinO	Ordinance on clinical trials, with the exception of clinical trials on devices medical
VOCS	Closing visit
(e) CRF	Electronic case report form
CE	Ethics Committee
GCP	Good clinical practice
HRA	Human Research Act
ICF	Informed consent form
PI	Principal investigator
RBM	Risk-based monitoring
RMV	Routine check-up
SAE	Serious adverse event
SCTO	Swiss Clinical Trials Organization
SDV	Checking source data
SOP	Standard operating procedure
SIV	Site opening visit
TMF	Trial Master File

Appendices

Appendix 1: Study sites and contacts

Study sites

Delta Care Network practices Geneva, Switzerland

This monitoring plan applies to all medical practices (sites) affiliated to Réseau de soins Delta Genève.

Contact information	
Study site<br>(Local principal investigator: First and last name; e-mail)	Delta Network primary care practices, Geneva<br>*Principal investigator :*<br>Dr Minette-Joëlle Zeukeng, PharmD, PhD,<br>FPH Minette.zeukeng@reseau-delta.ch<br>*Coordinator (logistics manager):*<br>Joëlle Coclet, PhD<br>Joelle.coclet@reseau-delta.ch<br>*Healthcare Manager :*<br>Cécilia Rios-Valente<br>Cecilia.rios-valente@reseau-delta.ch

Appendix 2: Scope of source data verification (SDV) and key data

According to the monitoring strategy based on low-risk and Table 3, the verification of CRF source data will be carried out as follows:

A **partial VDS** will be performed for **10% of** randomly selected trial participants according to the following table.

	CRF elements	SDV range (%)	Comments
**Visit 1 - T0**	○ Date	10%	
	○ Eligibility criteria	100%	
	○ Demographics	10%	
	○ Consent	100%	
	○ Reportable comorbidity and drug therapy in type 2 diabetes	10%	
	○ Randomization	30%	
	○ Measurements main metabolic measures (HbA1c)	100%	
	○ Measurements secondary metabolic (blood pressure, body composition)	20%	
	○ Questionnaires PROMs	5%	Reference PROMs (EQ5D, DIABQ)
**Visit 2 to 4 - T3, T6,TG**	○ Date	10%	
	○ Measurements main metabolic measures (HbA1c)	100%	
	○ Measurements secondary metabolic (blood pressure, body composition)	20%	
	○ Questionnaires PROMs	5%	Evaluation PROMs (EQ5D, DIABQ)<br>Follow-up PROMs (PHQ-9, DIAB-Q)
**Tour 5 - T12**	○ Date	10%	
	○ Measurements main metabolic measures (HbA1c)	100%	
	○ Measurements secondary metabolic (blood pressure	20%	

	CRF components	SDV range (%)	Comments
	blood pressure, body composition)		
	○ Questionnaires PROMs and PREM	5%	Evaluation PROMs (EQ5D, DIABQ)<br>PREM (experience)
	○ Activity from individualized program DT2	5%	Dietetics (individual or group)<br>Exercise/sports activities (individual or group)<br>Discussions/information (individually or in groups)
**Tour 6 - T18**	○ Date	10%	
	○ Measurements main metabolic measures (HbA1c)	100%	
	○ Measurements secondary metabolic (blood pressure, body composition)	20%	
	○ Questionnaires PROMs and PREM	5%	Evaluation PROMs (EQ5D, DIABQ)<br>PREM (experience)
**SAFETY**	○ SAE	100%	

Appendix 3 Key elements of the Alliance DT2 study protocol

	Allowance	Recruitmen t	Base line	Study period			Closing and follow-up	
**Timeline**	**Pre-recruitment**	**Pre-base line**	**t=0m[1]**	**t=3 m**	**t=6m**	**t=Gm**	**t=12m**	**t=18m**
RECRUITMENTS:								
Randomization by cluster	X							
Informed consent		X						
Evaluation of eligibility		X						
Questionnaire line basic			X					
INTERVENTIONS:								
Intervention group	X	X	X				X	X
Control group (standard practice)	X	X	X				X	X
EVALUATIONS:								
Case composition variables (e.g. age, gender, comorbidities), medication)			X					
Metabolic data *(HbA1c level, blood pressure, body composition)*			X	X	X	X	X	X
Reference and evaluation PROMs *(EQ-5D-5L, DIAB-Q)*			X				X	X
PROMs of intervention *(PHQ-S, DIAB-Q)*				X	X	X		
PREM							X	X
Program data individualized activity				X	X	X		
Billing data insurance							X[2]	X[2]

Annotations: 1. baseline assessment is performed for GI patients prior to participation in the quality circle, while for GC patients it is performed on the day of study enrolment. 2. Groupe Mutuel policyholders only. DIAB-Q: Questionnaire on diabetes intention, attitude and behavior; EQ-5D-5L = EuroQoL 5 dimensions 5 levels; HbA1c = Hemoglobin A1C; PHQ-9: Questionnaire on patient health;

PROM = Measure of patient-reported outcomes; PREM = Measure of patient-reported experience.

Appendix 4: Risk Based Monitoring Score

Risk Based Monitoring Score

SCTO PLATFORMS

Project: Alliance DT2

Author: MJ Zeukeng

Summary

Swiss risk category: A

Based on the assumptions fed into the RBM score calculator, the study risk is low and we thus recommend applying the monitoring strategy for **low-risk trials**.

Level	Number of risks
High	0
Medium	3
Low	6

The following risks are rated as high in this trial:

Category	Risk	Description

The following risks are rated as medium in this trial:

Category	Risk	Description
II. Design	Withdrawal/drop-outs	
VI. Data	Volume/complexity	
VI. Data	CRF quality	

Category	Risk	Note	Impact	Occurrence	Detectability	Score
I. Participant	Vulnerable population		0	0	0	0
I. Participant	Emergency situation		0	0	0	0
I. Participant	Complexity of consent process		0	0	0	0
II. Design	Complexity of eligibility criteria		0	0	0	0
II. Design	Complexity of design		2	1	1	2
II. Design	Complexity of primary endpoint		1	1	1	1
II. Design	Bias impacting the primary endpoint		1	1	1	1
II. Design	Additional treatment for concomitant diseases/symptoms		2	1	1	2
II. Design	Complexity of procedures		1	1	1	1
II. Design	Withdrawal/drop-outs		2	2	1	4
III. Safety	Serious drug reaction/device effect		0	0	0	0
III. Safety	Interactions		0	0	0	0
III. Safety	Participant population's conditions		0	0	0	0
IV. Intervention	Actual knowledge		0	0	0	0
IV. Intervention	Administration		0	0	0	0
IV. Intervention	Logistics		0	0	0	0
IV. Intervention	Accidental or deliberate unblinding		0	0	0	0
V. Management	Sites		0	0	0	0
V. Management	Technical requirements		0	0	0	0
V. Management	Staff requirements		2	1	1	2
VI. Data	Volume/complexity		2	2	1	4
VI. Data	CRF quality		3	3	1	9

Plan de surveillance

Formulaire basé sur le modèle de la plateforme de surveillance de la Swiss Clinical Trial Organisation (SCTO). www.sctoplatforms.ch.

PLAN DE SURVEILLANCE – Alliance DT2

Détails de l'étude	
**Titre de l'étude**	Alliance DT2
Type d'étude / Catégorie de risque (selon la loi suisse)	ClinO_Other / A
Nom de l'intervention faisant l'objet de l'enquête	Implémentation d'un programme interdisciplinaire (éducation thérapeutique du patient et questionnaires PROM/PREM) pour le traitement du diabète de type 2
Système CRF (le cas échéant)	Specify if neither secuTrial nor REDCap: SOKLE/électronique
**Promoteur**	Réseau de soins Delta
Personne de contact principale	Dr Philippe Schaller, MD, FMH, MPH<br>Administrateur général
Adresse	98 route de Chancy<br>CH - 1213 Onex
Numéro de téléphone, courriel	079 652 51 16, http://www.philippe-schaller.ch
**Investigateur principal**	Dre Minette-Joëlle Zeukeng, PharmD, PhD, FPH<br>079 892 19 81<br>minette.zeukeng@reseau-delta.ch
**Personne de contact pour la gestion des données (moniteur)**	Cécilia Rios Valente<br>Cecilia.rios-valente@reseau-delta.ch
**Personne de contact pour la gestion opérationnelle (coordinatrice)**	Joëlle Coclet, PhD<br>Joelle.coclet@reseau-delta.ch

Détails de l'approbation du plan de surveillance		
Version / Date du plan de surveillance	1	11.10.2024
**Auteur du plan de surveillance**		
Prénom et nom	Minette-Joëlle Zeukeng	
Fonction	Investigatrice principale	
Date / Signature	11.10.2024	
**Promoteur**		
Nom et prénom	Philippe Schaller	
Date / Signature	11.10.2024 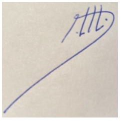	

Table des matières

1. Introduction 4
2. Stratégie de surveillance (Risk-Based Monitoring - RBM) 5
   2.1 Stratégie (tableau 1) 5
   2.2 Programmation des visites (tableau 2) 5
   2.3 Activités de surveillance (tableau 3) 6
3. Responsabilités 7
   3.1 Moniteur 7
   3.2 Promoteur 7
   3.3 Investigateurs 7
4. Activités de suivi 7
   4.1 Processus de consentement éclairé et documentation 7
   4.2 Vérification des données à la source (SDV) 8
   4.3 Sécurité 8
   4.4 Randomisation 8
   4.5 Conformité au protocole 8
   4.6 Confirmation des visites de contrôle 8
5. Visite de démarrage du site (cabinet) 8
6. Visite de contrôle de routine 9
7. Contrôle central des données 9
8. Visite de clôture 9
9. Écarts 10
Acronymes et abréviations 11
Annexes 12

1. Introduction

Les activités de contrôle sont réalisées conformément aux normes GCP. Le présent plan de surveillance est fondé sur les exigences propres à l'étude. Si les engagements convenus changent après le début de l'étude, une nouvelle version du plan de surveillance peut être publiée.

Les procédures opérationnelles standards (SOP) sont détaillées dans l'Annexe 2 du protocole de recherche Alliance DT2.

L'étude clinique Alliance DT2 se déroule dans plusieurs cabinets de médecins affiliés au Réseau Delta, ce qui lui confère une nature multicentrique. Cependant, plusieurs éléments permettent de justifier l'adoption d'un plan de surveillance similaire à celui d'un essai monocentrique, compte tenu de la structure organisationnelle spécifique et de la coordination centralisée sous la responsabilité du Réseau Delta.

Tous les cabinets impliqués dans cette étude clinique appartiennent au Réseau Delta, qui fonctionne comme une entité faîtière. Les médecins de premier recours (MPR) opèrent sous des directives uniformes et la coordination est assurée de manière centralisée par le promoteur (*Réseau de soins Delta SA*). Cette structure garantit une standardisation des pratiques au sein des différents cabinets. Il ne s'agit donc pas de sites indépendants, mais d'un réseau coordonné qui partage les mêmes procédures, type de formations et responsabilités, similaires à un centre unique avec plusieurs sous-sites.

Le Réseau Delta centralise la gestion et la coordination des différentes étapes de l'étude :

- La gestion des inclusions et consentements des patients est faite à travers une plateforme unique (*SOKLE*), sous la supervision directe de la gestionnaire en santé (GS).
- La GS et l'équipe de coordination sont responsables du suivi proactif des participants, de la collecte de données cliniques (HbA1c, questionnaires PROM/PREM), ainsi que de l'organisation des Cercles de qualité dans les différents cabinets. Cette centralisation des tâches administratives et cliniques sous une seule structure (Réseau Delta) rapproche l'organisation d'un modèle de gestion monocentrique où une équipe unique contrôle les opérations dans plusieurs lieux physiques.

L'intervention centrale de cette étude, à savoir les Cercles de qualité médecin-patient, est organisée de manière similaire dans chaque cabinet. Les procédures éducatives, le déroulement des interventions et la collecte des mesures cliniques sont standardisés selon un plan unique appliqué à tous les sites participants.

- La GS assure la coordination de la formation des médecins et l'application uniforme des protocoles d'éducation thérapeutique du patient (ETP).
- Le matériel utilisé (mesures métaboliques, questionnaires) est également standardisé et administré de la même manière, garantissant ainsi une homogénéité des pratiques, comme dans un cadre monocentrique.

L'approche de surveillance dans cette étude sera centralisée et uniformisée. Bien que les visites de contrôle (à distance) aient lieu dans plusieurs cabinets, les données sont regroupées dans un seul système (plateforme SOKLE) et les contrôles seront effectués de manière similaire dans chaque site, avec des vérifications périodiques :

- Les contrôles des données (SDV) et la gestion des écarts se feront à partir de la même plateforme, sous la supervision de la GS et l'investigatrice principale.

- La surveillance centralisée des données collectées (mesures métabolique dont HbA1c, et questionnaires PROM/PREM) se fera à des points prédéfinis (10% des participants inclus – voir détails à l'annexe 2) indépendamment du site, comme dans un contexte monocentrique où les données seraient centralisées dans une base unique.

La fréquence des visites de contrôle et leur contenu seront standardisés, avec des visites additionnelles uniquement en cas de besoin (par exemple, non-conformité majeure ou problème identifié lors de la première visite de routine). Cette approche, où tous les cabinets reçoivent des visites planifiées de manière homogène, soutient l'idée d'un modèle de surveillance similaire à celui d'un centre unique.

Finalement, le fait que la gestionnaire en santé et l'équipe d'intervention œuvrant avec tous les cabinets participants sont couverts par une assurance unique sous la faîtière du Réseau Delta renforce cette idée de gestion centralisée. Cela diminue la variabilité des pratiques et des risques à travers les différents cabinets, car ils sont tous régis par les mêmes type d'obligations contractuelles. Cette uniformité diminue les besoins d'un plan de surveillance plus détaillé pour chaque cabinet, comme cela serait nécessaire dans un essai multicentrique classique avec plusieurs promoteurs ou établissements totalement indépendants.

2. Stratégie de surveillance (Risk-Based Monitoring - RBM)

Pour les détails voir rapport du calculateur de score de risque RBM (Annexe 4)

2.1 Stratégie (tableau 1)

Stratégie de surveillance basée sur le risque recommandée selon le calculateur de score RBM de la SCTO[1]	low-risk
Facteurs de risque moyens ou élevés selon le calculateur de score RBM de la SCTO[1]	Retrait/abandon<br>Volume/complexité<br>Qualité du formulaire de rapport de cas (CRF)
Contrôle central des données ?	Oui

2.2 Programmation des visites (tableau 2)

Visite d'ouverture du site à distance (courriel au cabinet)	Avant l'inclusion du premier participant
Contrôle de routine (Visite 1)	Si nécessaire, afin d'identifier d'éventuelles non-conformités, problèmes logistiques, valeurs aberrantes.
Contrôle de routine (Visites 2 à 6)	Si nécessaire, en cas de non-conformité ou de problèmes identifiés, évènements indésirables graves.
Visite de clôture à distance	Lorsque tous les participants auront effectué leur dernière visites et que la collecte des données sera achevée.
Contrôle central des données	Lors de la création d'un nouveau Cercle de qualité médecin-patient via le logiciel SOKLE.

Au cours de l'étude, l'étendue de la vérification des données ainsi que le nombre de visites de contrôle de routine peuvent être réévalués en fonction des résultats. Le contrôleur (membre équipe investigatrice) doit informer le promoteur si du temps ou des ressources supplémentaires sont nécessaires.

2.3 Activités de surveillance (tableau 3)

**Action**	**Étendue de l'étude** (% des participants)	**Commentaires**
Vérification des formulaires de consentement éclairé	100%	
SDV complet	100%	Données métaboliques (*niveau d'HbA1c)* par trimestre durant une année puis à 6 mois sur un total de 18 mois
SDV partiel des données clés, y compris le calendrier des visites	20%	Données métaboliques (*composition corporelle, tension artérielle)*<br>*Vérification possible à partir des fichiers sources des appareils de mesure (date et heure)*
SDV partiel des données clés, y compris le calendrier des visites	10%	PROMs de référence et d'évaluation (EQ5D, DIABQ)<br>PROMs d'intervention (PHQ-9, DIAB-Q)Par trimestre<br>PREM (expérience patient)

Voir le tableau SDV de l'annexe 2 pour plus de détails sur l'étendue du SDV.

3. Responsabilités

3.1 Moniteur

Les responsabilités du contrôleur sont détaillées dans les GCP.

Le respect des exigences du protocole est important pour garantir la conduite éthique de l'étude clinique, ainsi que la validité scientifique, l'exactitude et l'exhaustivité des données produites au cours de l'étude clinique. La surveillance permet de garantir le respect de ces exigences.

Avant tout, le moniteur doit être formé par le promoteur :

- Toute documentation relative à l'étude, par exemple le protocole ou le (e)CRF
- Toute exigence spécifique en matière de contrôle, par exemple le processus de vérification des données à la source (SDV)
- Règlements et lignes directrices dans la version en vigueur : Ordonnance sur les essais cliniques dans la recherche sur l'être humain (ClinO) chapitre 4

3.2 Promoteur

Le promoteur est responsable de la mise en place et de la surveillance de l'étude. Les responsabilités du promoteur sont détaillées dans les GCP.

Tous les documents de l'étude (TMF – Trial master file) sont centralisés et accessibles sur demande.

Le promoteur est également responsable de la formation spécifique à l'étude.

La formation doit couvrir au moins les points suivants

- Formation médicale et formation à l'utilisation des terminaux
- Tous les documents spécifiques à l'étude
- Conception et procédures de l'étude
- Calendrier de l'étude
- Logistique (organisation des sessions, approvisionnement matériel pour les mesures métaboliques, etc.)
- Composition, remplissage et consignation du CRF (dans le cas d'une version papier)
- Traitement des requêtes / Délais pour le verrouillage estimé de la base de données
- Système de qualité /SOP spécifiques à l'étude

3.3 Investigateurs

Les responsabilités de l'investigatrice principale sont précisées dans les GCP.

En ce qui concerne le contrôle, l'équipe investigatrice est chargée de faciliter les activités de contrôle, de les rendre accessibles pour le contrôle et d'être disponible lors des visites de contrôle.

4. Activités de suivi

4.1 Processus de consentement éclairé et documentation

Le contrôleur (équipe investigatrice) doit vérifier que :

- Le processus de consentement est approprié, conforme aux lignes directrices internationales des bonnes pratiques cliniques (GCP et réglementations nationales suisses) et documenté dans le dossier du participant.
- Seules les versions actuelles et les dernières versions approuvées par la Commission d'éthique du formulaire d'information et de consentement du participant sont utilisées.
- Le formulaire de consentement a été signée/datée par le participant et par la personne qualifiée autorisée par l'investigatrice principale dans le registre.

- Le consentement a été obtenu et documenté correctement pour tous les participants qui ont subi une intervention dans le cadre de l'étude, conformément au registre de randomisation.
- Le formulaire original de consentement a été conservé par la gestionnaire en santé (papier et électronique) dans SOKLE. Une copie du document entièrement signé est remise au participant.
- Le consentement a été obtenu de manière appropriée avant la mise en œuvre de toute procédure liée à l'étude.
- Les registres d'inclusion et de randomisation des participants ainsi que le registre d'identification des participants sont correctement remplis et mis à jour.

4.2 Vérification des données à la source (SDV)

Le contrôleur vérifie les données du CRF par rapport aux données sources correspondantes, conformément au tableau 2.

Le contrôleur est un membre de l'équipe investigatrice.

Les divergences seront discutées avec les investigateurs et, si nécessaire, des dispositions sont prises.

4.3 Sécurité

Conformément au tableau 2, le moniteur (gestionnaire en santé) vérifie que les événements indésirables graves (EI) :

- sont correctement enregistrés conformément aux documents de base et sont cohérents avec les médicaments et les maladies concomitants
- sont signalés de manière appropriée et font l'objet d'un suivi conformément au protocole et aux exigences réglementaires

4.4 Randomisation

Le moniteur doit comparer la liste de randomisation à la nature des traitements administrés aux participants conformément au tableau 2.

4.5 Conformité au protocole

Le moniteur évaluera la conformité au protocole à chaque visite (par exemple, le moment des visites) conformément au tableau 3 et consignera tout écart dans SOKLE.

4.6 Confirmation des visites de contrôle

Toutes les visites à distance seront précédées d'un courriel ou d'une lettre de confirmation comprenant toutes les informations nécessaires concernant la visite (date, heure, lieu de la visite, documents et données de base demandés, participants, etc).

5. Visite de démarrage du site (cabinet)

Le site sera initié conformément au tableau 2.

Le contrôleur effectuera les activités suivantes :

- Rappel des principes des GCP et des exigences réglementaires
- Formation sur les éléments clé du protocole (information) et les procédures de l'étude.
- Formation sur les processus de recrutement et de consentement éclairé
- Discuter des rôles et des responsabilités de l'équipe chargée de l'étude
- Identification des documents sources (appareils de mesure métabolique et SOKLE)

- Le cas échéant, assurer la réception des formulaires requis (voir SOP, Annexe 2 du protocole Alliance DT2)

6. Visite de contrôle de routine

Les visites de contrôle de routine seront effectuées conformément au tableau 2.

Le contrôleur effectuera les activités suivantes :

- Vérifier que le processus de consentement éclairé est effectué correctement
- Vérifier que les tâches de l'étude sont effectuées par le personnel délégué
- Vérifier le respect des critères d'éligibilité
- Vérifier le respect du protocole et si toutes les déviations sont correctement signalées au promoteur (consignation dans SOKLE).
- Effectuer la vérification des données sources (SDV) sur le CRF comme spécifié à l'annexe 2.
- Identifier, discuter et résoudre les points d'action avec la collaboration du personnel du cabinet.
- Si nécessaire, revoir les inscriptions et discuter des options pour augmenter le recrutement des participants.
- Le cas échéant, vérifier les procédures de randomisation conformément à l'annexe 2, le maintien de l'aveugle.
- Le cas échéant, rassembler des documents pour le promoteur (CRF)

Après la visite, en cas de manquement, le contrôleur le documentera dans SOKLE.

7. Contrôle central des données

Les données sources des appareils pour les mesures métaboliques sont disponibles et accessible (export .xlsx ou .csv) directement à partir des appareils. Ainsi, les vérifications sont possibles à partir de l'heure et la date de la mesure contenues dans les extractions. La gestionnaire (moniteur) retranscrit les données directement dans SOKLE lors des visites.

Au niveau de SOKLE, toutes les actions (backup) peuvent être suivies et notifiées sur demande à l'administrateur SOKLE.

8. Visite de clôture

Une visite de clôture sera effectuée à distance, conformément au tableau 2.

Le contrôleur effectuera les activités suivantes :

- Résoudre les points d'action encore en suspens avec la collaboration du personnel du site.
- Veiller à ce que tous les formulaires et registres soient remplis puis signés par l'investigatrice principale.
- Informer les enquêteurs sur :
    - La responsabilité des effets indésirables graves, comme spécifié dans le protocole
    - Les exigences en matière d'archivage et de durée de conservation des documents relatifs à l'étude

Après la visite, le contrôleur rédigera si nécessaire un rapport de visite à consigner dans SOKLE.

9. Écarts

Un écart par rapport au protocole ou aux GCP est tout manquement au protocole ou aux exigences réglementaires. Le cas échéant, les écarts de protocole doivent être signalés dans le rapport de surveillance et faire l'objet d'une discussion avec l'investigateur principal.

Tous les écarts doivent être signalés par l'observateur et consigné dans SOKLE.

Tout écart critique potentiel observé au cours d'une visite de contrôle ou indépendamment des visites doit être signalé par le contrôleur au promoteur avant la rédaction du rapport de la visite de contrôle.

Exemples de questions :

- Le processus de consentement éclairé n'a pas été réalisé de manière adéquate
- Violation des critères d'inclusion/exclusion
- Affectation d'un participant au mauvais groupe d'étude
- Les exigences en matière de rapports SAE pas respectées
- Visites et suivi des participants non effectués conformément au protocole
- Tout manquement aux GCP

Acronymes et abréviations

ClinO	Ordonnance sur les essais cliniques, à l'exception des essais cliniques de dispositifs médicaux
COV	Visite de clôture
(e) CRF	Formulaire (électronique) de rapport de cas
CE	Comité d'éthique
GCP	Bonnes pratiques cliniques
HRA	Loi sur la recherche humaine
ICF	Formulaire de consentement éclairé
PI	Investigateur principal
RBM	Surveillance fondée sur les risques
RMV	Visite de contrôle de routine
SAE	Événement indésirable grave
SCTO	Organisation suisse des essais cliniques
SDV	Vérification des données sources
SOP	Procédure opérationnelle standard
SIV	Visite d'ouverture du site
TMF	Trial Master File

Annexes

Annexe 1 : Sites d'étude et contacts

Sites d'étude

Cabinet médicaux du Réseau de soins Delta Genève, Suisse

Le présent plan de surveillance est valable pour tous les cabinets médicaux (sites) affilés au Réseau de soins Delta Genève.

**Informations sur le contact**

Site de l'étude<br>(Chercheur principal local : Prénom et nom ; e-mail)	Cabinets de médecines de premiers recours du Réseau Delta, Genève<br>*Investigatrice principale :*<br>Dre Minette-Joëlle Zeukeng, PharmD, PhD, FPH<br>Minette.zeukeng@reseau-delta.ch<br>*Coordinatrice (responsable logistique):*<br>Joëlle Coclet, PhD<br>Joelle.coclet@reseau-delta.ch<br>*Gestionnaire en santé :*<br>Cécilia Rios-Valente<br>Cecilia.rios-valente@reseau-delta.ch

Annexe 2 : Étendue de la vérification des données sources (SDV) et données clés

Selon la stratégie de surveillance basée sur le risque low-risk et au tableau 3, la vérification des données sources du CRF sera effectuée comme suit :

Un **SDV partiel** sera effectué pour **10 % des** participants à l'essai sélectionnés de manière aléatoire selon le tableau suivant.

	Éléments du CRF	Étendue SDV (%)	Commentaires
**Visite 1 – T0**	○ Date	10%	
	○ Critères d'éligibilité	100%	
	○ Données démographiques	10%	
	○ Consentement	100%	
	○ Comorbidité soumises à déclaration et traitement médicamenteux lié au diabète type 2	10%	
	○ Randomisation	30%	
	○ Mesures métaboliques principale (HbA1c)	100%	
	○ Mesures métaboliques secondaires (tension artérielle, composition corporelle)	20%	
	○ Questionnaires PROMs	5%	PROMs de référence (EQ5D, DIABQ)
**Visite 2 à 4 – T3, T6,T9**	○ Date	10%	
	○ Mesures métaboliques principale (HbA1c)	100%	
	○ Mesures métaboliques secondaires (tension artérielle, composition corporelle)	20%	
	○ Questionnaires PROMs	5%	PROMs d'évalutation (EQ5D, DIABQ)<br>PROMs de suivi (PHQ-9, DIAB-Q)
**Visite 5 – T12**	○ Date	10%	
	○ Mesures métaboliques principale (HbA1c)	100%	
	○ Mesures métaboliques secondaires (tension	20%	

	Éléments du CRF	Étendue SDV (%)	Commentaires
	artérielle, composition corporelle)		
	○ Questionnaires PROMs et PREM	5%	PROMs d'évalutation (EQ5D, DIABQ)<br><br>PREM (expérience)
	○ Activité du programme individualisé DT2	5%	Diététique (en individuel ou en groupe)<br><br>Mise en mouvement/activités sportive (en individuel ou en groupe)<br><br>Discussions/information (en individuel ou en groupe)
**Visite 6 – T18**	○ Date	10%	
	○ Mesures métaboliques principale (HbA1c)	100%	
	○ Mesures métaboliques secondaires (tension artérielle, composition corporelle)	20%	
	○ Questionnaires PROMs et PREM	5%	PROMs d'évalutation (EQ5D, DIABQ)<br><br>PREM (expérience)
**SÉCURITÉ**	○ SAE	100%	

Annexe 3 Éléments clés du protocole d'étude Alliance DT2

	**Allocation**	**Recrutement**	**Ligne de base**	**Période de l'étude**			**Clôture et suivi**	
**Chronologie**	**Pré-recrutement**	**Pré-ligne de base**	**t=0m[1]**	**t=3m**	**t=6m**	**t=9m**	**t=12m**	**t=18m**
RECRUTEMENTS:								
Randomisation par cluster	X							
Consentement éclairé		X						
Évaluation de l'éligibilité		X						
Questionnaire de ligne de base			X					
INTERVENTIONS:								
Groupe d'intervention	X	X	X ●	—	—	●	X	X
Groupe contrôle (pratique standard)	X	X	X				X	X
ÉVALUATIONS:								
Variables de composition des cas (par ex., âge, sexe, comorbidités, médication)			X					
Données métaboliques *(niveau d'HbA1c, pression artérielle, composition corporelle)*			X	X	X	X	X	X
PROMs de référence et d'évaluation *(EQ-5D-5L, DIAB-Q)*			X				X	X
PROMs d'intervention *(PHQ-9, DIAB-Q)*				X	X	X		
PREM							X	X
Données du programme d'activité individualisé				X	X	X		
Données de facturation des assurances							X[2]	X[2]

Annotations : 1. L'évaluation de base est effectuée pour les patients du GI avant leur participation au cercle de qualité, tandis que pour les patients du GC, elle se fait le jour de l'inscription à l'étude. 2. Seulement pour les assurés du Groupe Mutuel. DIAB-Q : Questionnaire sur l'intention, l'attitude et le comportement en matière de diabète ; EQ-5D-5L = EuroQoL 5 dimensions 5 niveaux ; HbA1c = Hémoglobine A1C ; PHQ-9 : Questionnaire de santé du patient ; PROM = Mesure des résultats rapportés par les patients ; PREM = Mesure de l'expérience rapportée par les patients.

Risk Based Monitoring Score

SCTO PLATFORMS

Project: Alliance DT2

Author: MJ Zeukeng

Summary

Swiss risk category: A

Based on the assumptions fed into the RBM score calculator, the study risk is low and we thus recommend applying the monitoring strategy for **low-risk trials**.

Level	Number of risks
High	0
Medium	3
Low	6

The following risks are rated as high in this trial:

Category	Risk	Description

The following risks are rated as medium in this trial:

Category	Risk	Description
II. Design	Withdrawal/drop-outs	
VI. Data	Volume/complexity	
VI. Data	CRF quality	

Category	Risk	Note	Impact	Occurrence	Detectability	Score
I. Participant	Vulnerable population		0	0	0	0
I. Participant	Emergency situation		0	0	0	0
I. Participant	Complexity of consent process		0	0	0	0
II. Design	Complexity of eligibility criteria		0	0	0	0
II. Design	Complexity of design		2	1	1	2
II. Design	Complexity of primary endpoint		1	1	1	1
II. Design	Bias impacting the primary endpoint		1	1	1	1
II. Design	Additional treatment for concomitant diseases/symptoms		2	1	1	2
II. Design	Complexity of procedures		1	1	1	1
II. Design	Withdrawal/drop-outs		2	2	1	4
III. Safety	Serious drug reaction/device effect		0	0	0	0
III. Safety	Interactions		0	0	0	0
III. Safety	Participant population's conditions		0	0	0	0
IV. Intervention	Actual knowledge		0	0	0	0
IV. Intervention	Administration		0	0	0	0
IV. Intervention	Logistics		0	0	0	0
IV. Intervention	Accidental or deliberate unblinding		0	0	0	0
V. Management	Sites		0	0	0	0
V. Management	Technical requirements		0	0	0	0
V. Management	Staff requirements		2	1	1	2
VI. Data	Volume/complexity		2	2	1	4
VI. Data	CRF quality		3	3	1	9
